# Supplementary material for: A nanoparticle-based sonodynamic therapy reduces Helicobacter pylori infection in mouse without disrupting gut microbiota
Source: Nat Commun. 2024 Jan 29;15:844. doi: 10.1038/s41467-024-45156-8 (PMC10825188; doi:10.1038/s41467-024-45156-8)
Supplement: Supplementary file 1 — Supplementary Information [file 41467_2024_45156_MOESM1_ESM.pdf]

Supplementary Information

A nanoparticle-based sonodynamic therapy reduces *Helicobacter pylori* infection in mouse without disrupting gut microbiota

Tao Liu[1,2,3], *Shuang Chai*[1,2,3], *Mingyang Li*[1,2,3], *Xu Chen*[1,2,3], *Yutao Xie*[1,2,3], *Zehui Zhao*[1,2,3], *Jingjing Xie*[1,2,3], *Yunpeng Yu*[1,2,3], *Feng Gao*[1], *Feng Zhu*[4], *Lihua Yang*[1,2,3]*

[1]Hefei National Research Center for Physical Sciences at the Microscale, [2]CAS Key Laboratory of Soft Matter Chemistry, [3]School of Chemistry and Materials Science, [4]Division of Life Science and Medicine, University of Science and Technology of China, Hefei, Anhui 230026 China.

*Corresponding author: (L.Y.) lhyang@ustc.edu.cn

ADDITIONAL RESULTS AND DISCUSSION

1. PLGA@Lecithin nanoparticles were successfully prepared.

A lecithin bilayer-coated PLGA nanoparticle (PLGA@Lecithin) was prepared (Supplementary Fig. 1a) as a model for lipid bilayer-coated nanoparticles. Under transmission electron microscopy (TEM), PLGA@Lecithin appeared spherical, similar to its uncoated precursor, PLGA nanoparticles, but in contrast to the precursor PLGA nanoparticles, it acquired a core-shell structure (Supplementary Fig. 1b,c). Through the statistical analysis of TEM images, the number of nanoparticles with a membrane coating rate of ≥80% accounted for 64.7% of the total number of nanoparticles (Supplementary Fig. 1d), indicating that the lecithin membrane could be well coated on the surface of the nanoparticles to form a phospholipid bilayer. Moreover, after the lecithin membrane coating, the nanoparticle size (in average hydrodynamic diameter) increased from 151.1 nm for the precursor PLGA nanoparticles to 167.8 nm for PLGA@Lecithin (Supplementary Fig. 1e), indicative of an increase of 16.7 nm in average hydrodynamic diameter, which is consistent with the previously reported increase in size after lecithin membrane coating[1-3]. In addition, the average zeta potential (ζ) of PLGA@Lecithin was -30.3 mV, which is much closer to that of lecithin vesicles (-31.5 mV) than to that of bare PLGA nanoparticles (-27.7 mV) (Supplementary Fig. 1e). Taken together, these results indicate successful preparation of PLGA@Lecithin, in which a lecithin membrane is successfully coated over the precursor PLGA nanoparticle.

2. PLGA@PEG nanoparticles were successfully prepared.

PLGA@PEG nanoparticles were prepared (Supplementary Fig. 2a) as a model for lipid-free PEGylated nanoparticles. Under TEM, the as-prepared PLGA@PEG nanoparticles appeared to be

spherical (Supplementary Fig. 2b). Dynamic light scattering (DLS) results revealed an average hydrodynamic diameter of 196.3 nm (Supplementary Fig. 2c) and a zeta potential (ζ) of -35.8 mV (Supplementary Fig. 2d) for PLGA@PEG.

3. USS-PLGA@Lecithin nanoparticles were successfully prepared.

The USS-PLGA@Lecithin nanoparticles (*i.e.*, Ver-PLGA@Lecithin, Ce6-PLGA@Lecithin, or ICG-PLGA@Lecithin) were prepared by preloading a USS (*i.e.*, verteporfin (Ver), chlorin e6 (Ce6), or indocyanine green (ICG)) into PLGA nanoparticles and then coating the resultant USS-PLGA nanoparticles with a lecithin bilayer membrane, which yielded the expected USS-PLGA@Lecithin nanoparticles (Supplementary Fig. 8a, 9a and 10a).

Under TEM, the as-prepared USS-PLGA@Lecithin particles appeared spherical, as did the precursor USS-PLGA nanoparticles; nevertheless, unlike the USS-PLGA nanoparticles, the USS-PLGA@Lecithin particles exhibited a core-shell structure (Supplementary Fig. 8b,c; Supplementary Fig. 9b,c and Supplementary Fig. 10b,c). Moreover, DLS characterizations revealed that, due to the lecithin membrane coating, the average hydrodynamic diameters of the nanoparticles increased relatively by 15.0-21.7 nm, from 181.2 nm for the Ver-PLGA nanoparticles to 196.2 nm for Ver-PLGA@Lecithin (relatively by 15.0 nm) (Supplementary Fig. 8d), from 172.5 nm for the Ce6-PLGA nanoparticles to 191.7 nm for Ce6-PLGA@Lecithin (relatively by 19.2 nm) (Supplementary Fig. 9d), and from 176.2 nm for the ICG-PLGA nanoparticles to 197.9 nm for ICG-PLGA@Lecithin (relatively by 21.7 nm) (Supplementary Fig. 10d). Notably, such relative increases in average size are consistent with a previously reported increase in average hydrodynamic diameter after lecithin membrane coating[1-3]. In addition, according to DLS characterizations, the USS-PLGA@ Lecithin particles unanimously exhibited average zeta

potentials (ζ) (which are -36.8 mV for Ver-PLGA@Lecithin, -37.7 mV for Ce6-PLGA@Lecithin, and -38.1 mV for ICG-PLGA@Lecithin) close to that of the precursor lecithin vesicle (-37.2 mV), rather than those of their corresponding precursor USS-PLGA nanoparticles (which are -26.2 mV for Ver-PLGA nanoparticles, -25.1 mV for Ce6-PLGA nanoparticles, and -25.9 mV for ICG-PLGA nanoparticles) (Supplementary Fig. 8d, 9d and 10d). Collectively, these results indicate successful coating of the USS-PLGA nanoparticles with lecithin bilayer membrane and consequently suggest successful preparation of the expected USS-PLGA@Lecithin nanoparticles.

4. The ROS generated by USS-PLGA@Lecithin upon US exposure is singlet oxygen ($^1O_2$).

Some ROS species are extremely reactive, while others are poor oxidants[4]. Being extremely reactive, singlet oxygen ($^1O_2$), hydroxyl radical (•OH) and superoxide anion ($O_2^{-\bullet}$) are highly cytotoxic and hence present the three major ROS species that most ROS-generating materials are expected to produce. Singlet oxygen sensor green (SOSG) is weakly blue fluorescent, but upon oxidation by $^1O_2$, it becomes the brightly green fluorescent SOSG-EP (an endoperoxide of SOSG)[5]. Using SOSG as the $^1O_2$ probe (Fig. 3a), we found that upon US exposure (at 0.5 W/cm^2 for 10 min), all three USS-PLGA@Lecithin nanoparticles rendered SOSG solution exhibit significantly increased green fluorescence intensity in a USS dose-dependent manner (Fig. 3f and Supplementary Fig. 11e, 12e, and 13e), indicative of $^1O_2$ generation due to the preloaded USS. Consistently, $^1O_2$ generation by these three USS-PLGA@Lecithin nanoparticles was confirmed with electron spin resonance spectroscopy (ESR) (Supplementary Fig. 11h, 12 h, and 13 h). Dihydroethidium (DHE) is weakly blue fluorescent that, upon oxidation by $O_2^{-\bullet}$, becomes brightly red fluorescent[6]. Pure phthalic acid (PTA) is virtually nonfluorescent, but upon oxidation by •OH, it becomes brightly fluorescent[7]. To assess whether the USS-PLGA@Lethicin nanoparticles

generate $O_2^{-\bullet}$ and •OH upon US exposure, we used DHE and PTA as the respective probes for $O_2^{-\bullet}$ and •OH but failed to observe appreciable changes in their fluorescence emission spectra under US irradiation (at 0.5 W/cm^2 for 10 min) (Supplementary Figs. 11-13), suggesting the absence of $O_2^{-\bullet}$ or •OH generation. Obviously, upon US exposure, all three USS-PLGA@Lecithin nanoparticles unanimously generate $^1O_2$, but neither $O_2^{-\bullet}$ nor •OH (Fig. 3f, Supplementary Figs. 11-13). Of note, in the order of decreasing efficiency in $^1O_2$ generation, these three USS-PLGA@Lecithin nanoparticles are ranked as Ce6-PLGA@Lethicin, Ver-PLGA@Lecithin, and ICG-PLGA@Lecithin (Fig. 3f), which mirrors that in ROS generation, confirming $^1O_2$ as the ROS generated.

5. Ver-PLGA@Lecithin is intrinsically biocompatible.

All three components of Ver-PLGA@Lecithin have been approved by the Food and Drug Administration (FDA) of the United States of America (USA) for clinical use[8-10], suggesting intrinsic biocompatibility for this nanosensitizer. This is indeed the case, according to *in vivo* studies both in healthy mouse models (Supplementary Fig. 16) and in gastric *H. pylori*-infected mice (Supplementary Fig. 17 and 18).

A drop in host body weight usually indicates acute or high toxicity. When orally administered alone into healthy mouse models, Ver-PLGA@Lecithin failed to cause a detectable decrease in average mouse weight throughout the whole observation window (Supplementary Fig. 16b), indicative of a lack of acute toxicity. TNF-α, IL-6 and IL-1β are proinflammatory cytokines whose overproduction usually indicates the occurrence of certain inflammatory and autoimmune disorders and even cancer development[11]. Of note, in mice administered orally with Ver-PLGA@Lecithin (in PBS), the serum levels of TNF-α, IL-6 and IL-1β were similar to those in

mice treated with PBS (Supplementary Fig. 16c-e), indicating a lack of proinflammatory responses after oral administration of Ver-PLGA@lecithin. To examine whether oral administration of Ver-PLGA@Lecithin results in damage to the stomach and the five major organs (namely, heart, liver, spleen, lung, and kidney), we performed histological analysis on hematoxylin and eosin (H&E)-stained tissues of the stomach and the major organs collected after treatment completion (Supplementary Fig. 16a) and found undetectable abnormalities or lesions in mice treated with Ver-PLGA@Lecithin (in PBS) compared to the untreated controls (*i.e.*, treated with PBS) (Supplementary Fig. 16f), indicative of a lack of appreciable damage to the stomach or major organs.

To examine whether orally administered Ver-PLGA@Lecithin alone is harmless to the gut microbiota, we treated gastric *H. pylori*-infected mouse models similar to those used for sonodynamic therapy but orally administered Ver-PLGA@Lecithin nanoparticles alone (*i.e.*, without US exposure), their healthy counterparts (*i.e.*, the healthy group) and those treated with PBS alone (*i.e.*, the control group) for comparison and collected mouse feces at 48 h after treatment for 16S rRNA analysis (Supplementary Fig. 17a and Supplementary Fig. 18a). Our results reveal that the Ver-PLGA@Lecithin group exhibited similar α diversity of gut microbiota as both the healthy and control groups (Supplementary Fig. 17b and Supplementary Fig. 18b), indicative of negligible effects of Ver-PLGA@Lecithin on the α diversity of gut microbiota. Principal component analysis (PCA) on the β diversity of gut microbiota found that the location of the Ver-PLGA@Lecithin group almost overlapped with those of the control and healthy groups (Supplementary Fig. 17c and Supplementary Fig. 18c), indicative of undetectable alteration to the flora structure of gut microbiota after oral administration of Ver-PLGA@Lecithin alone. In addition, to understand how oral administration of Ver-PLGA@Lecithin alone affects the gut

microbiota composition, we quantified the relative abundances of gut bacterial species (Supplementary Fig. 17d, e and Supplementary Fig. 18d, e) and found that at both the phylum and genus levels, the Ver-PLGA@Lecithin group exhibited a pattern nearly identical to those of the "healthy" and "control" groups.

To further determine changes in the composition of the gut microbiota, we performed statistical analysis on the difference between the Ver-PLGA@Lecithin group *and* the healthy group (Supplementary Fig. 17f, g) and between the Ver-PLGA@Lecithin group *and* the control group (Supplementary Fig. 17h, i). First, the change in the relative abundance of gut bacterial species after Ver-PLGA@Lecithin treatment was indicated as the fold change (*i.e.*, "$\log_2$(Ver-PLGA@Lecithin/Healthy)" and "$\log_2$(Ver-PLGA@Lecithin/Control)") (Supplementary Fig. 17f, h). For a change in bacterial relative abundance after Ver-PLGA@Lecithin treatment to be considered significant, it needs to be ≥2 or ≤1/2 in the fold of change, and its *P* value needs to be <0.05. Our plots of $-\log_{10}$(*P* value) *versus* the fold change in bacterial relative abundance (Supplementary Fig. 17f, h) revealed that only 2 bacterial species were downregulated (namely, *Actinobacteria* at the phylum level and *unclassified Erysipelotrichaceae* at the genus level), while no specified species were upregulated, and this is the case regardless of whether the reference was the healthy group or the control group. Second, the change in relative abundance of gut bacterial species after Ver-PLGA@Lecithin treatment was indicated as the absolute value of change (*i.e.*, Relative Abundance (Ver-PLGA@Lecithin-Healthy, %)and Relative Abundance (Ver-PLGA@Lecithin-Control, %)) (Supplementary Fig. 17g,i). For bacteria to be marked as significantly perturbed in the plots of $-\log_{10}$(*P* value) *versus* the absolute change in bacterial relative abundance, the *P* value needs to be <0.05. Our plots of $-\log_{10}$(*P* value) *versus* the absolute value of change in bacterial relative abundance ("Relative Abundance (Ver-PLGA@Lecithin -

Healthy, %)" and "Relative Abundance (Ver-PLGA@Lecithin - Control, %)") (Supplementary Fig. 17g,i) revealed that only 2 bacterial species were downregulated (namely, *Actinobacteria* at the phylum level in trial 2 and *unclassified_Erysipelotrichaceae* at the genus level), while 3 specific species were upregulated (namely, *Actinobacteria* at the phylum level in trial 1 and *Enterococcus* and *Bacteroidetes* at the genus level). Clearly, oral administration of Ver-PLGA@Lecithin alone conferred only negligible effects on the gut microbiota.

Collectively, these results suggest intrinsic biocompatibility for Ver-PLGA@Lecithin and support its use *in vivo*.

6. Effects on apoptotic death of gastric cells after sonodynamic therapy *versus* triple therapy.

For a TUNEL-stained image, the percentage of green fluorescence intensity relative to the intensities of both green and blue fluorescence signals was calculated with the following function:

$$\text{percentage of green fluorescence intensity} = I_{Green} / (I_{Green} + I_{Blue})$$

where I_{Green} and I_{Blue} represent the mean intensity of green fluorescence and the mean intensity of blue fluorescence, respectively.

TUNEL-stained stomach tissue samples from each treatment group were examined at high magnification and at 50 different fields of view, which enabled us to perform statistical analysis on the percentage of green fluorescence intensity relative to the intensities of both green and blue fluorescence (Supplementary Fig. 19). As green fluorescence indicates TUNEL-positive (*i.e.*, apoptotic) cells and blue fluorescence indicates TUNEL-negative cells, the as-calculated percentage of green fluorescence intensity for one image indicates the ratio of TUNEL-positive cells relative to all cells in that image. Therefore, the average of the percentages of green fluorescence intensity calculated over the 50 images of one treatment group indicates the average

ratio of TUNEL-positive cells relative to all imaged cells for that treatment group.

Our results show that, for the healthy group, the percentages of green fluorescence intensity were within the range of 0.48-1.54%, which indicates the percentage of apoptotic cells in the normal stomach due to normal cell turnover. In the TUNEL-stained images, the control group exhibited percentages of green fluorescence intensity (0.79-2.14%) significantly higher than the healthy group ($P < 0.0001$), indicative of increased cell apoptosis due to gastric *H. pylori* infection. To our surprise, the triple therapy group exhibited further higher percentages of green fluorescence intensity (2.56-4.56%, $P < 0.0001$), suggesting deterioration rather than mitigation of cell apoptosis after *H. pylori* eradication with triple therapy, possibly due to triple therapy itself and its neglect of VacA. In stark contrast to triple therapy, the sonodynamic therapy (*i.e.*, Ver-PLGA@Lecithin + US) and Ver-PLGA@Lecithin alone (*i.e.*, the Ver-PLGA@Lecithin group) both reduced the percentages of green fluorescence intensity to levels (0.58-2.95% and of 0.39-2.18%, respectively) comparable to those of the healthy group ($P > 0.3$), indicative of renormalized cell apoptosis in stomach tissues, possibly owing to the ability of Ver-PLGA@Lecithin to neutralize VacA (Fig. 2). Nevertheless, after ultrasound exposure alone, the percentages of green fluorescence intensity (0.86-3.20%) were comparable to those of the healthy group ($P < 0.0001$), indicative of the inability of US exposure alone to renormalize cell apoptosis in stomach tissues. Collectively, these results suggest that the sonodynamic therapy mediated by Ver-PLGA@Lecithin is able to renormalize the cell apoptosis level in stomach tissue possibly owing to the ability of Ver-PLGA@Lecithin to remove VacA (Figure 2), which again underscores the importance of VacA removal in gastric *H. pylori* infection treatment.

7. Analysis on the perturbation to gut microbiota composition using the absolute difference

in bacterial relative abundance.

We further analysed the sonodynamic therapy's effects on mouse gut microbiota composition, by gauging the difference in bacterial relative abundance with the absolute change in bacterial relative abundance and plotting $-\log_{10}(P$ value) *versus* the absolute change in bacterial relative abundance (*i.e.*, Relative Abundance (Sonodynamic Therapy – Healthy, %) when compared to the healthy group, and Relative Abundance (Sonodynamic Therapy – Control, %) when compared to the control group) (Supplementary Fig. 21a,b). In the resultant plots of $-\log_{10}(P$ value) *versus* the absolute change in bacterial relative abundance, a bacterial species was marked as a significantly up- or downregulated one as long as the P value associated with its difference in relative abundance is <0.05. Consistently observed in both trials 1 and 2, the sole significant impact which the sonodynamic therapy introduced to mouse gut microbiota composition is the upregulation of *Lactobacillus* and this is the case no matter whether the reference is the healthy group or the control group (Supplementary Fig. 21a,b). Only in trial 1, the sonodynamic therapy significantly downregulated *Turicibacter* no matter whether the reference is the healthy group or the control group (Supplementary Fig. 21a,b). Only in trial 1, the sonodynamic therapy impacted the relative abundance of *Clostridium_sensu_stricto* as compared to the healthy group (Supplementary Fig. 21a) and that of *unclassified_Lachnospiraceae* as compared to the control group (Supplementary Fig. 21b). Only in trial 2, the sonodynamic therapy impacted the relative abundances of *Staphylococcus* and *Enterococcus* as compared to the control group (Supplementary Fig. 21b). It should be noted that the differences in relative abundance of *Staphylococcus*, *Enterococcus*, and *unclassified_Lachnospiraceae* are boarding on the edge of 0% (Supplementary Fig. 21b). Similarly, we analysed the triple therapy's effects on mouse gut microbiota composition by plotting of $-\log_{10}(P$ value) *versus* the absolute change in bacterial relative abundance (*i.e.*, Relative Abundance

(Triple Therapy – Healthy, %) when compared to the healthy group, and Relative Abundance (Triple Therapy – Control, %) when compared to the control group) (Supplementary Fig. 21c,d). Unlike the sonodynamic therapy, triple therapy significantly downregulated one bacterial species at the phylum level (specifically, it is *Actinobacteria*) and eight bacterial species at the genus level (specifically, they are *Lactobacillus*, *Turicibacter*, *Parabacteroides*, *Romboutsia*, *Bifidobacterium*, *unclassified_Porphyromonadaceae*, *unclassified_clostridiales*, *unclassified_Erysipelotrichaceae*) while significantly upregulated one bacterial species at the phylum level (specifically, it is *Proteobacteria*) and seven bacterial species at the genus level (specifically, they are *Flavonifractor*, *Clostridium_XlVa*, *Bacteroides*, *Parasutterella,* *Escherichina_Shigella*, *Enterococcus*, *unclassified_Lachnospiraceae*), and this is the case no matter whether the reference is the healthy group (Supplementary Fig. 21c) or the control group (Supplementary Fig. 21d). Collectively, these observations based on the absolute change in bacterial relative abundance indicate that, unlike triple therapy, the sonodynamic therapy mediated by Ver-PLGA@Lecithin left the upregulation of *Lactobacillus* as the sole significant perturbation to mouse gut microbiota composition, consistent with the results based on the fold of change in bacterial relative abundance (Fig. 7f-i).

8. Effects of host size on the α diversity of gut microbiota after sonodynamic therapy.

The α diversity of the gut microbiota in mice after sonodynamic therapy was slightly lower than that in their healthy counterparts or that in their untreated (*i.e.*, control) counterparts, likely because the C57BL/6J mice, the animal models we used here, are relatively so small compared to the size of the ultrasound probe we used that it was difficult for us to distinguish the location of their stomach from that of the neighboring part of their intestinal tracts (Supplementary Fig. 23).

Due to the large diameter of the ultrasound probe (d = 5 cm), it is difficult to spatially

distinguish the intestine and stomach of the C57BL/6J mice used in the experiment. Nevertheless, this problem can be solved when the sonodynamic therapy is applied to clinical treatment, as the medical ultrasound probes currently available in clinics can easily achieve spatial selectivity between the intestine and stomach in the human body. Therefore, the sonodynamic therapy is reasonably expected to be safe for the gut microbiota in humans.

9. Effects of gastric *H. pylori* infection on mouse gut microbiota.

Analysis of the α diversity of mouse gut microbiota reveals that the control group exhibited similar α diversity as the healthy group (with *P* values of 0.9931 and 0.8666 in trials 1 and 2, respectively) (Fig. 7b and Supplementary Fig. 20b), suggesting lack of significant change to the α diversity of mouse gut microbiota after *H. pylori* infection alone. Moreover, principal component analysis (PCA) on the β diversity of mouse gut microbiota shows that the location of the control group overlapped with that of the healthy group (Fig. 7c and Supplementary Fig. 20c), suggesting lack of significant change to the β diversity of mouse gut microbiota after *H. pylori* infection alone. In addition, quantification on the relative abundances of commensal bacteria in mouse gut indicates that, at both the phylum and genus levels, the control group exhibited similar patterns of relative abundance for gut commensal bacteria as the healthy group (Fig. 7d, e and Supplementary Fig. 20d, e).

To further examine whether there are commensal bacterial species whose relative abundances in mouse gut microbiota changed significantly after *H. pylori* infection, we analysed the difference in bacterial relative abundance between the control group and the healthy group (Supplementary Fig. 24), by gauging the change in bacterial relative abundance after *H. pylori* infection with the absolute change (*i.e.*, Relative Abundance (Control-Healthy), %)) or the fold of change (*i.e.*,

log_2(Control/Healthy)) in bacterial relative abundance. The plots of -log_{10}(*P* value) *versus* Relative Abundance (Control-Healthy), %) (Supplementary Fig. 24a,c) revealed only 1 significantly perturbed gut commensal bacterium at the genus level (which is *unclassified_Clostridiales*) (Supplementary Fig. 24c) while none at the phylum level (Supplementary Fig. 24a). Similarly, the plots of -log_{10}(*P* value) *versus* log_2(Control/Healthy) (Supplementary Fig. 24b,d) revealed only 1 significantly perturbed gut commensal bacterium at the genus level (which is, again, *unclassified_Clostridiales*) (Supplementary Fig. 24d) while none at the phylum level (Supplementary Fig. 24b). Specifically, *unclassified_Clostridiales* is down-regulated, no matter whether the change in bacterial relative abundance after *H. pylori* infection is gauged with the fold of change or the absolute change (Supplementary Fig. 24). Collectively, these results indicate that the down-regulation of *unclassified_Clostridiales* is the sole significant change to mouse gut microbiota composition after *H. pylori* infection.

Results above show that *H. pylori* infection did not impact the α or β diversities of mouse gut microbiota and left the down-regulation of *unclassified_Clostridiales* as the sole significant change to mouse gut microbiota composition, which contrasts significantly with the previously reported changes to human gut microbiota due to *H. pylori* infection[12-16] (Supplementary Data 2). The difference between the observations in this work and those in previous relevant studies on human objects may arise because of the following two reasons.

First, the current clinical tests for *H. pylori* infection[17], though able to indicate whether a human subject is *H. pylori*-positive or -negative, cannot indicate for-how-long a human subject has been infected for *H. pylori*-positive cases; in contrast, in animal studies, the length-of-time between the time-point of *H. pylori* inoculation and that of gut microbiota sampling is readily controllable. More importantly, using murine models, examinations on fecal samples collected at

different months after *H. pylori* infection show that, although the microbial community structures between infected subjects and naïve controls are similar within 1 month after *H. pylori* infection, they become more and more divergent as the post-infection observation window extends[18]. In this work, mouse fecal samples were collected on day 23 after the *H. pylori* inoculation (Supplementary Figure 20a), which is within 1 month after *H. pylori* infection and may explain why *H. pylori* infection did not impact the α or β diversities of mouse gut microbiota but left down-regulation of *unclassified_Clostridiales* as the sole significant change to mouse gut microbiota composition.

Second, regarding the specific impact of gastric *H. pylori* infection to human gut microbiota, controversy exists in the previous relevant studies on human objects[16]. For example, some studies observed non-significant difference in α-diversity of gut microbiota between *H. pylori*-positive patients and *H. pylori*-negative controls[14,19-22], while others showed significantly higher α-diversity for *H. pylori*-positive patients than *H. pylori*-negative controls[12,13,15,23]. Similarly, some studies show no difference in β-diversity between *H. pylori*-positive patients and *H. pylori*-negative controls while others find "β-diversity differs"[16] likely because β-diversity varies significantly even within the group of *H. pylori*-positive human subjects depending on individual *H. pylori* load determined by stool antigen test[13]. Moreover, the specific bacterial commensals (phyla or genera) that show differences in relative abundance in gut microbiota between *H. pylori*-positive patients and *H. pylori*-negative controls vary in different studies[16]. Such controversy arises likely because *H. pylori* infection may not be the only health disorder the human subjects involved in some studies have. For example, children with gastritis but tested *H. pylori*-negative exhibit significantly different gut microbiota composition as compared with their healthy controls and gastric *H. pylori* infection aggravates the gut microbiota dysbiosis in children with gastritis[20]. For

H. pylori-infected human subjects, those with severe atrophic gastritis exhibit significantly higher relative abundance of Lactobacillus in gut microbiota than counterparts with mild or no atrophic gastritis[24], indicating influence of the severity of atrophic gastritis on the relative abundance of Lactobacillus in gut microbiota. In addition, one prior study shows increased Bacteroidetes-to-Firmicutes (B:F) ratio in gut microbiota for *H. pylori*-positive patients after triple therapy even at 3 months after the therapy as compared to that before the therapy[25] while another one reveals decreased B:F ratio for *H. pylori*-positive young adults after antibiotic-based eradication therapy[26], and this difference arises likely because of the difference in age and race between the human subjects involved in these two studies[25].

Supplementary Figures

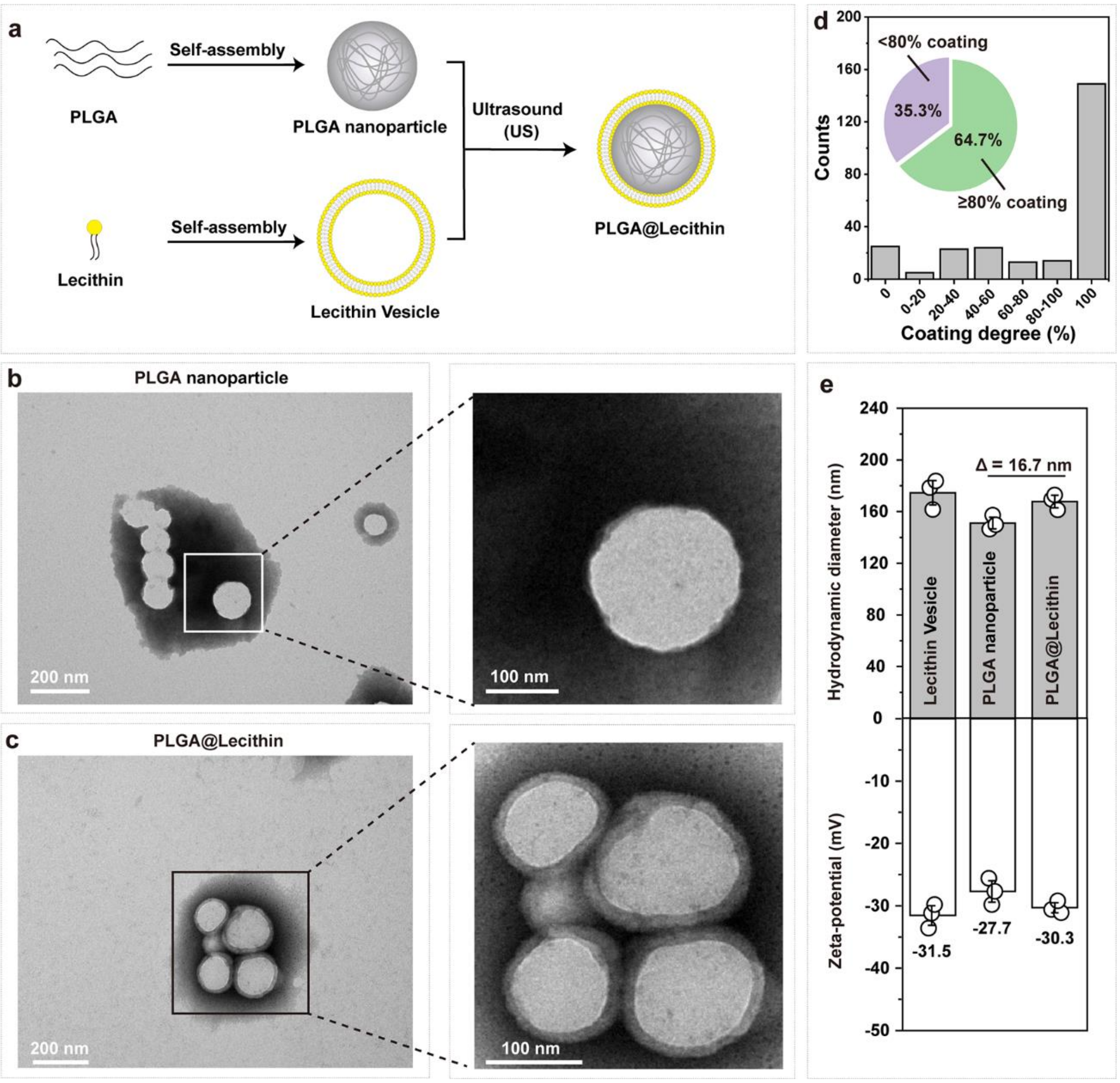

Supplementary Figure 1. PLGA@Lecithin preparation and characterization. a. Schematic illustration of the preparation of PLGA@Lecithin nanoparticles. **b-c.** Transmission electron microscopy (TEM) images of (b) PLGA nanoparticles and (c) PLGA@Lecithin nanoparticles. Two times, each experiment was repeated independently with similar results. **d.** Counts of PLGA@Lecithin nanoparticles with varying lecithin membrane coating degrees among a total of 252 individual PLGA@Lecithin nanoparticles in TEM images. The pie chart (inset) indicates that

the percentages of PLGA@Lecithin nanoparticles with membrane coating degrees of ≥80% and <80% were 64.7% and 35.3%, respectively. **e.** The hydrodynamic diameters and zeta potentials of PLGA@Lecithin in Millipore water, with those of the precursor PLGA nanoparticle and lecithin vesicle included for comparison. Bar heights are reported as the average ± standard deviation (n = 3 independent experiments). Source data are provided as a Source Data file.

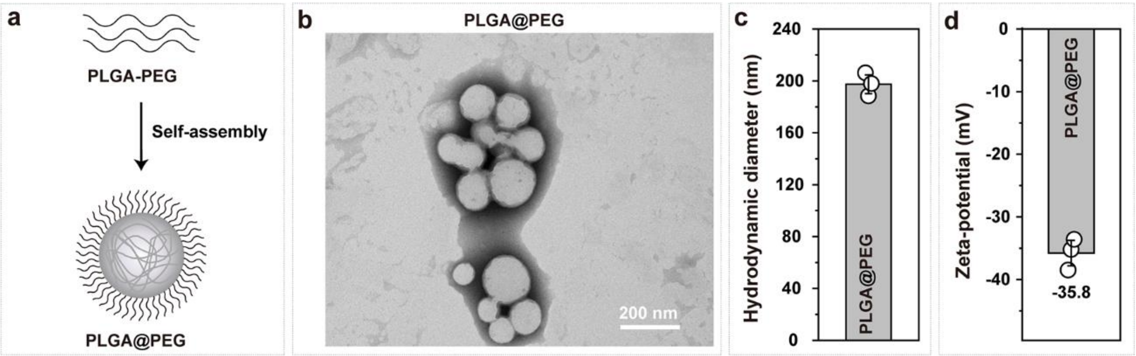

Supplementary Figure 2. PLGA@PEG preparation and characterization. a. Schematic illustration of the preparation of PLGA@PEG nanoparticles. **b.** TEM images of PLGA@PEG nanoparticles. Two times, each experiment was repeated independently with similar results. **c-d.** The hydrodynamic diameter and zeta potential of PLGA@PEG nanoparticles in Millipore water. Bar heights are reported as the average ± standard deviation (n = 3 independent experiments). Source data are provided as a Source Data file.

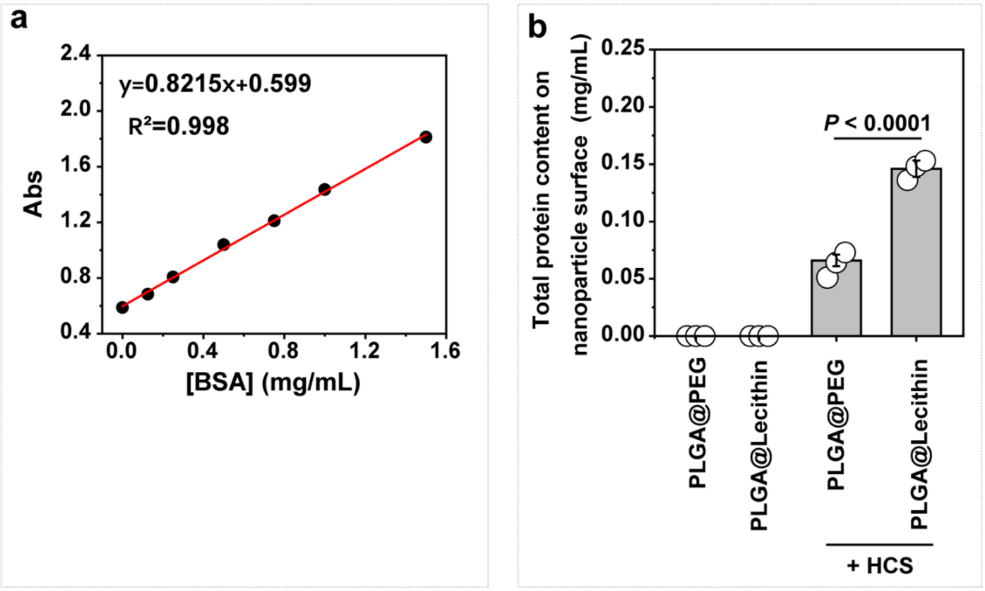

Supplementary Figure 3. Quantifying adsorbed proteins on nanoparticles. a. Standard curve measured through a Bradford Protein Assay Kit using BSA as a protein standard. **b.** Contents of total adsorbed proteins on nanoparticles (PLGA@PEG or PLGA@Lecithin) (10 mg/mL) recollected after 12 h incubation in HCS-supplemented water (10 mg/mL), with those treated similarly but in water included for comparison. Bar heights are reported as the average ± standard deviation (n = 3 independent experiments). Statistical analysis was carried out with a one-way ANOVA with Tukey's multiple-comparison test. Source data are provided as a Source Data file.

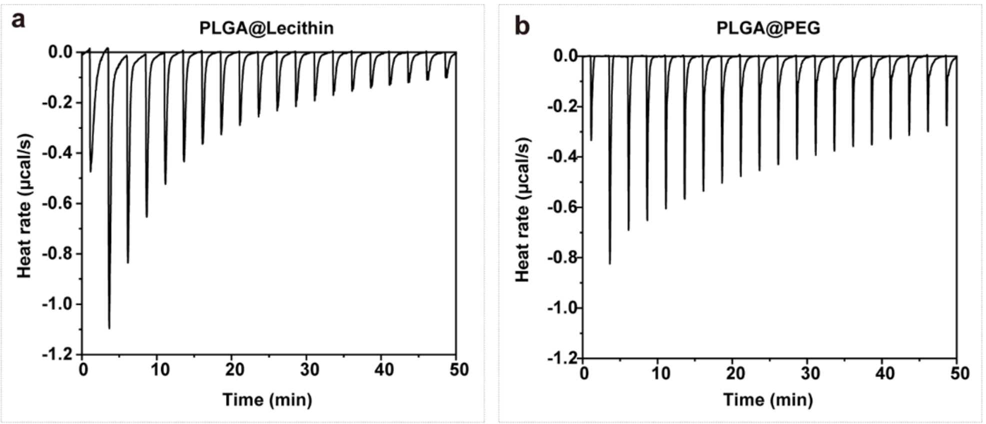

Supplementary Figure 4. Raw data from isothermal titration calorimetry (ITC) assays, in which HCS-supplemented water (30 mg/mL) was titrated into (a) PLGA@Lecithin nanoparticle and (b) PLGA@PEG nanoparticle dispersions (both at 20 mg/mL in water). Source data are provided as a Source Data file.

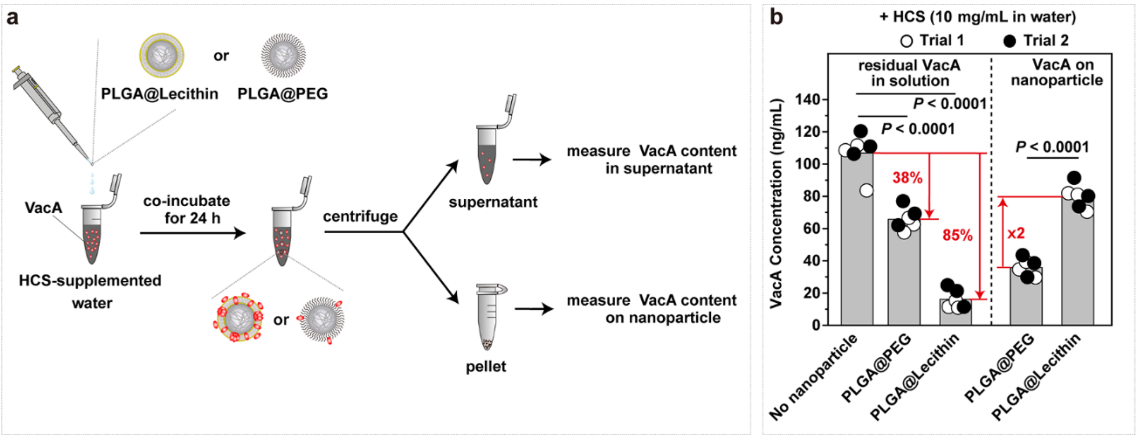

Supplementary Figure 5. VacA removal by nanoparticles. a. Schematic illustration of the assay for removing VacA from HCS-supplemented water with a nanoparticle (PLGA@Lecithin, or PLGA@PEG). **b.** Contents of adsorbed VacA on nanoparticles and those of residual VacA in the solution after 24 h incubation of the nanoparticles (2 mg/mL) in HCS-supplemented water (10 mg/mL). Bar heights are reported as averages of two independent trials (n = 3 in each independent trial). Statistical analysis was carried out with a one-way ANOVA with Tukey's multiple-

comparison test. Source data are provided as a Source Data file.

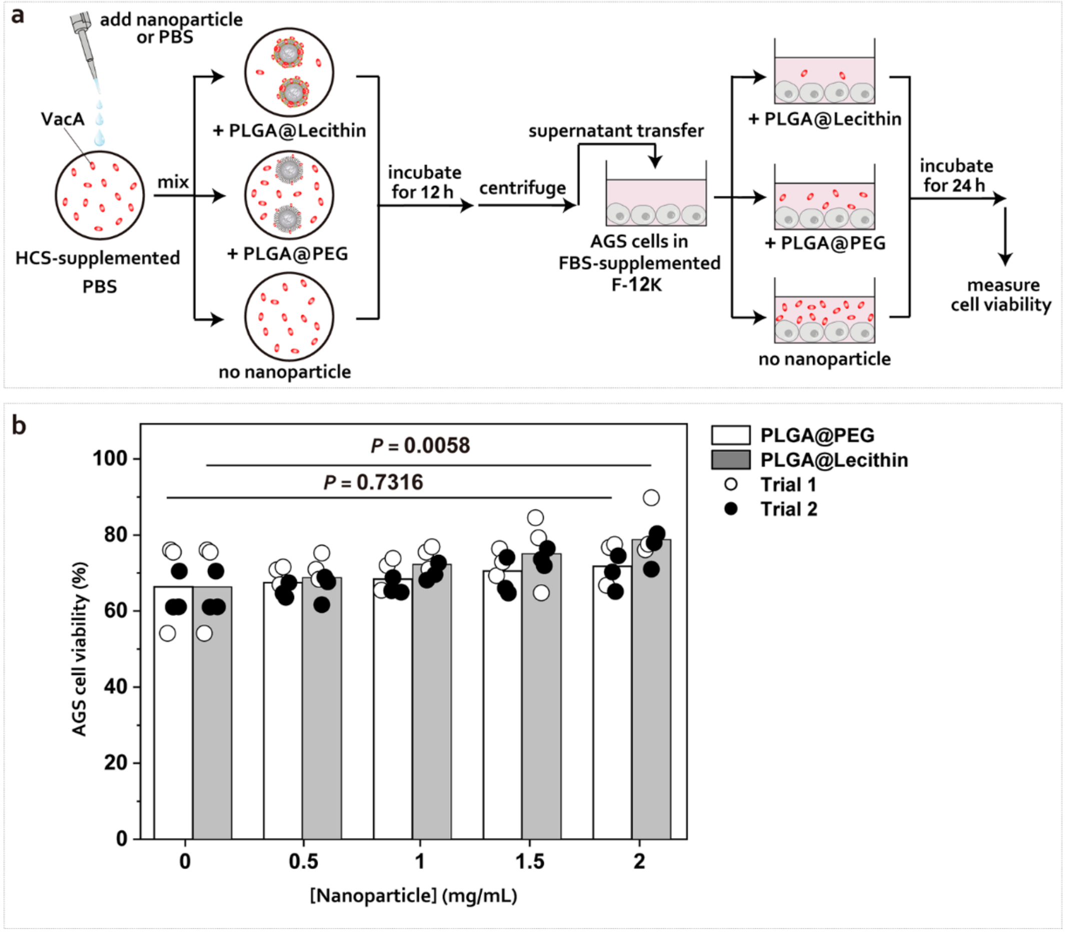

Supplementary Figure 6. Cytotoxicity of HCS after preincubation with nanoparticles. a. Schematic illustration of the cytotoxicity assay of HCS after 12 h of preincubation with or without a nanoparticle (PLGA@Lecithin, or PLGA@PEG). Human gastric adenocarcinoma cells (AGS cells) were used as representative gastric epithelial cells. **b.** Viability ratios of AGS cells after 24 h of treatment with HCS-supplemented PBS (10 mg/mL) preincubated with nanoparticles (PLGA@Lecithin, or PLGA@PEG) at different doses. Bar height represents the average of two independent trials (n = 3 in each independent trial). Statistical analysis was carried out with a two-

way ANOVA with Tukey’s multiple-comparison test. Source data are provided as a Source Data file.

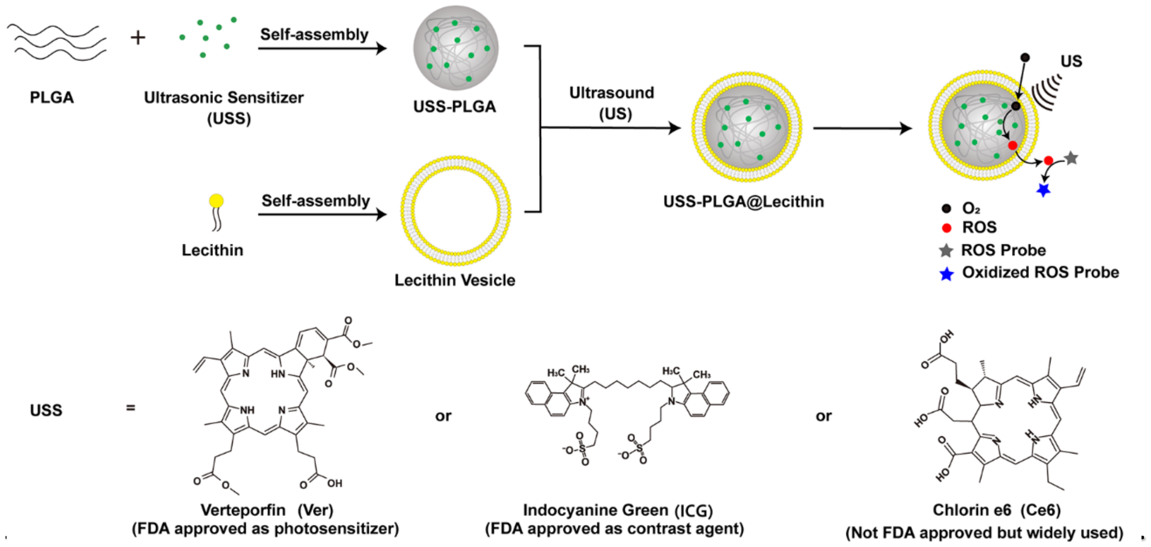

Supplementary Figure 7. Schematic illustration of (top) the preparation of a USS-PLGA@Lecithin nanoparticle, which can be Ver-PLGA@Lecithin, ICG-PLGA@Lecithin, or Ce6-PLGA@Lecithin depending on (bottom) the specific USS molecule preloaded, and ROS generation by the as-prepared USS-PLGA@Lecithin in response to US exposure.

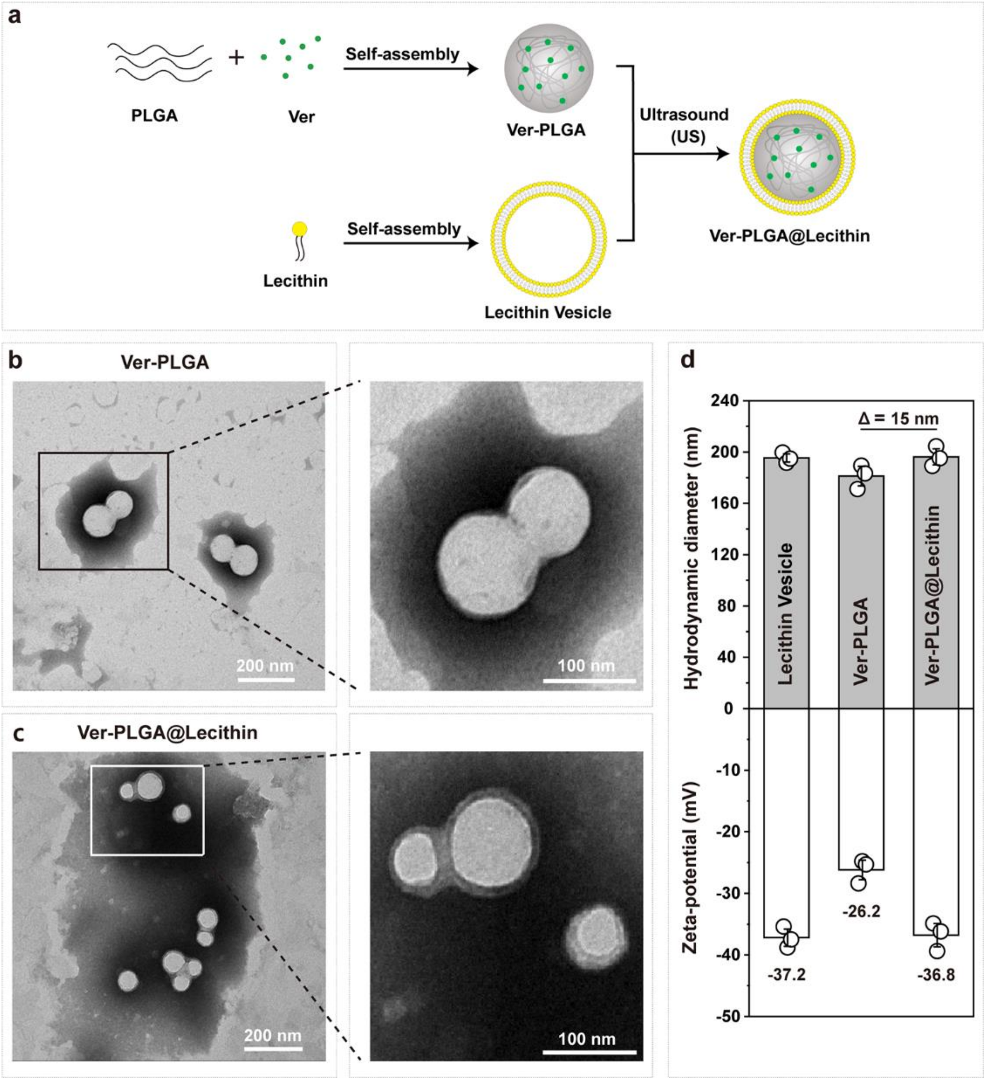

Supplementary Figure 8. Ver-PLGA@Lecithin nanoparticle preparation and characterization. a. Schematic illustration of the preparation of nanoparticles. **b-c.** TEM images of (b) Ver-PLGA nanoparticles and (c) Ver-PLGA@Lecithin nanoparticles. Two times, each experiment was repeated independently with similar results. **d.** The hydrodynamic diameters and zeta potentials of Ver-PLGA@Lecithin in Millipore water, with those of the precursor Ver-PLGA nanoparticle and lecithin vesicle included for comparison. Bar heights are reported as the average ± standard deviation (n = 3 in independent experiments). Source data are provided as a Source Data file.

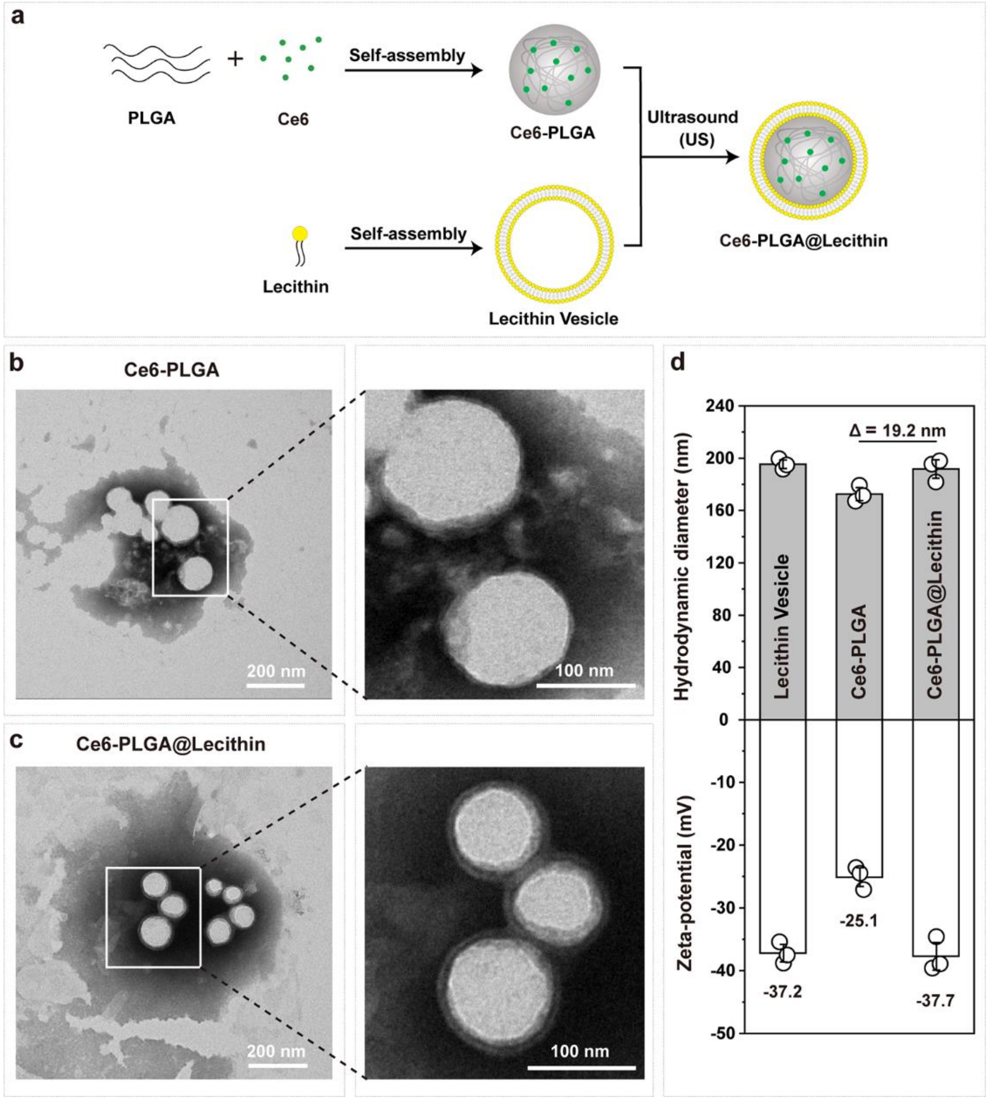

Supplementary Figure 9. Ce6-PLGA@Lecithin nanoparticle preparation and characterization. a. Schematic illustration of the preparation of Ce6-PLGA@Lecithin nanoparticles. **b-c.** TEM images of (b) Ce6-PLGA nanoparticles and (c) Ce6-PLGA@Lecithin nanoparticles. Two times, each experiment was repeated independently with similar results. **d.** The hydrodynamic diameters and zeta potentials of Ce6-PLGA@Lecithin in Millipore water, with those of the precursor Ce6-PLGA nanoparticle and lecithin vesicle included for comparison. Bar heights are reported as the average ± standard deviation (n = 3 in independent experiments). Source data are provided as a Source Data file.

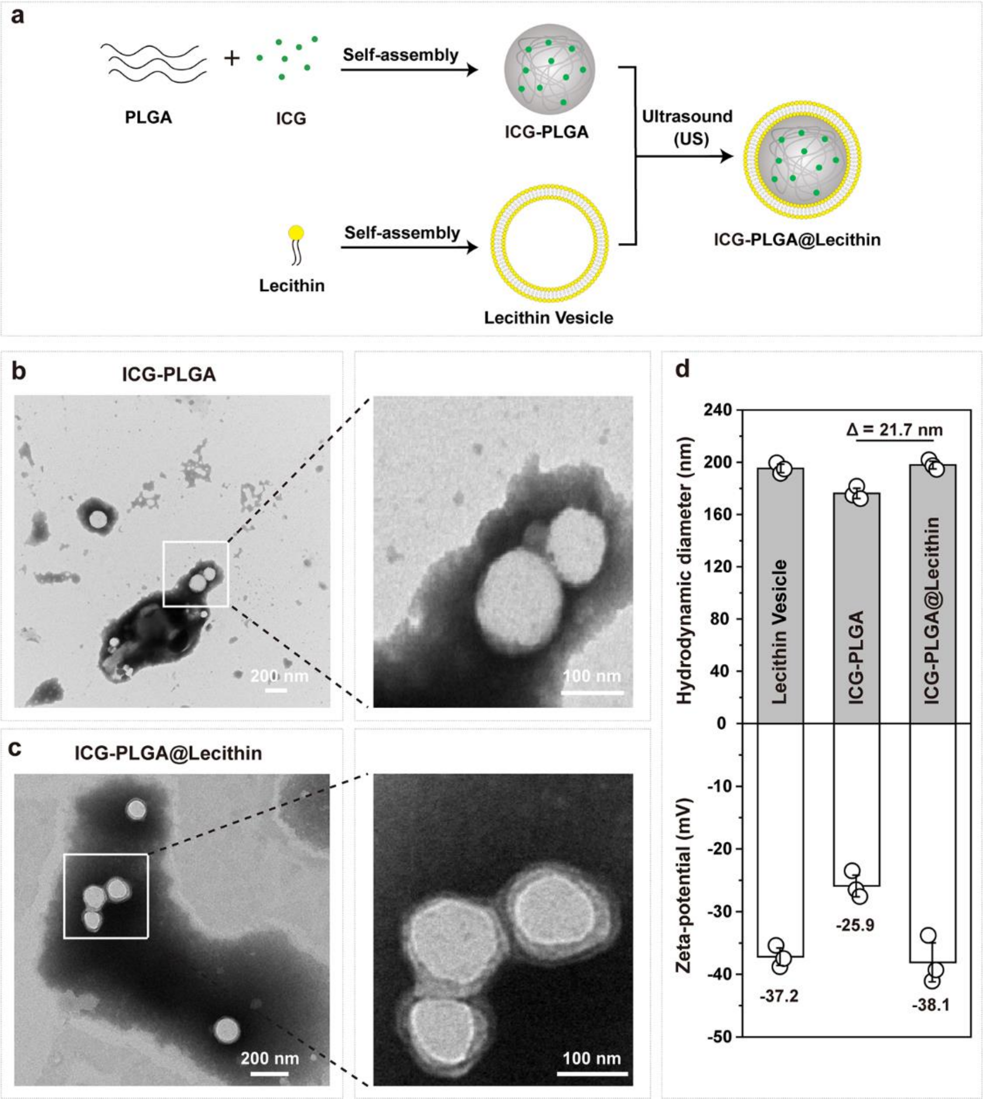

Supplementary Figure 10. ICG-PLGA@Lecithin nanoparticle preparation and characterization. a. Schematic illustration of the preparation of ICG-PLGA@Lecithin nanoparticles. **b-c.** TEM images of (b) ICG-PLGA nanoparticles and (c) ICG-PLGA@Lecithin nanoparticles. Two times, each experiment was repeated independently with similar results. **d.** The hydrodynamic diameters and zeta potentials of ICG-PLGA@Lecithin in Millipore water, with those of the precursor ICG-PLGA nanoparticle and lecithin vesicle included for comparison. Bar heights are reported as the average ± standard deviation (n = 3 in independent experiments). Source data are provided as a Source Data file.

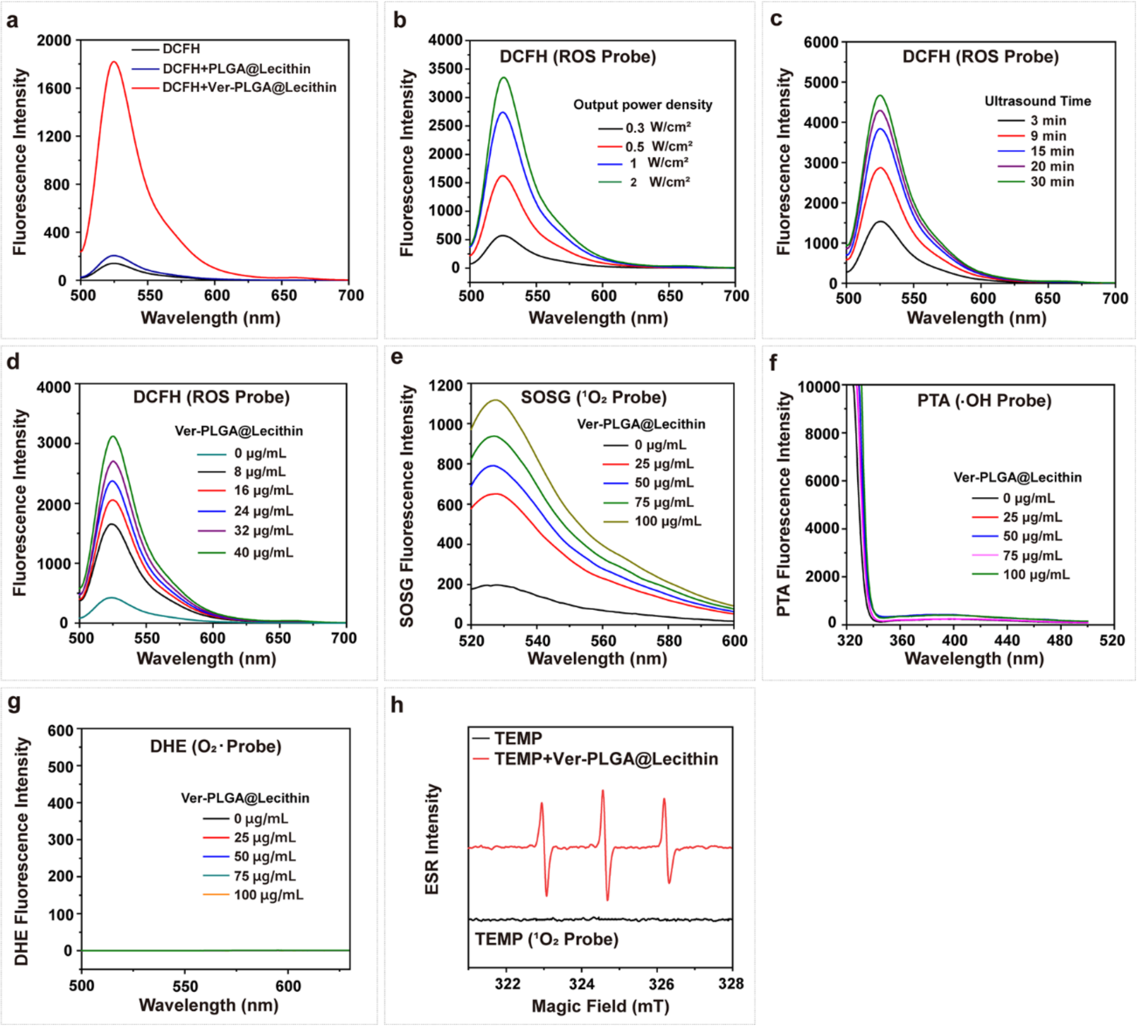

Supplementary Figure 11. ROS generation by Ver-PLGA@Lecithin nanoparticles. a. Fluorescence emission spectra of 2',7'-dichlorodihydrofluorescein (DCFH) (100 µL, 40 µM), PLGA@Lecithin nanoparticles (100 µL, 100 µg/mL) and Ver-PLGA@Lecithin nanoparticles (100 µL, 100 µg/mL) under US conditions (0.5 W/cm^2, 10 min). **b.** Fluorescence emission spectra of Ver-PLGA@Lecithin nanoparticles (100 µL, 100 µg/mL) with DCFH (100 µL, 40 µM) as an ROS probe at different US intensities (fixed US time of 10 min). **c.** Fluorescence emission spectra of Ver-PLGA@Lecithin nanoparticles (100 µL, 100 µg/mL) with DCFH (100 µL, 40 µM) as an ROS probe at different US times (fixed US intensity of 0.5 W/cm^2). **d.** Fluorescence emission spectra of Ver-PLGA@Lecithin nanoparticles (100 µL) at different concentrations with DCFH (100 µL,

40 μM) as an ROS probe under US conditions (0.5 W/cm^2, 10 min). **e.** Fluorescence emission spectra of Ver-PLGA@Lecithin nanoparticles (100 μL) at different concentrations under US conditions (0.5 W/cm^2, 10 min). The generation of $^1O_2$ under US conditions was monitored by singlet oxygen sensor green (SOSG) as a selective fluorescent probe. **f.** Fluorescence emission spectra of Ver-PLGA@Lecithin nanoparticles (100 μL) at different concentrations under US conditions (0.5 W/cm^2, 10 min). The generation of •OH under US conditions was monitored by purified terephthalic acid (PTA) as a selective fluorescent probe. **g.** Fluorescence emission spectra of Ver-PLGA@Lecithin nanoparticles (100 μL) at different concentrations under US conditions (0.5 W/cm^2, 10 min). The generation of $O_2^{-\bullet}$ under US conditions was monitored by dihydroethidium (DHE) as a selective fluorescent probe. **h.** Electron spin resonance (ESR) spectra of Ver-PLGA@Lecithin nanoparticles (50 μg/mL) with 2,2,6,6-tetramethylpiperidin (TEMP) as a $^1O_2$ probe under US conditions (0.5 W/cm^2, 10 min). Source data are provided as a Source Data file.

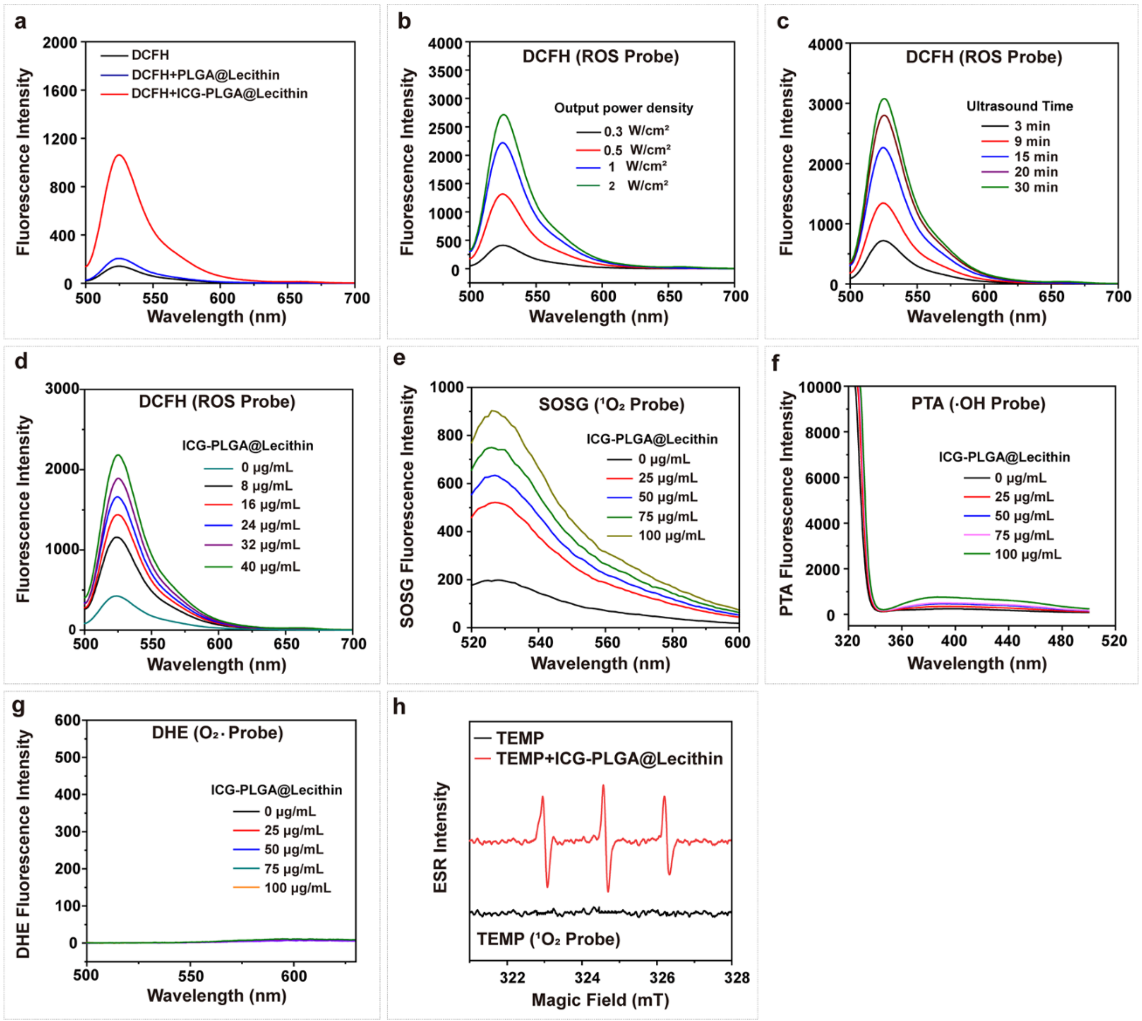

Supplementary Figure 12. ROS generation by ICG-PLGA@Lecithin nanoparticles. a. Fluorescence emission spectra of DCFH (100 μL, 40 μM), PLGA@Lecithin nanoparticles (100 μL) and ICG-PLGA@Lecithin nanoparticles (100 μL, 100 μg/mL) under US conditions (0.5 W/cm^2, 10 min). **b.** Fluorescence emission spectra of ICG-PLGA@Lecithin nanoparticles (100 μL, 100 μg/mL) with DCFH (100 μL, 40 μM) as an ROS probe at different US intensities (fixed US time of 10 min). **c.** Fluorescence emission spectra of ICG-PLGA@Lecithin nanoparticles (100 μL, 100 μg/mL) with DCFH (100 μL, 40 μM) as an ROS probe at different US times (fixed US intensity of 0.5 W/cm^2). **d.** Fluorescence emission spectra of ICG-PLGA@Lecithin nanoparticles (100 μL) at different concentrations with DCFH (100 μL, 40 μM) as an ROS probe under US

conditions (0.5 W/cm^2, 10 min). **e.** Fluorescence emission spectra of ICG-PLGA@Lecithin nanoparticles (100 μL) at different concentrations under US conditions (0.5 W/cm^2, 10 min). The generation of $^1O_2$ under US conditions was also monitored by SOSG as a selective fluorescent probe. **f.** Fluorescence emission spectra of ICG-PLGA@Lecithin nanoparticles (100 μL, 100 μg/mL) at different concentrations under US conditions (0.5 W/cm^2, 10 min). The generation of •OH under US conditions was monitored by PTA as a selective fluorescent probe. **g.** Fluorescence emission spectra of ICG-PLGA@Lecithin nanoparticles (100 μL, 100 μg/mL) at different concentrations under US conditions (0.5 W/cm^2, 10 min). The generation of $O_2^{-\bullet}$ under US conditions was monitored by DHE as a selective fluorescent probe. **h.** ESR spectra of ICG-PLGA@Lecithin nanoparticles (50 μg/mL) with TEMP as a $^1O_2$ probe under US conditions (0.5 W/cm^2, 10 min). Source data are provided as a Source Data file.

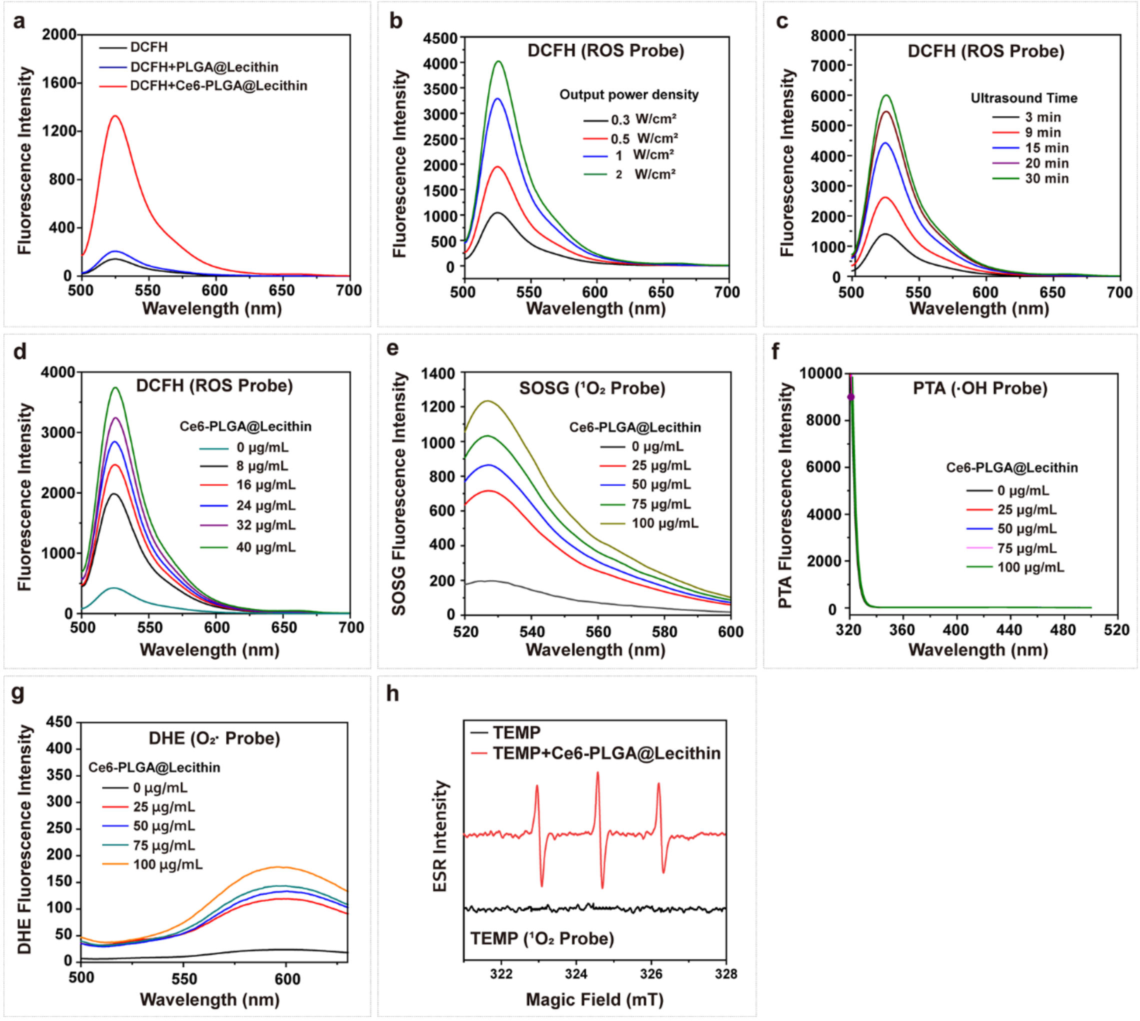

Supplementary Figure 13. ROS generation by Ce6-PLGA@Lecithin nanoparticles. a. Fluorescence emission spectra of DCFH (100 μL, 40 μM), PLGA@Lecithin nanoparticles (100 μL) and Ce6-PLGA@Lecithin nanoparticles (100 μL, 100 μg/mL) under US conditions (0.5 W/cm^2, 10 min). **b.** Fluorescence emission spectra of Ce6-PLGA@Lecithin nanoparticles (100 μL, 100 μg/mL) with DCFH (100 μL, 40 μM) as an ROS probe at different US intensities (fixed US time of 10 min). **c.** Fluorescence emission spectra of Ce6-PLGA@Lecithin nanoparticles (100 μL, 100 μg/mL) with DCFH (100 μL, 40 μM) as an ROS probe at different US times (fixed US intensity of 0.5 W/cm^2). **d.** Fluorescence emission spectra of Ce6-PLGA@Lecithin nanoparticles (100 μL) at different concentrations with DCFH (100 μL, 40 μM) as an ROS probe under US

conditions (0.5 W/cm^2, 10 min). **e.** Fluorescence emission spectra of Ce6-PLGA@Lecithin nanoparticles (100 μL) at different concentrations under US conditions (0.5 W/cm^2, 10 min). The generation of $^1O_2$ under US conditions was monitored by SOSG as a selective fluorescent probe. **f.** Fluorescence emission spectra of Ce6-PLGA@Lecithin nanoparticles (100 μL) at different concentrations under US conditions (0.5 W/cm^2, 10 min). The generation of •OH under US conditions was monitored by PTA as a selective fluorescent probe. **g.** Fluorescence emission spectra of Ce6-PLGA@Lecithin nanoparticles (100 μL) at different concentrations under US conditions (0.5 W/cm^2, 10 min). The generation of $O_2^{-\bullet}$ under US conditions was monitored by DHE as a selective fluorescent probe. **h.** ESR spectra of Ce6-PLGA@Lecithin nanoparticles (50 μg/mL) with TEMP as a $^1O_2$ probe under US conditions (0.5 W/cm^2, 10 min). Source data are provided as a Source Data file.

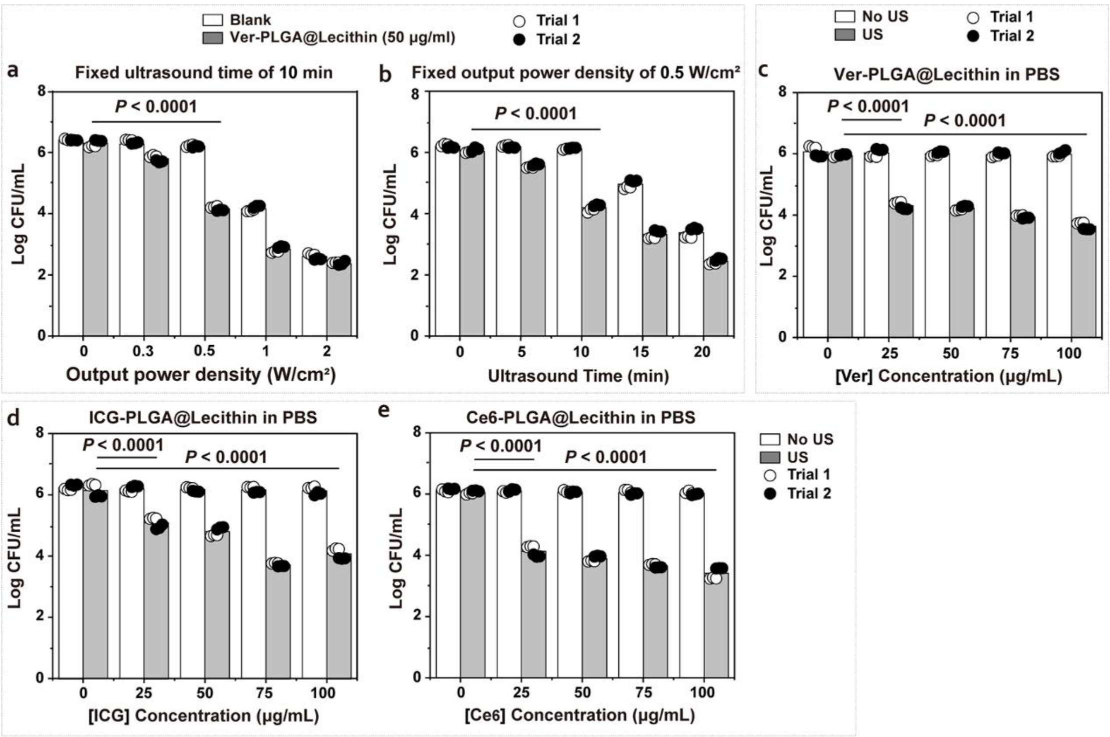

Supplementary Figure 14. *In vitro* antibacterial assays of USS-PLGA@Lecithin nanoparticles. a-b. *In vitro* antibacterial properties of Ver-PLGA@Lecithin nanoparticles under different conditions. **c-e.** *In vitro* antibacterial properties of USS-PLGA@Lecithin nanoparticles (in PBS) at different concentrations under US conditions (0.5 W/cm^2, 10 min). Bar heights are reported as the average of the two independent experiments (n = 3 in each independent trial). Statistical analysis was carried out with a two-way ANOVA with Tukey's multiple-comparison test. Source data are provided as a Source Data file.

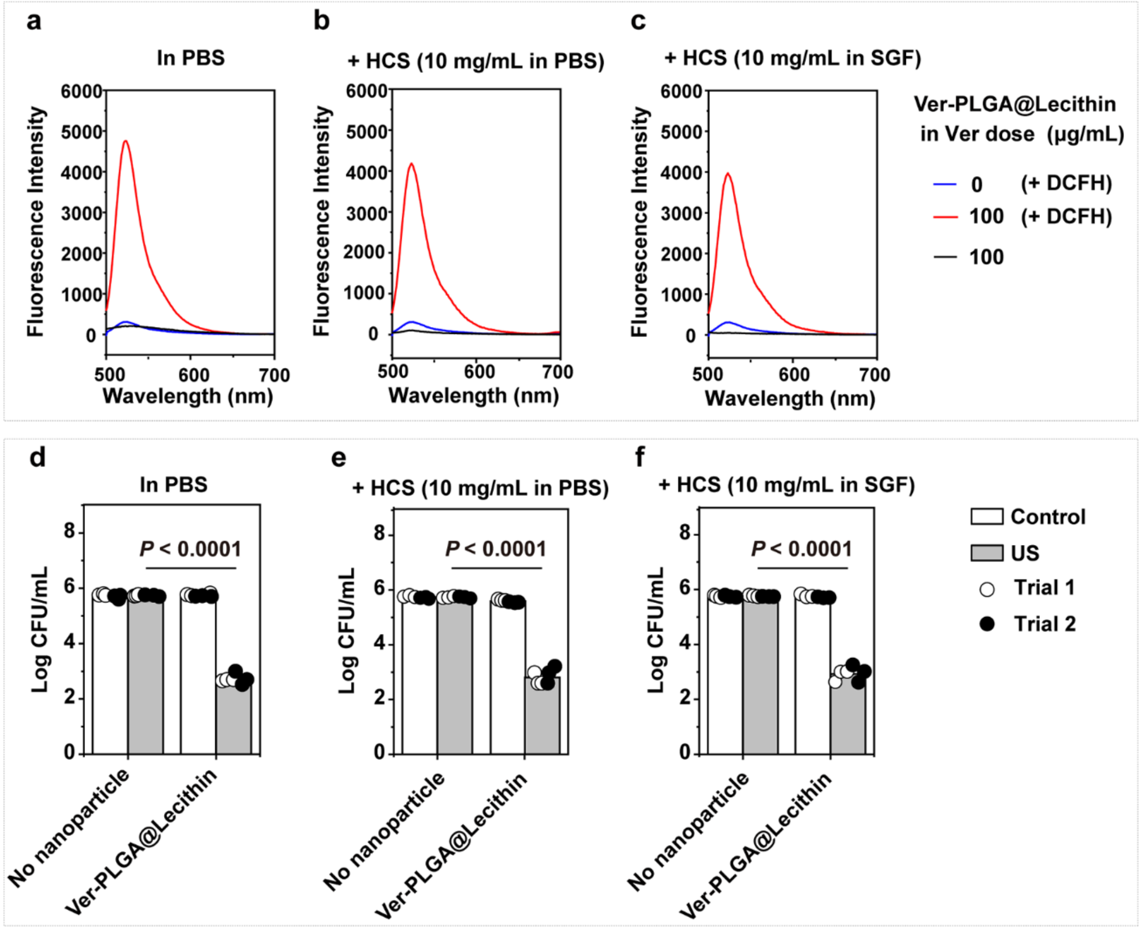

Supplementary Figure 15. Effects of protein adsorption on Ver-PLGA@Lecithin's performance *in vitro*. a-c. Fluorescence emission spectra of DCFH (100 µL, 40 µM) in the presence of US exposure (0.5 W/cm^2, 10 min) and Ver-PLGA@Lecithin (100 µL, 0 or 100 µg/mL) were recollected after 12 h of preincubation in (a) PBS, (b) HCS-supplemented PBS (10 mg/mL), or (c) HCS-supplemented SGF (10 mg/mL). Fluorescence emission spectra of samples treated similarly but without DCFH (100 µL, 40 µM) were included to exclude the possibility of self-fluorescence from the nanoparticle and/or protein-present media. **d-f.** Counts of viable *H. pylori* cells (in Log CFU/mL) after treatment with Ver-PLGA@Lecithin (100 µL, 0 or 100 µg/mL) recollected after 12-h preincubation in (d) PBS, (e) HCS-supplemented PBS (10 mg/mL), or (f) HCS-supplemented SGF (10 mg/mL) under US exposure (0.5 W/cm^2, 10 min). Controls are

samples treated similarly but without US exposure. Bar height represents the average of two independent trials (n = 3 in each independent trial). Statistical analysis was carried out with a two-way ANOVA with Tukey's multiple-comparison test. Source data are provided as a Source Data file.

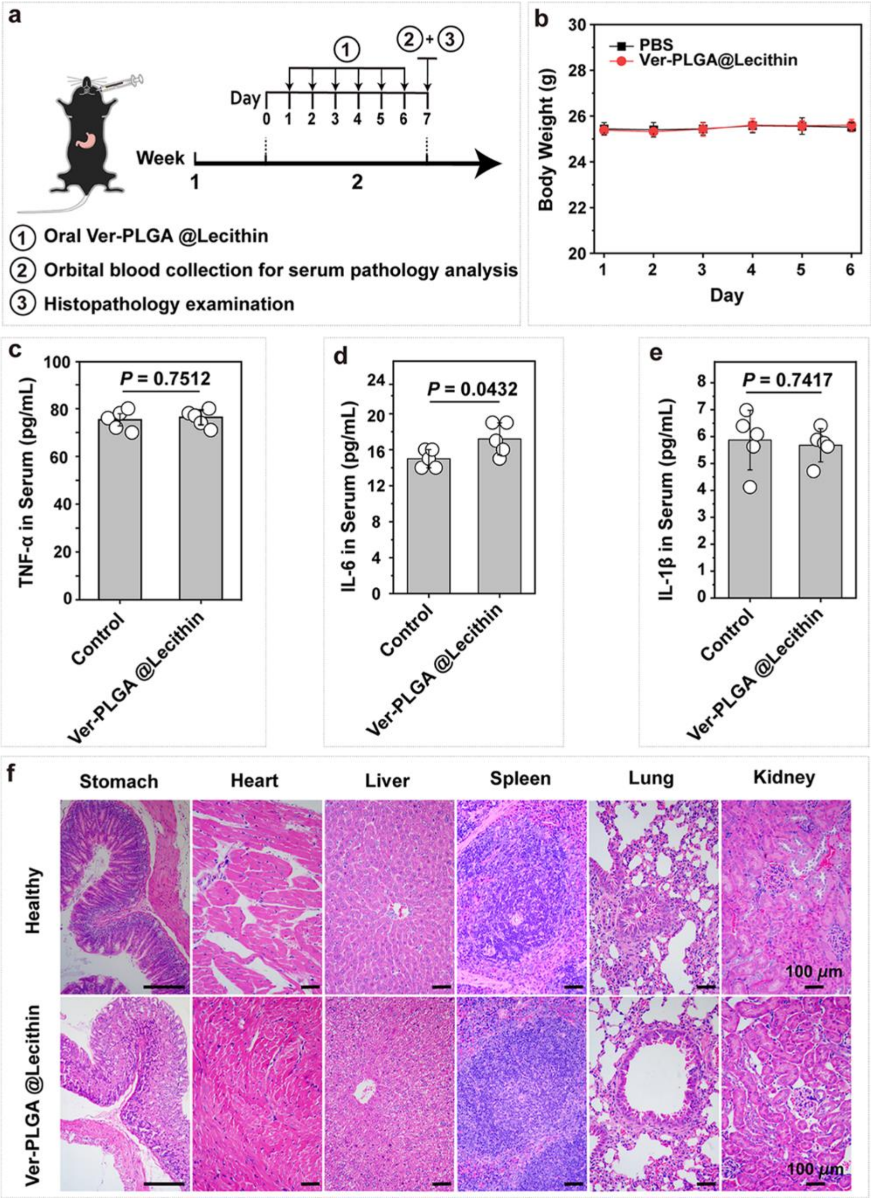

Supplementary Figure 16. Intrinsic biosafety of Ver-PLGA@Lecithin. a. Schematic illustration of the schedule for evaluating the biosafety of orally administered Ver-PLGA@Lecithin in healthy C57BL/6J mice. **b.** Mouse body weights over 6 days after oral administration of Ver-PLGA@Lecithin. **c-e.** Serum levels of (c) TNF-α (d) IL-6 and (e) IL-1β at 24 h after treatment completion. Control indicates treatment with PBS alone (*i.e.*, healthy). Bar heights are reported as the average ± standard deviation (n = 5 biologically independent mice in

one experiment). *P* values were calculated using two-sided Student's t test. **f.** Microscopy images of hematoxylin and eosin (H&E)-stained stomach tissues and organs collected from mice in the healthy group and the treatment group. Mice in the treatment group were sacrificed at 24 h after treatment completion. Scale bar = 100 μm. Source data are provided as a Source Data file.

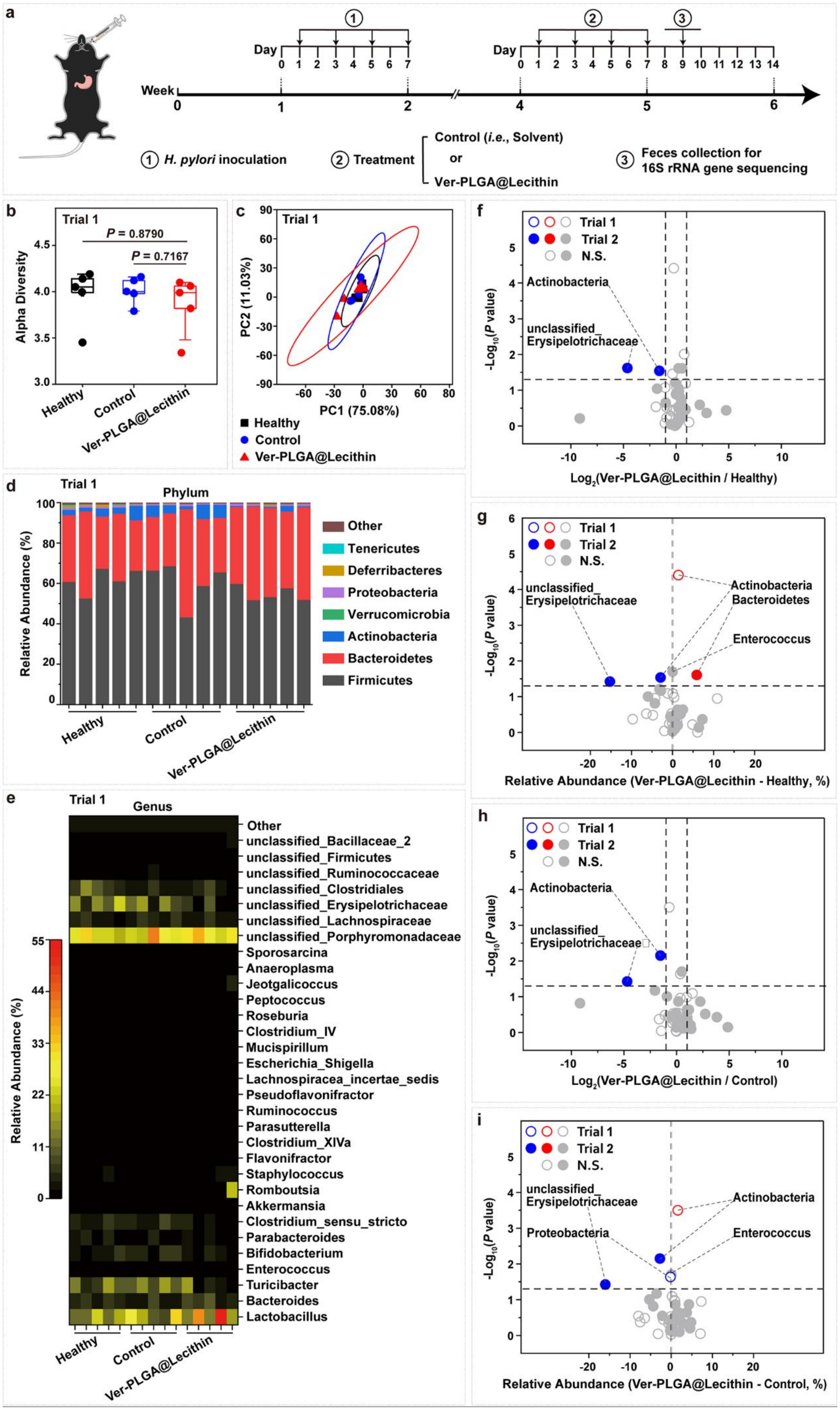
a
Day
0 1 2 3 4 5 6 7
0 1 2 3 4 5 6 7 8 9 10 11 12 13 14
Week
0 1 2 4 5 6
① H. pylori inoculation
② Treatment
Control (i.e., Solvent)
or
Ver-PLGA@Lecithin
③ Feces collection for
16S rRNA gene sequencing
b
Trial 1
P = 0.8790
P = 0.7167
Alpha Diversity
Healthy
Control
Ver-PLGA@Lecithin
c
Trial 1
PC2 (11.03%)
PC1 (75.08%)
Healthy
Control
Ver-PLGA@Lecithin
d
Trial 1
Phylum
Relative Abundance (%)
Other
Tenericutes
Deferribacteres
Proteobacteria
Verrucomicrobia
Actinobacteria
Bacteroidetes
Firmicutes
Healthy
Control
Ver-PLGA@Lecithin
e
Trial 1
Genus
Relative Abundance (%)
Other
unclassified_Bacillaceae_2
unclassified_Firmicutes
unclassified_Ruminococcaceae
unclassified_Clostridiales
unclassified_Erysipelotrichaceae
unclassified_Lachnospiraceae
unclassified_Porphyromonadaceae
Sporosarcina
Anaeroplasma
Jeotgalicoccus
Peptococcus
Roseburia
Clostridium_IV
Mucispirillum
Escherichia_Shigella
Lachnospiracea_incertae_sedis
Pseudoflavonifractor
Ruminococcus
Parasutterella
Clostridium_XIVa
Flavonifractor
Staphylococcus
Romboutsia
Akkermansia
Clostridium_sensu_stricto
Parabacteroides
Bifidobacterium
Enterococcus
Turicibacter
Bacteroides
Lactobacillus
Healthy
Control
Ver-PLGA@Lecithin
f
Trial 1
Trial 2
N.S.
Actinobacteria
unclassified_
Erysipelotrichaceae
-Log10(P value)
Log2(Ver-PLGA@Lecithin / Healthy)
g
Trial 1
Trial 2
N.S.
unclassified_
Erysipelotrichaceae
Actinobacteria
Bacteroidetes
Enterococcus
-Log10(P value)
Relative Abundance (Ver-PLGA@Lecithin - Healthy, %)
h
Trial 1
Trial 2
N.S.
Actinobacteria
unclassified_
Erysipelotrichaceae
-Log10(P value)
Log2(Ver-PLGA@Lecithin / Control)
i
Trial 1
Trial 2
N.S.
unclassified_
Erysipelotrichaceae
Proteobacteria
Actinobacteria
Enterococcus
-Log10(P value)
Relative Abundance (Ver-PLGA@Lecithin - Control, %)

Supplementary Figure 17. Gut microbiota after oral administration of Ver-PLGA@Lecithin nanoparticles. a. Schematic illustration of the schedule of oral administration of Ver-PLGA@Lecithin nanoparticle treatment in mouse models bearing gastric *H. pylori* infection and follow-up feces collection for (b-e) gut microbiota analysis, with healthy counterparts included as references. **b-c.** (b) α diversity and (c) principal component analysis (PCA) of the beta (β) diversity of the gut microbiota of mice in the healthy group, the control group and the oral administration of Ver-PLGA@Lecithin group. The boxes denote the lower 25% quantile, upper 75% quantile, and centerline the median, with whiskers extending to a limit of ±1.5 interquartile ranges (IQRs). (n = 5 biologically independent mice in one trial). Statistical analysis was carried out with a one-way ANOVA with Tukey's multiple-comparison test. **d-e.** The relative abundances of gut bacterial species at the (d) phylum and (e) genus levels in mouse models after differing treatments, with those of the healthy group included for comparison. The data were clustered according to the number of samples per group on the abscissa. **f-i.** Plots of 16S rRNA gene sequencing results on (f-g) the difference between the oral administration of Ver-PLGA@Lecithin group *versus* healthy group and (h-i**)** on the difference between the oral administration of Ver-PLGA@Lecithin nanoparticle groups *versus* control groups. The change in the relative abundance of gut bacterial species after oral administration of Ver-PLGA@Lecithin nanoparticles is indicated both as (f, h) the fold change (*i.e.*, $\log_2$(Ver-PLGA@Lecithin/Healthy), and $\log_2$(Ver-PLGA@Lecithin/Control)) and (g, i) as the absolute value of change (*i.e.*, Relative Abundance (Ver-PLGA@Lecithin-Healthy, %), and Relative Abundance (Ver-PLGA@Lecithin-Control, %)). For a bacterium to be marked as a significantly perturbed bacterium in the plots of $-\log_{10}$(*P* value) *versus* the fold change in bacterial relative abundance, its fold change in bacterial relative abundance needs to be ≥2 or ≤1/2, and its *P* value needs to be <0.05. For a bacterium to be marked as a significantly perturbed

bacterium in the plots of $-\log_{10}$(P value) *versus* the absolute change in bacterial relative abundance, its P value needs to be <0.05. Bacterial species whose relative abundances were significantly up- and downregulated after triple therapy are marked in red and blue, respectively. Statistical analysis was carried out with a two-sided Student's t test. Source data are provided as a Source Data file.

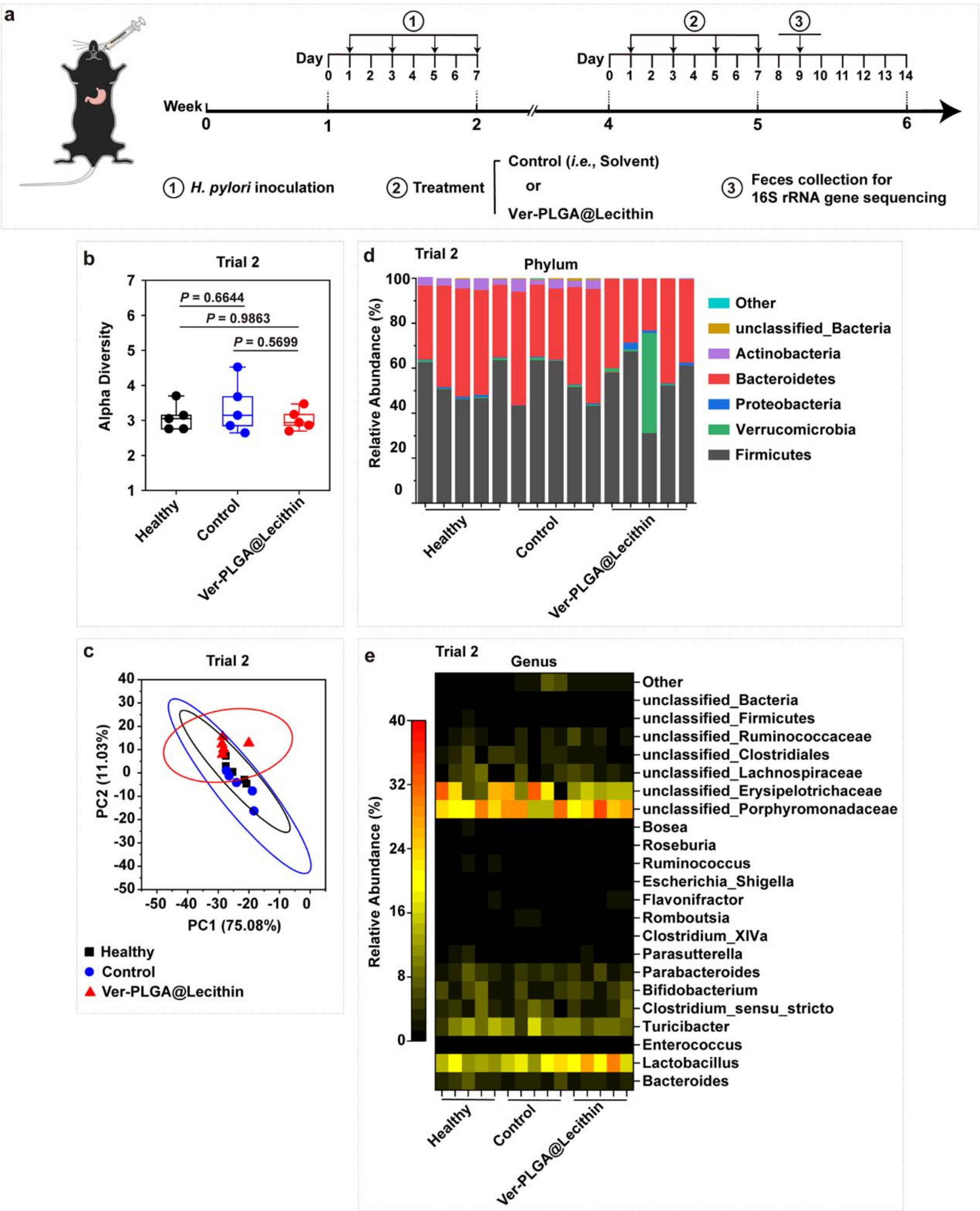

Supplementary Figure 18. Effects of oral administration of Ver-PLGA@Lecithin nanoparticles on the gut microbiota. a. Schematic illustration of the schedule of oral administration of Ver-PLGA@Lecithin nanoparticle treatment in mouse models bearing gastric *H. pylori* infection and follow-up feces collection for (b-e) gut microbiota analysis, with healthy

counterparts included as references. **b-c.** The (b) alpha (α) diversity (Shannon) and (c) principal component analysis (PCA) on the beta (β) diversity of gut microbiota of mice in the healthy group, the control group and the oral administration of Ver-PLGA@Lecithin nanoparticle group. The boxes denote the lower 25% quantile, upper 75% quantile, and centerline the median, with whiskers extending to a limit of ±1.5 interquartile ranges (IQRs). (n = 5 biologically independent mice in one trial). Statistical analysis was carried out with a one-way ANOVA with Tukey's multiple-comparison test. **d-e.** The relative abundances of gut bacterial species at the (d) phylum and (e) genus levels in mouse models after differing treatments, with those of the healthy group included for comparison. The data were clustered according to the number of samples per group on the abscissa. Source data are provided as a Source Data file.

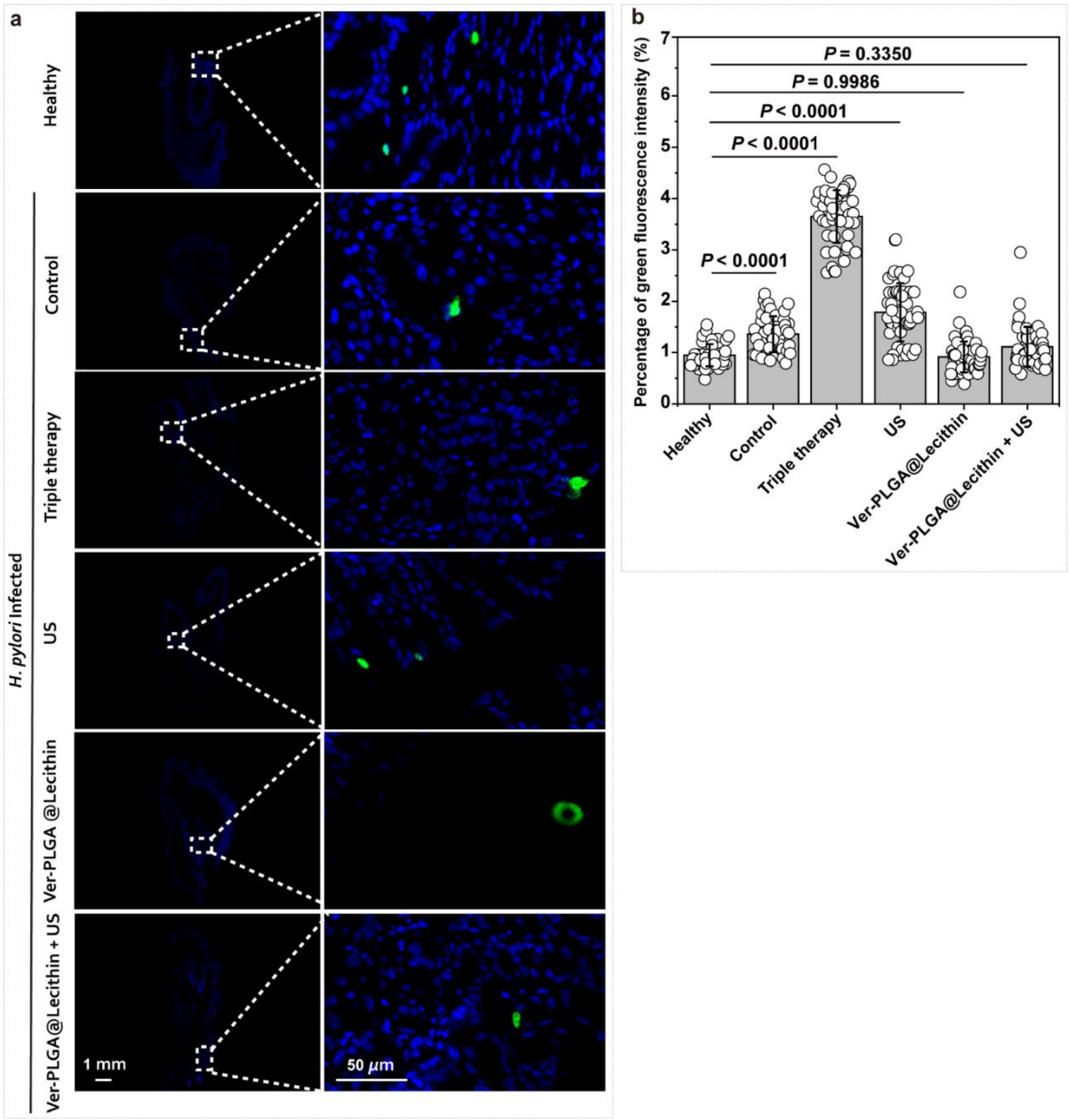

Supplementary Figure 19. Analysis of TUNEL-stained stomach tissues. a. Representative fluorescence microscopy images of TUNEL (TdT-mediated dUTP nick-end labelling)-stained stomach tissues collected from gastric *H. pylori* infection-bearing mouse models at 48 h after differing treatments (*i.e.*, control, triple therapy, US exposure alone, Ver-PLGA@Lecithin alone, and (Ver-PLGA@Lecithin + US)), with those from their healthy counterparts included as references. Blue fluorescence (by DAPI) indicates DNA, and green fluorescence (by FITC- dUTP) indicates apoptotic cell death. **b.** Percentages of green fluorescence intensity in (a) the TUNEL-

stained images, which were obtained by imaging 50 different fields of view for each treatment group. Bar heights are reported as the average ± standard deviation. Statistical analysis was carried out with a one-way ANOVA with Tukey's multiple-comparison test. Source data are provided as a Source Data file.

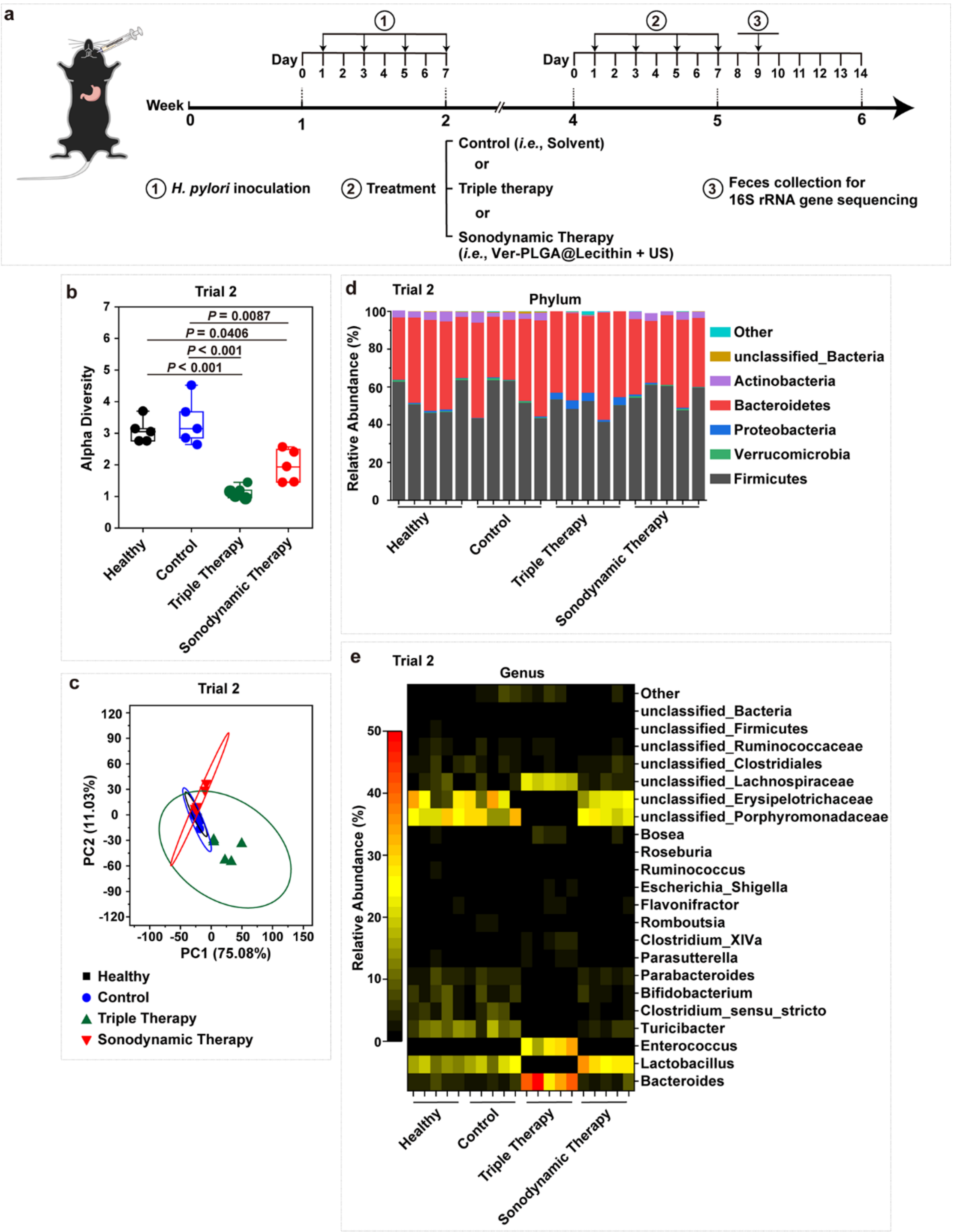

Supplementary Figure 20. Gut microbiota after sonodynamic therapy. a. Schematic illustration on the schedule of differing treatment to mouse models bearing gastric *H. pylori* infection and follow-up feces collection for (b-e) gut microbiota analysis, with healthy

counterparts included as references. **b-c.** The (b) α diversity and (c) β diversity of gut microbiota by principal component analysis (PCA) in mouse models after differing treatment, with those of the healthy group included for comparison. The boxes denote the lower 25% quantile, upper 75% quantile, and centerline the median, with whiskers extending to a limit of ±1.5 interquartile ranges (IQRs). (n = 5 biologically independent mice in one trial). Statistical analysis was carried out with a one-way ANOVA with Tukey's multiple-comparison test. **d-e.** The relative abundances of gut bacterial species at the (d) phylum and (e) genus levels in mouse models after differing treatments, with those of the healthy group included for comparison. The data were clustered according to the number of samples per group on the abscissa. Source data are provided as a Source Data file.

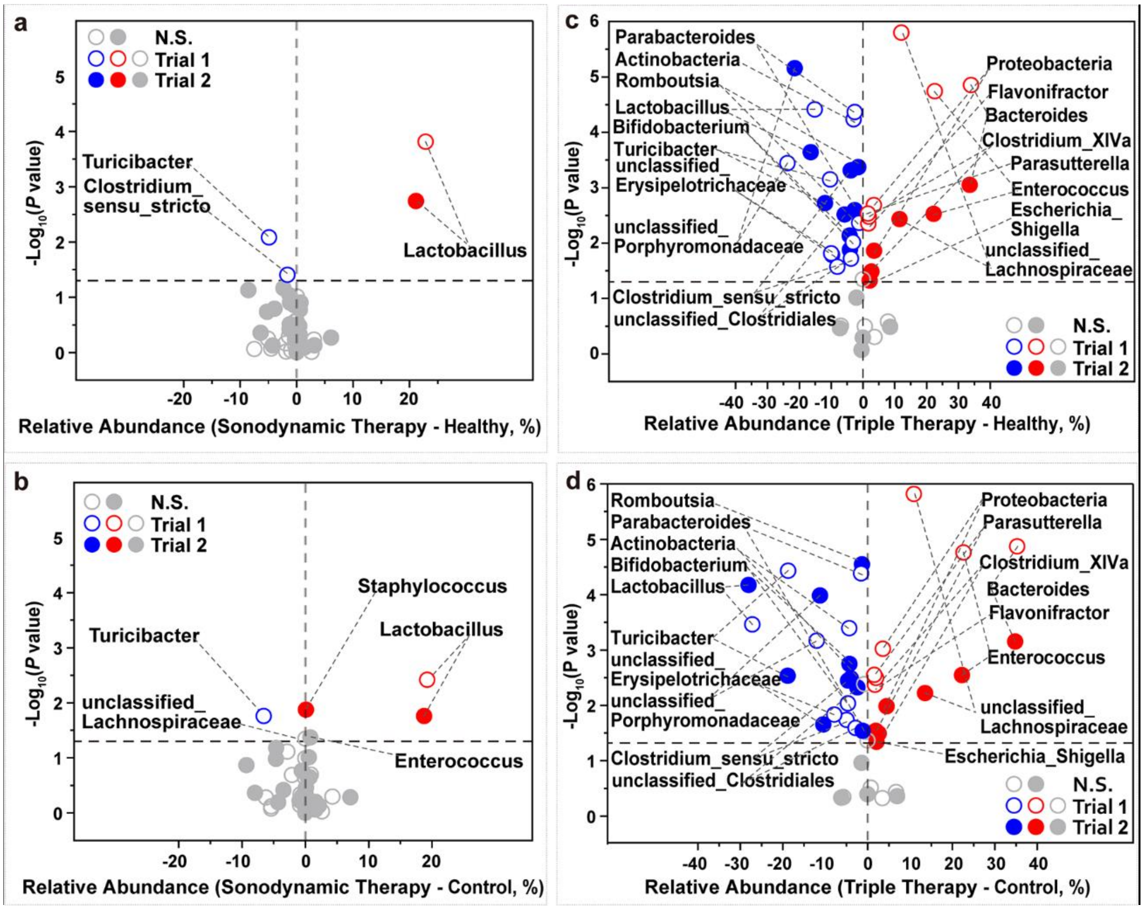

Supplementary Figure 21. Gut microbiota after triple therapy *versus* sonodynamic therapy. Statistical analysis of how (a, b) sonodynamic therapy and (c, d) triple therapy affect the composition of gut commensal bacteria, with (a, c) the healthy group and (b, d) the control group used as references. The change in the relative abundance of gut bacterial species after sonodynamic therapy is indicated as the absolute value of change (a) compared to the healthy group (*i.e.*, Relative Abundance (Sonodynamic therapy-Healthy, %)) and (c) compared to the control group (*i.e.*, Relative Abundance (Sonodynamic therapy-Control, %)). Similarly, the change in relative abundance of gut bacterial species after triple therapy is indicated both as (c) the absolute value of change compared to the healthy group (*i.e.*, Relative Abundance (Triple therapy-Healthy, %)) and (d) that compared to the control group (*i.e.*, Relative Abundance (Triple therapy-Control, %)). For a bacterium to be marked as a significantly perturbed bacterium, its P value needs to be <0.05.

Bacterial species whose relative abundances were significantly up- and downregulated after sonodynamic therapy are marked in red and blue, respectively. Statistical analysis was carried out with a two-sided Student's t test. Source data are provided as a Source Data file.

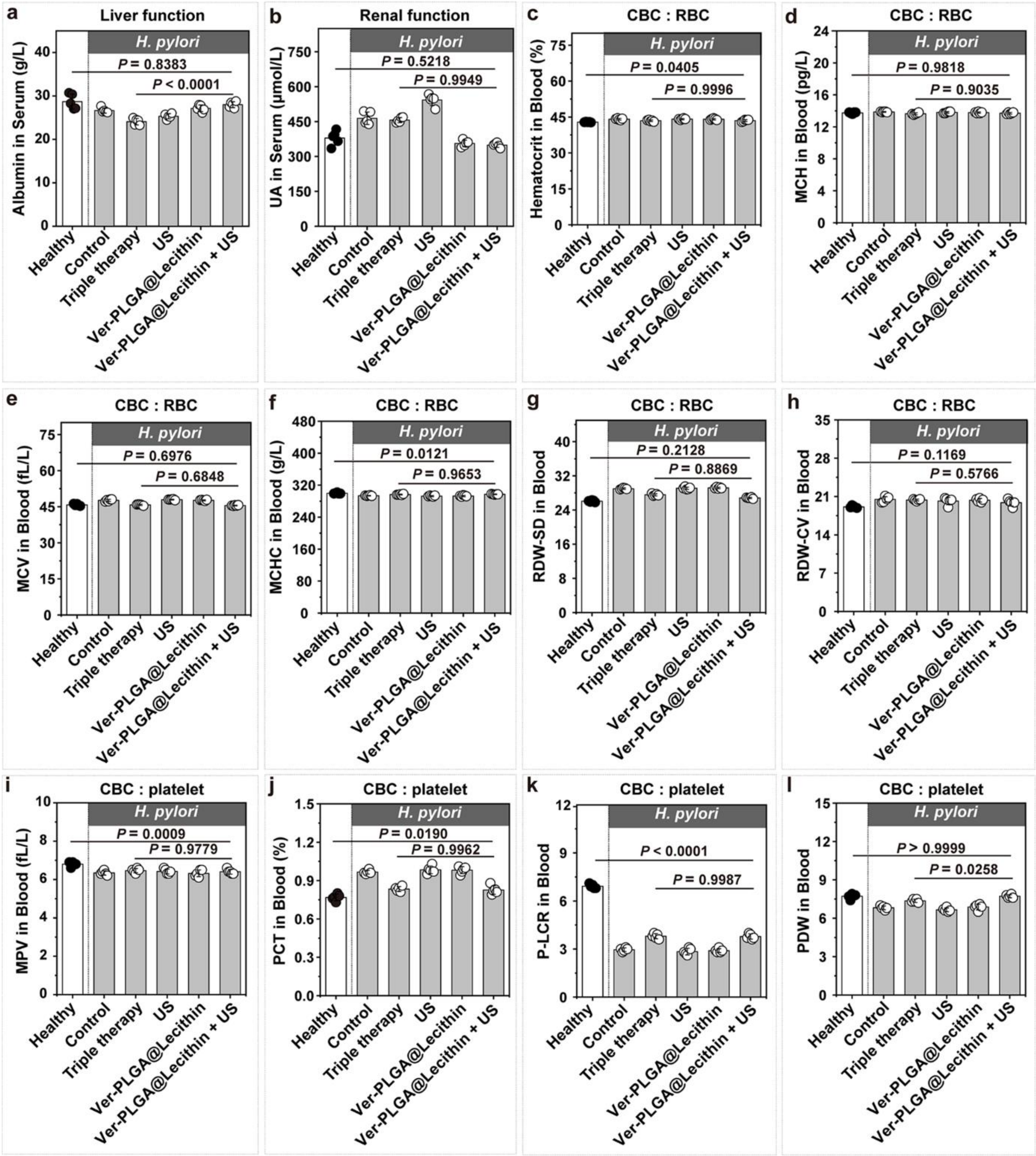

Supplementary Figure 22. Biosafety of the sonodynamic therapy mediated by Ver-PLGA@Lecithin. The extended data for Fig. 7, which show serum levels of (a) albumin and (b) uric acid (UA) in the liver and renal function tests and blood levels of (c) hematocrit, (d) mean corpuscular hemoglobin (MCH), (e) mean corpuscular volume (MCV), (f) mean corpuscular hemoglobin concentration (MCHC), (g) red blood cell distribution width-standard deviation

(RDW-SD) and (h) red blood cell volume distribution width-coefficient of variation (RDW-CV), (i) mean platelet volume (MPV), (j) plateletcrit (PCT) %, (k) platelet-larger cell ratio (P-LCR). and (l) platelet distribution width (PDW) in the CBC test. All blood samples were collected on day 9 from mice treated with different therapies. Bar heights are reported as the average ± standard deviation (n = 5 biologically independent mice in one trial). Statistical analysis was carried out with a one-way ANOVA with Tukey's multiple-comparison test. Source data are provided as a Source Data file.

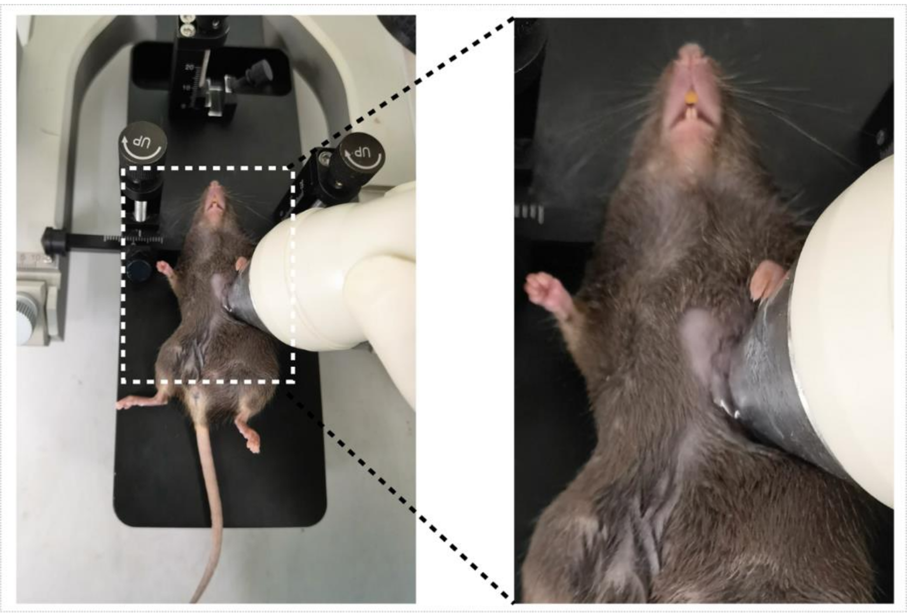

Supplementary Figure 23. Pictures of mice receiving US exposure in sonodynamic therapy. The ultrasound probe was placed snugly on the skin over the stomach of a C57BL/6J mouse. Prior to US exposure, the mouse was subjected to a depilatory treatment on the skin over the stomach. Source data are provided as a Source Data file.

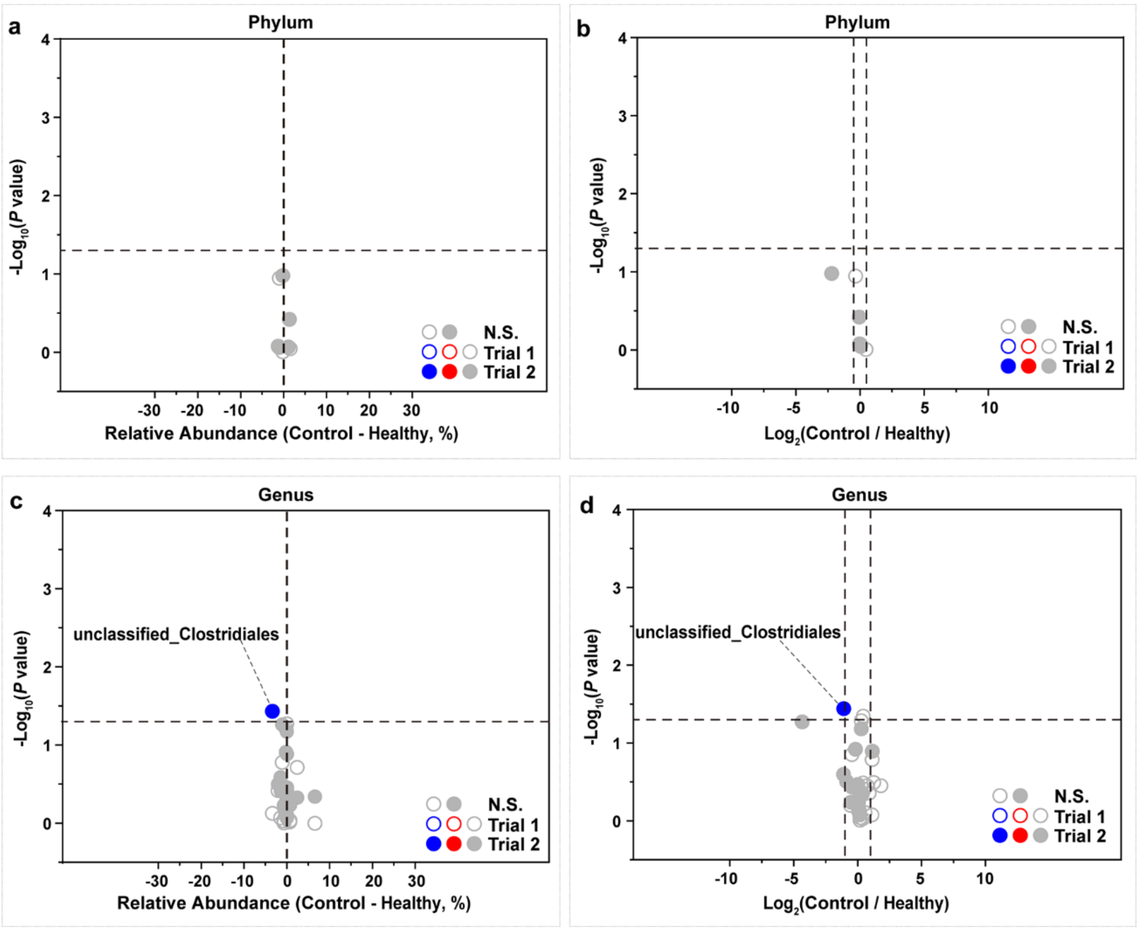

Supplementary Figure 24. Differences in gut microbiota composition between the control *and* healthy groups. The difference in relative abundance of gut bacterial species between the control *versus* healthy groups is indicated both (a, c) as the absolute value of change (*i.e.*, Relative Abundance (Control-Healthy, %)) and (b, d) as the fold of change (*i.e.*, $\log_2$(Control/Healthy)) and plotted separately at the (a-b) phylum and (c-d) genus levels. For a bacterium to be marked as a significantly perturbed bacterium in the plots of $-\log_{10}$(*P* value) *versus* the fold change in bacterial relative abundance, its fold change in bacterial relative abundance needs to be ≥ 2 or $\leq 1/2$, and its *P* value needs to be <0.05. For a bacterium to be marked as a significantly perturbed bacterium in the plots of $-\log_{10}$(*P* value) *versus* the absolute change in bacterial relative abundance, its *P* value needs to be <0.05. Bacterial species whose relative abundances were significantly up- and

downregulated after triple therapy are marked in red and blue, respectively. Statistical analysis was carried out with a two-sided Student's t test. Source data are provided as a Source Data file.

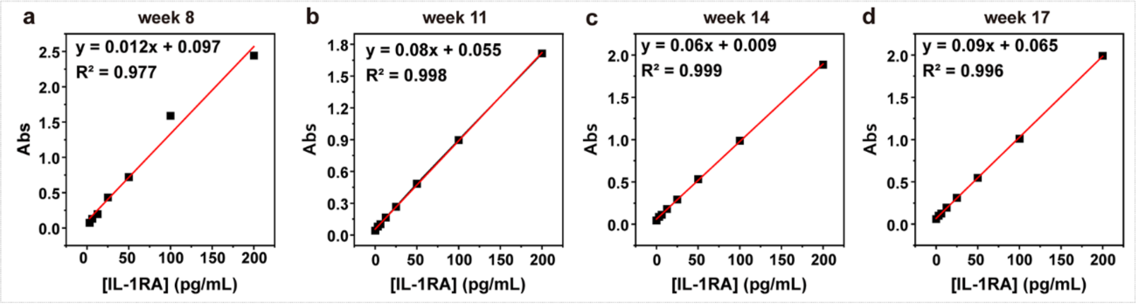

Supplementary Figure 25. Calibration curves for absorbance at 540 nm *versus* IL-1RA concentration. The calibration curves used for quantifying the serum levels of IL1-RA in mouse models at (a) week 8, (b) week 11, (c) week 14, and (d) week 17 during the observation window after the establishment of the *H. pylori*-infected mouse model. Source data are provided as a Source Data file.

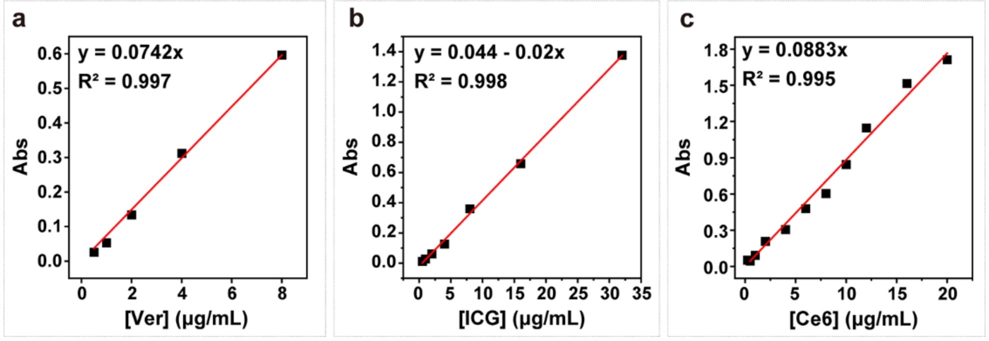

Supplementary Figure 26. Calibration curves for determining the ultrasound sensitizer dose in nanoparticles. a. Calibration curve between the absorbance at 680 nm *versus* the concentration of Ver dissolved in acetone. **b.** Calibration curve between the absorbance at 785 nm *versus* the concentration of ICG dissolved in water. **c.** Calibration curve between the absorbance at 664 nm

versus the concentration of Ce6 dissolved in acetone. Source data are provided as a Source Data file.

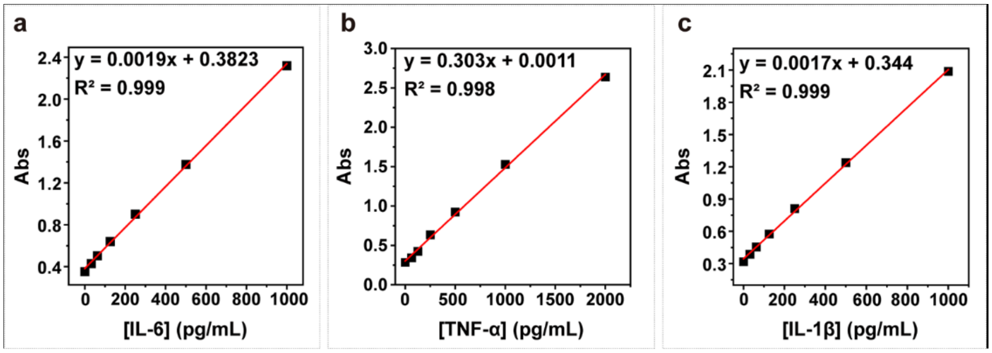

Supplementary Figure 27. Calibration curves for inflammatory factors. a. Calibration curve between absorbance at 540 nm *versus* IL-6 (standard) concentration. **b.** Calibration curve between absorbance at 540 nm *versus* TNF-α (standard) concentrations. **c.** Calibration curve between absorbance at 540 nm *versus* IL-1β (standard) concentrations. Source data are provided as a Source Data file.

Data Availability

Source data are provided with this paper for Supplementary Figs. 1b-e, 2b-d, 3a, b, 4a, b, 5b, 6b, 8b-d, 9b-d, 10b-d, 11a-h, 12a-h, 13a-h, 14a-e, 15a-f, 16b-f, 17b-i, 18b-e, 19a, b, 20b-e, 21a-d, 22a-l, 23, 24a-d, 25a-d, 26a-c, and 27a-c in the associated source data file. Source data are provided with this paper.

METHODS

S1. Preparation of lecithin liposomes.

Briefly, 10 mg lecithin solution (mix phospholipids from soybean, 20 mg/g in chloroform, Macklin Biochemical Co., Ltd, Shanghai, China) was added to a clean glass bottle, followed by addition of 0.5 mg cholesterol (20 mg/g in chloroform). The resulting mixture was then dried to a thin film under a gentle N_2 stream and desiccated under vacuum at room temperature overnight. The resulting thin film was then rehydrated with Millipore water to a final lipid concentration of 4 mg/mL and heated at 40 ºC for 2 hours followed by 7 freeze–thaw cycles. The resulting dispersion was extruded through a nuclear porous membrane with a pore size of 0.2 μm (Whatman) in a mini-extruder (Avanti Polar Lipids) 13 times, which yielded the expected lecithin vesicle. The as-prepared lecithin vesicle was then stored at 4 ºC prior to use.

To determine the hydrodynamic diameter and zeta potential (ζ) for the lecithin liposomes, lecithin liposomes (200-300 μL) were dispersed into Millipore water (~1 mL), and the resulting mixture was characterized by dynamic light scattering (DLS) using a particle size and zeta potential analyzer (NanoBrook, 90Plus PALS, Brookhaven).

S2. Preparation of PLGA@Lecithin nanoparticles.

$PLGA_{40K}$ polymer powder was added into acetone, which yielded a $PLGA_{40K}$ solution at 40 mg/mL. The resulting $PLGA_{40K}$ solution (0.4 mL) was added dropwise into Millipore water (10 mL), and the resulting mixture was stirred at 37 ℃ at 300 rpm for 14 h in open air to evaporate the acetone. The resultant mixture was subsequently subjected to centrifugation (800 g, for 10 min) to remove the large particles and then to centrifugation (10,000 g, for 10 min) to collect the expected PLGA nanoparticles.

To coat the PLGA nanoparticles with a lecithin membrane, a dispersion of PLGA nanoparticles (100 μg/mL in Millipore water) was mixed with the dispersion of lecithin liposomes at a mass ratio of lecithin to PLGA of 1:2, and the resulting mixture was then ultrasonicated (at 53 kHz and at an output power of 100 W) for 5 minutes in a water bath (KUDOS, SK5210HP) and centrifuged (10,000 g, for 10 min) (5417R, Eppendorf) to remove free lecithin liposomes and to collect the expected PLGA@Lecithin nanoparticles. The as-prepared PLGA@Lecithin was then dispersed into Millipore water (to a final concentration of 100 μg/mL unless specified otherwise) and stored at 4 ℃ prior to use.

To determine the morphology and size of the as-prepared PLGA and PLGA@Lecithin, PLGA and PLGA@Lecithin nanoparticles were dispersed into Millipore water at a final concentration of 100 μg/mL, and 2 drops of the resulting nanoparticle dispersion were added onto a copper mesh, followed by the addition of 2 drops of phosphotungstic acid solution (1% in PBS) for staining the lipid membrane, drying at 37 ℃ in an oven for 5 min, and then imaging under TEM (Hitachi h-7650, 100 kV).

To determine the hydrodynamic diameter and zeta potential (ζ) for the PLGA and PLGA@Lecithin nanoparticles, PLGA and PLGA@Lecithin nanoparticles were dispersed into Millipore water to a final concentration of 100 μg/mL, and the resulting mixture was characterized by DLS using a particle size and zeta potential analyzer (NanoBrook, 90Plus PALS, Brookhaven).

S3. Preparation of PLGA@PEG nanoparticles.

$PLGA_{40K}$-PEG_{2K} polymer powder was added to acetone, which yielded a 40 mg/mL $PLGA_{40K}$-PEG_{2K} solution. The resulting $PLGA_{40K}$-PEG_{2K} solution (0.4 mL) was added dropwise into Millipore water (10 mL), and the resulting mixture was stirred at 37 ºC at 300 rpm for 14 h in

open air to evaporate the acetone. The resultant mixture was subsequently subjected to centrifugation (800 g, for 10 min) to remove the large particles and then to centrifugation (10,000 g, for 10 min) to collect the expected PLGA@PEG nanoparticles.

To determine the morphology and size of the as-prepared PLGA@PEG nanoparticles, PLGA@PEG nanoparticles were dispersed into Millipore water at a final concentration of 100 μg/mL, and 2 drops of the resulting nanoparticle dispersion were added onto a copper mesh, dried at 37 ℃ in an oven for 5 min, and then imaged under TEM (Hitachi h-7650, 100 kV).

To determine the hydrodynamic diameter and zeta potential (ζ) for the PLGA@PEG nanoparticles, PLGA@PEG nanoparticles were dispersed into Millipore water to a final concentration of 100 μg/mL, and the resulting mixture was characterized by DLS using a particle size and zeta potential analyzer (NanoBrook, 90Plus PALS, Brookhaven).

S4. Preparation of USS-PLGA nanoparticles.

PLGA nanoparticles were loaded with a USS (verteporfin (Ver), Ce6, or ICG) through a nano-coprecipitation method. Briefly, for Ver-PLGA and Ce6-PLGA nanoparticles, a solution of Ver or Ce6 (1 mL, 1 mg/mL in acetone) was added to the dispersion of $PLGA_{40K}$ solution (0.25 mL, 40 mg/mL in acetone), and the resulting dispersion was added dropwise into Millipore water (10 mL); for ICG-PLGA nanoparticles, a solution of ICG (1 mL, 1 mg/mL in Millipore water) was added into Millipore water (10 mL) and then into the resulting ICG solution (11 mL in Millipore water), was added in dropwise the dispersion of $PLGA_{40K}$ solution (0.25 mL, 40 mg/mL in acetone). The resulting mixtures were subsequently stirred at 37 ºC at 300 rpm for 14 h in open air to evaporate the acetone, and the resultant mixture was then subjected to centrifugation (800 g, for

10 min) to remove large particles and then to centrifugation (10,000 g, for 10 min) to collect the expected USS-PLGA (*i.e.*, Ver-PLGA, Ce6-PLGA, or ICG-PLGA) nanoparticles.

To determine the morphology and size of the as-prepared USS-PLGA nanoparticles, USS-PLGA *(i.e.*, Ce6-PLGA, Ver-PLGA, or ICG-PLGA) nanoparticles were dispersed into Millipore water at a final concentration of 100 μg/mL, and 2 drops of the resulting nanoparticle dispersion were added onto a copper mesh, dried at 37 ℃ in an oven for 5 min, and then imaged under TEM (Hitachi h-7650, 100 kV).

To determine the hydrodynamic diameter and zeta potential (ζ) for the as-prepared USS-PLGA nanoparticles, USS-PLGA (*i.e.*, Ce6-PLGA, Ver-PLGA, or ICG-PLGA) nanoparticles were dispersed into Millipore water to a final concentration of 100 μg/mL, and the resulting mixture was characterized by DLS using a particle size and zeta potential analyzer (NanoBrook, 90Plus PALS, Brookhaven).

S5. Preparation of USS-PLGA@Lecithin nanoparticles.

To coat a USS-PLGA (*i.e.*, Ver-PLGA, Ce6-PLGA, or ICG-PLGA) nanoparticle with a lecithin bilayer membrane, dispersion of the USS-PLGA nanoparticle was mixed with the dispersion of lecithin liposomes at a mass ratio of lecithin to PLGA of 1:2, and the resulting mixture was then ultrasonicated (at 53 kHz and at an output power of 100 W) for 5 minutes in a water bath (KUDOS, SK5210HP) and then centrifuged (10,000 g, for 10 min) (5417R, Eppendorf) to remove free lecithin liposomes and to collect the expected USS-PLGA@Lecithin nanoparticles (*i.e.*, Ver-PLGA@Lecithin, Ce6-PLGA@Lecithin, or ICG-PLGA@Lecithin). The as-prepared USS-PLGA@Lecithin nanoparticles (*i.e.*, Ver-PLGA@Lecithin, Ce6-PLGA@Lecithin, or ICG-

PLGA@Lecithin) were then dispersed into Millipore water (to a final concentration of 100 μg/mL unless specified otherwise) and stored at 4 ℃ prior to use.

To determine the morphology and size of the as-prepared USS-PLGA@Lecithin nanoparticles, USS-PLGA@Lecithin nanoparticles (*i.e.*, Ver-PLGA@Lecithin, Ce6-PLGA@Lecithin, or ICG-PLGA@Lecithin) were dispersed into Millipore water at a final concentration of 100 μg/mL, and 2 drops of the resulting nanoparticle dispersion were added onto a copper mesh, followed by the addition of 2 drops of phosphotungstic acid solution (1% in PBS) to stain the lipid membrane, drying at 37 ℃ in an oven for 5 min, and then imaging under TEM (Hitachi h-7650, 100 kV).

To determine the hydrodynamic diameter and zeta potential (ζ) for the as-prepared USS-PLGA@Lecithin nanoparticles, USS-PLGA@Lecithin nanoparticles (*i.e.*, Ver-PLGA@Lecithin, Ce6-PLGA@Lecithin, or ICG-PLGA@Lecithin) were dispersed into Millipore water to a final concentration of 100 μg/mL, and the resulting mixture was characterized by DLS using a particle size and zeta potential analyzer (NanoBrook, 90Plus PALS, Brookhaven).

To determine the dose of Ver preloaded into Ver-PLGA@Lecithin, the calibration curve between the absorbance at 680 nm *versus* the Ver concentration was obtained by measuring the UV–Vis absorption spectra of Ver at different concentrations in acetone with a UV–Vis spectrophotometer (Cary 60 UV–Vis, Agilent). To determine the Ver content in Ver-PLGA@Lecithin, the Ver-PLGA@Lecithin dispersion (~200 μg/mL in nanoparticle mass concentration, in Millipore water) was freeze-dried with a freeze-dryer (FD-1-50Plus, Biocool), and the resulting solid was then dissolved into acetone (1 mL) for subsequent UV–Vis absorption spectrum measurement with a UV–Vis spectrophotometer (Cary 60 UV–Vis, Agilent), which

yielded an absorbance at 680 nm that, in combination with the calibration curve above, gave us the Ver dose in Ver-PLGA@Lecithin.

To determine the dose of Ce6 preloaded into Ce6-PLGA@Lecithin, the calibration curve between the absorbance at 664 nm *versus* Ce6 concentration was obtained by measuring the UV–Vis absorption spectra of Ce6 at different concentrations in acetone with a UV–Vis spectrophotometer (Cary 60 UV–Vis, Agilent). To determine the Ce6 content in Ce6-PLGA@Lecithin, the Ce6-PLGA@Lecithin dispersion (~200 μg/mL in nanoparticle mass concentration, in Millipore water) was freeze-dried with a freeze-dryer (FD-1-50Plus, Biocool), and the resulting solid was then dissolved in acetone (1 mL) for subsequent UV–Vis absorption spectrum measurement with a UV–Vis spectrophotometer (Cary 60 UV–Vis, Agilent), which yielded the absorbance at 664 nm that, in combination with the calibration curve above, gave us the Ce6 dose in Ce6-PLGA@Lecithin.

To determine the dose of ICG preloaded into ICG-PLGA@Lecithin, the calibration curve between the absorbance at 785 nm *versus* ICG concentration was obtained by measuring the UV–Vis absorption spectra of ICG at different concentrations in water with a UV–Vis spectrophotometer (Cary 60 UV–Vis, Agilent). To determine the ICG content in ICG-PLGA@Lecithin, the ICG-PLGA@Lecithin dispersion (~200 μg/mL in nanoparticle mass concentration, in Millipore water) was freeze-dried with a freeze-dryer (FD-1-50Plus, Biocool), and the resulting solid was then dissolved in water (1 mL) for subsequent UV–Vis absorption spectrum measurement with a UV–Vis spectrophotometer (Cary 60 UV–Vis, Agilent), which yielded an absorbance at 785 nm that, in combination with the calibration curve above, gave us the ICG dose in ICG-PLGA@Lecithin.

S6. Collection of *H. pylori* culture supernatant.

H. pylori culture supernatant (HCS) was obtained by centrifuging the *H. pylori* culture to remove *H. pylori* cells. Briefly, *H. pylori* were removed from a -80 ℃ refrigerator and inoculated into fresh tryptic soy broth (TSB) broth supplemented with fetal bovine serum (FBS) (v./v. of 5%), followed by incubation at 37 ℃ under microaerophilic conditions (10% CO_2, 85% N_2 and 5% O_2) for 12 h, and then centrifugation (10,000 ×g, at 4 ℃, for 10 min) (MicroCL 17R, Thermo Scientific) to collect *H. pylori* cells. The as-collected *H. pylori* cells were redispersed into fresh TSB broth supplemented with FBS (v./v. of 5%), followed by incubation at 37 ℃ for 48 h to mid-log phase (OD_{600} ~ 1.0, which corresponds to ~1.0×10^8 CFU/mL) and then centrifugation (10,000 ×g, at 4 ℃, for 10 min) (MicroCL 17R, Thermo Scientific) to remove the *H. pylori* cells therein, which yielded the supernatant of the resulting *H. pylori* culture. The as-obtained supernatant was then filtered through a syringe filter (pore size of 220 nm, Sinopharm Chemical Reagent Co., Ltd) for sterilization, flash frozen in liquid nitrogen, and freeze-dried in a lyophilizer (FD-1-50Plus, Biocool), which yielded the expected HCS. The as-prepared HCS was stored at 4 ℃ for use within one week.

S7. Characterizations of the kinetics of protein adsorption on nanoparticles.

The content of total adsorbed proteins on a nanoparticle was measured by using a Bradford Protein Assay Kit (Beyotime, China), which indicates protein content with the absorbance at 595 nm due to the binding of Coomassie Blue G-250 with proteins and includes a standard bovine serum albumin (BSA) solution (at 5 mg/mL) for monitoring the calibration curve of absorbance at 595 nm *versus* BSA concentration. Briefly, the standard BSA solution (5 mg/mL) was diluted serially into Millipore water to a series of final concentrations (specifically, at 1.5, 1, 0.75, 0.5,

0.25, and 0.125 mg/mL). The resulting dilutions (5 μL per well) were subsequently added to a 96-well microplate, followed by the addition of Coomassie Blue G-250 staining solution (250 μL per well). The as-set microplate was then placed into a microplate reader (Bio-Rad, iMark) to record the absorbance at 595 nm. Millipore water (*i.e.*, 0 mg/mL BSA) was included as a blank control. Plot of the resulting absorbance (at 595 nm) *versus* protein concentration yielded the calibration curve for BSA, a model for proteins.

To characterize the kinetics of protein adsorption on nanoparticles, a dispersion (100 μL in Millipore water) of a nanoparticle (PLGA@PEG, PLGA@Lecithin, or Ver-PLGA@Lecithin) was mixed with HCS (100 μL in Millipore water) to a final nanoparticle concentration of 10 mg/mL and a final HCS concentration of 10 mg/mL), and the resulting mixture was subsequently incubated at 4 ℃ for a specified time (specifically, 0, 5, 10, 20, 30, 60, 120, 240, 480, 600, or 720 minutes) and then centrifuged (10,000 g, for 10 min) (5417R, Eppendorf) to recollect the nanoparticles, which were then washed twice (10,000 g, for 10 min) (5417R, Eppendorf) with Millipore water. To quantify the amount of adsorbed proteins on nanoparticles after a 12 h incubation in a protein-absent or -present medium, a dispersion (100 μL in Millipore water) of PLGA@PEG or PLGA@Lecithin was mixed with a protein-absent medium (Millipore water, or PBS) (100 μL) or a protein-present medium (HCS-supplemented Millipore water, HCS-supplemented PBS, or HCS-supplemented SGF) (100 μL) to a final nanoparticle concentration of 10 mg/mL and, in the cases with HCS, to a final HCS concentration of 10 mg/mL, and the resulting mixture was subsequently incubated at 4 ℃ for 12 hours and then centrifuged (10,000 g, for 10 min) (5417R, Eppendorf) to recollect the nanoparticles, which were then washed twice (10,000 g, for 10 min) (5417R, Eppendorf) with Millipore water.

The resulting pellet (*i.e.*, the nanoparticle-protein complex) was then redispersed into Millipore water (200 μL), and the resulting dispersion was then added to a 96-well microplate (5 μL per well), followed by the addition of Coomassie blue G-250 staining solution (250 μL per well) and, within 2 hours, determination of the absorbance at 595 nm with a microplate reader (Bio-Rad, iMark). The as-measured absorbance at 595 nm, in combination with the calibration curve of absorbance (595 nm) *versus* BSA concentration measured in the same trial, yielded the concentration of proteins in a well of the microplate, which, combined with the final volume of the particle-protein complex dispersion, provided the total amount of proteins adsorbed on the nanoparticle. Each trial was performed independently in triplicate, and the reported results were the averages of one trial.

S8. Sodium dodecyl sulfate–polyacrylamide gel electrophoresis (SDS–PAGE).

SDS–PAGE was used to detect proteins adsorbed on nanoparticles. Briefly, one-dimensional SDS–PAGE was performed under reducing conditions in 10% acrylamide, and a 10% SDS–PAGE gel was prepared first according to a previously reported protocol[27]. The two glass plates were clamped in the casting frames, and the appropriate amount of separating gel solution (product no. P0012A, Beyotime, China) (preparing 10 mL 10% separating gel solution needs to add 2.7 mL Millipore water, 3.3 mL 30% AcrBis (Acr: Bis ~ 29:1) solution, 3.8 mL Tris (1 M, pH 8.8), 0.1 mL 10% SDS solution, 0.1 mL 10% ammonium persulfate solution, and 4 μL tetramethylethylenediamine) was pipetted into the gap between the glass plates to a level that will enable the comb to be inserted with 5 mm between the bottom of the wells and the top of the separating gel. Then, Millipore water (0.4 mL) was added to cover the separating gel solution to ensure a flat interface between the separating gels and stacking gels, and the gel solution was

allowed to polymerize for 30 min. After discarding the water, the separating gel was left, and the stacking gel solution (product no. P0012A, Beyotime, China) (preparing 4 mL stacking gel solution with 2.7 mL Millipore water, 0.67 mL 30% Acr-Bis (29:1) solution, 0.5 mL 1 M Tris (pH 6.8), 0.04 mL 10% SDS solution, 0.04 mL 10% ammonium persulfate solution and 4 μL tetramethylethylenediamine) was then pipetted into the gap until spilling. The well-forming comb was inserted into the gel solution without trapping air under the teeth. The comb was removed after complete gelation of the stacking gel solution for ~30 min polymerization, yielding the expected SDS–PAGE gel.

Dispersion of PLGA@PEG nanoparticles (50 μL, 80 mg/mL in Millipore water) or PLGA@Lecithin (50 μL, 80 mg/mL in Millipore water) was mixed with HCS (50 μL, 20 mg/mL in Millipore water), and the resulting mixture was incubated at 4 ℃ for 12 h, followed by centrifugation (10,000 g, for 10 min) (5417R, Eppendorf) to discard the supernatant. The resulting pellet was then washed twice with Millipore water (10,000 g, for 10 min) (5417R, Eppendorf), which yielded the nanoparticle-protein complex that was to be redispersed into Millipore water (50 μL). The resulting dispersion (40 μL) was subsequently mixed with 5× SDS–PAGE loading buffer (Beyotime) (10 μL) to solubilize the nanoparticle-associated proteins and then incubated in a hot bath (at 95 ℃) for 5-10 min to denature the proteins. The resulting protein-containing loading buffer was loaded onto a channel of the gel, and electrophoresis was subsequently performed (VE 180, Tanon) at 120 V for 150 min (EPS 300, Tannon) in Tris-Gly electrophoresis buffer (3.02, 18.8 and 1 g/L in Millipore water for tris base, glycine, and SDS, respectively), followed by transferring and immersing SDS–PAGE gel into Coomassie Blue R250 staining solution (Beyotime) for 2 h to stain protein bands. The protein bands were then visualized by washing in decolorization solution (methanol/acetic acid/Millipore water = 3: 1: 6 (in volume ratio)). Finally,

the decolorized gel was photographed with a gel image system (Tannon 1600). Photographs of uncropped and unprocessed SDS–PAGE gels are provided in our Source Data file, which has been provided as a supplementary file for publication.

S9. Cryo-electron microscopy (Cryo-EM).

Cryo-EM was used to characterize the formation of protein coronas on nanoparticles. Briefly, dispersions of PLGA@PEG (100 μL, 60 mg/mL in Millipore water) or PLGA@Lecithin (100 μL, 60 mg/mL in Millipore water) were mixed with Millipore water (100 μL) or HCS-supplemented Millipore water (100 μL, 20 mg/mL), and the resulting mixture was then incubated at 4 °C for 12 h, followed by centrifugation (10,000 g, for 10 min) (5417R, Eppendorf) and washing twice with Millipore water (10,000 g, for 10 min) (5417R, Eppendorf) to recollect the nanoparticles. Next, the recollected nanoparticles were dispersed into Millipore water (to a final concentration of 30 mg/mL, in dose of the particle), and the resulting dispersions were added onto a copper mesh, followed by freezing and then imaging under Cryo-EM (Tecnai G2 SPIRIT 120 kV, Thermo FEI).

S10. Cytotoxicity assays.

Human gastric adenocarcinoma (AGS) cells, the most commonly used gastric epithelial cells in the literature, were used as representative cell lines in this work. Briefly, approximately 6×10^3 AGS cells were seeded into each well of a 96-well microplate and cultured in FBS-supplemented F-12K medium (Procell, PM150910) (v./v. of 10%) at 37 ºC (5% CO_2) for ~24 h to ~80% confluency and then washed with sterile PBS.

The resulting cell culture after washing was then treated with a to-be-tested sample (10 mL in PBS) (more details will be introduced as follows), followed by replenishment with fresh FBS-

supplemented F-12K (90 μL). When the to-be-tested sample was HCS (acid activated), HCS (10 μL in PBS, pH = 7.4) was added into the corresponding wells of the 96-well microplate to differing final concentrations (0 mg/mL, 5 mg/mL, 10 mg/mL, 20 mg/mL, or 40 mg/mL). When the to-be-tested sample was PLGA@PEG, PLGA@PEG dispersion (10 μL in PBS) was added into the corresponding wells of the 96-well microplate to differing final concentrations (0 mg/mL, 2 mg/mL, 6 mg/mL, 10 mg/mL). When the to-be-tested sample was PLGA@Lecithin, PLGA@Lecithin dispersion (10 μL in PBS) was added into the corresponding wells of the 96-well microplate to differing final concentrations (0 mg/mL, 2 mg/mL, 6 mg/mL, and 10 mg/mL). When the to-be-tested sample was a centrifuged supernatant after coincubation of PLGA@PEG with HCS-supplemented PBS (centrifuge (10,000 g, 10 min) after preincubated the PLGA@PEG with HCS-supplemented PBS (pH = 4) for 12 h to collect the supernatant), the centrifuged supernatant (10 μL in PBS, pH = 7.4) was added into the corresponding wells of the 96-well microplate to differing final concentrations (0 mg/mL, 0.5 mg/mL, 1 mg/mL, 1.5 mg/mL, 2 mg/mL for PLGA@PEG, respectively, and 10 mg/mL for HCS). When the to-be-tested sample was a centrifuged supernatant after coincubation of PLGA@PEG with HCS-supplemented PBS (centrifuge (10,000 g, 10 min) after preincubated the PLGA@PEG with HCS-supplemented PBS (pH = 4) for 12 h to collect the supernatant), the centrifuged supernatant (10 μL in PBS, pH = 7.4) was added into the corresponding wells of the 96-well microplate to differing final concentrations (0 mg/mL, 2 mg/mL, 4 mg/mL, 6 mg/mL, 8 mg/mL for PLGA@PEG and 20 mg/mL for HCS). When the to-be-tested sample was a centrifuged supernatant after coincubation of PLGA@Lecithin with HCS-supplemented PBS (centrifuge (10,000 g, 10 min) after preincubating the PLGA@Lecithin with HCS-supplemented PBS (pH = 4) for 12 h to collect the supernatant), the centrifuged supernatant (10 μL in PBS, pH = 7.4) was added into the corresponding wells of

the 96-well microplate to differing final concentrations (0 mg/mL, 0.5 mg/mL, 1 mg/mL, 1.5 mg/mL, 2 mg/mL for PLGA@Lecithin , and 10 mg/mL for HCS). When the to-be-tested sample was a centrifuged supernatant after coincubation of PLGA@Lecithin with HCS-supplemented PBS (centrifuge (10,000 g, 10 min) after preincubating the PLGA@Lecithin with HCS-supplemented PBS (pH = 4) for 12 h to collect the supernatant), the centrifuged supernatant (10 μL in PBS, pH = 7.4) was added into the corresponding wells of the 96-well microplate to differing final concentrations (0 mg/mL, 2 mg/mL, 4 mg/mL, 6 mg/mL, 8 mg/mL for PLGA@Lecithin, and 20 mg/mL for HCS).

The as-treated cells were subsequently incubated at 37 ºC (5% CO_2) for another 24 h and then washed with sterile PBS, followed by cell viability determination with a cell counting kit-8 (CCK-8) by adding CCK-8 (10 μL) and F-12K medium (90 μL) into each well of the 96-well microplate and then incubating the microplate at 37 ºC (5% CO_2) for 1 h. The microplate was then subjected to quantification of optical density at 450 nm (OD_{450}) with a microplate reader (Varioskan, Thermo Scientific).

Controls are cells treated similarly but without any to-be-tested sample. Each trial was carried out in triplicate, and the reported results are averages of two independent trials. The cell viability ratio was defined as the relative ratio of the average OD_{450} reading of cells treated with a to-be-tested sample to that of controls.

S11. Isothermal titration calorimetry (ITC) analysis.

The ITC measurement was performed using a MicroCal PEAQ-ITC (Malvern) with an effective cell volume of 200 μL. The dispersion (20 mg/mL in Millipore water) of a nanoparticle (PLAG@PEG, or PLGA@Lecithin) was added into the sample cell, which was kept at 25 ℃ and

stirred at 750 rpm, while the HCS-supplemented Millipore water solution (40 μL, 30 mg/mL) was added as the injectant into the syringe. The HCS-supplemented Millipore water was then titrated via injection into the nanoparticle dispersion, with the volume of each injection kept constant at 2 μL and the time interval between each two adjacent injections constant at 150 s.

S12. ROS generation by USS-PLGA@Lecithin nanoparticles at differing nanoparticle doses.

2',7'-Dichlorodihydrofluorescein (DCFH) is virtually nonfluorescent, but upon oxidation by ROS, it becomes the bright green fluorescent 2',7'-dichlorofluorescein (DCF)[28]. DCFH was used as the ROS probe for the detection of ROS generation with a fluorimeter. Briefly, NaOH aqueous solution (0.01 M, 400 μL) was added to DCFH-DA solution (1 mM, 100 μL in DMSO), and the resulting mixture was left standing still in the dark for 30 min, followed by the addition of NaH_2PO_4 solution (25 mM, 2 mL in water), which after mixing *by vortexing* yielded the expected DCFH solution (40 μM, assuming 100% conversion of DCFH-DA to DCFH).

The as-prepared DCFH solution (40 μM, 100 μL) was subsequently mixed with a dispersion (100 μL in Millipore water) of a USS-PLGA@Lecithin nanoparticle to different final concentrations (8 μg/mL, 16 μg/mL, 24 μg/mL, 32 μg/mL or 40 μg/mL, in a dose of the corresponding USS (*i.e.*, Ver, Ce6, or ICG)). The resulting mixtures were then ultrasonicated (0.5 W/cm^2, 10 min) with a medical ultrasonic instrument (WED-100, WELLD), followed by centrifugation (10,000 g, at 4 ℃, for 10 min) (5417R, Eppendorf) to collect the nanoparticle-free supernatant for subsequent fluorescence emission spectrum measurement ($\lambda_{ex}/\lambda_{em}$ = 485 nm/500-700 nm, slit-widths of 5 nm for both excitation and emission wavelengths) with a fluorimeter (Hitachi f-4600 fluorescence spectrometer). The control was DCFH treated similarly in PBS but without any USS-PLGA@Lecithin nanoparticles.

S13. Effect of Ver-PLGA@Lecithin surface protein corona formation on ROS production efficiency.

Ver-PLGA@Lecithin nanoparticles (100 μL) were mixed with PBS, HCS-supplemented PBS (100 μL) or HCS-supplemented SGF (100 μL) (to a final concentration of 100 μg/mL for Ver-PLGA@Lecithin nanoparticles (in Ver dose) and 10 mg/mL for HCS) and incubated at 4 ℃ for 12 h. After that, the coincubated mixture was centrifuged (10,000 g, for 10 min) (5417R, Eppendorf) to recollect the nanoparticles and washed twice with Millipore water (10,000 g, for 10 min) (5417R, Eppendorf), and the resulting pellets were then tested for ROS generation under ultrasound conditions.

DCFH was used as the ROS probe for the detection of ROS generation with a fluorimeter. Briefly, DCFH solution (40 μM, 100 μL) was mixed with a dispersion (100 μL, in Millipore water) of Ver-PLGA@Lecithin to a final concentration of 100 μg/mL (at a dose of Ver). The resulting mixtures were then ultrasonicated (0.5 W/cm^2, 10 min) with a medical ultrasonic instrument (WED-100, WELLD), followed by centrifugation (10,000 g, at 4 ℃, for 10 min) (5417R, Eppendorf) to collect the nanoparticle-free supernatant for subsequent fluorescence emission spectrum measurement ($\lambda_{ex}/\lambda_{em}$ = 485 nm/500-700 nm, slit-widths of 5 nm for both excitation and emission wavelengths) with a fluorimeter (Hitachi f-4600 fluorescence spectrometer). The control was DCFH treated similarly in PBS but without any Ver-PLGA@Lecithin nanoparticles.

S14. ROS generation by USS-PLGA@Lecithin upon differing US exposure dosages.

DCFH was used as the ROS probe for the detection of ROS generation with a fluorimeter. Briefly, DCFH solution (40 μM, 100 μL) was mixed with a dispersion (100 μL, in Millipore water) of a USS-PLGA@Lecithin nanoparticle (*i.e.,* Ver-PLGA@Lecithin, Ce6-PLGA@Lecithin, or

ICG-PLGA@Lecithin) to a final concentration of 100 μg/mL (in dose of the corresponding USS (*i.e.*, Ver, Ce6, or ICG)). The resulting mixture was then ultrasonicated at 0.5 W/cm^2 for differing US exposure times (3 min, 9 min, 15 min, 20 min, or 30 min) or for 10 min at differing output power densities (0.3 W/cm^2, 0.5 W/cm^2, 1.0 W/cm^2, or 2.0 W/cm^2) with a medical ultrasonic instrument (WED-100, WELLD), followed by centrifugation (10,000 g, for 10 min) (5417R, Eppendorf) to collect nanoparticle-free supernatant for subsequent fluorescence emission spectrum measurement ($\lambda_{ex}/\lambda_{em}$ = 485 nm/500-700 nm, slit-widths of 5 nm for both excitation and emission wavelengths) with a fluorimeter (Hitachi f-4600 fluorescence spectrometer).

S15. Detection of $^1O_2$ generation by USS-PLGA@Lecithin nanoparticles.

SOSG was used as the probe for detecting the generation of $^1O_2$ with a fluorimeter. Briefly, SOSG solution (10 μM, 100 μL in Millipore water) was mixed with a dispersion (100 μL in Millipore water) of a USS-PLGA@Lecithin nanoparticle (*i.e.*, Ver-PLGA@Lecithin, Ce6-PLGA@Lecithin, or ICG-PLGA@Lecithin) to differing final concentrations (25 μg/mL, 50 μg/mL, 75 μg/mL, or 100 μg/mL in dose of the corresponding USS (*i.e.*, Ver, Ce6, or ICG)). The resulting mixture was then ultrasonicated (0.5 W/cm^2, 10 min) with a medical ultrasonic instrument (WED-100, WELLD), followed by centrifugation (10,000 g, for 10 min) (5417R, Eppendorf) to collect nanoparticle-free supernatant for subsequent fluorescence emission spectrum measurement ($\lambda_{ex}/\lambda_{em}$ = 504 nm/510-700 nm, slit-widths of 5 nm for both excitation and emission wavelengths) with a fluorimeter (Hitachi F-4600 fluorescence spectrometer). The controls were SOSG solution treated similarly in PBS but without any USS-PLGA@Lecithin nanoparticles.

S16. Detection of •OH generation by USS-PLGA@Lecithin nanoparticles.

PTA was used as the probe for detecting the generation of •OH with a fluorimeter. Briefly, PTA solution (1 mM, 100 μL in Millipore water) was subsequently mixed with a dispersion (100 μL in Millipore water) of USS-PLGA@Lecithin (*i.e.*, Ver-PLGA@Lecithin, Ce6-PLGA@Lecithin, or ICG-PLGA@Lecithin) to differing final concentrations (25 μg/mL, 50 μg/mL, 75 μg/mL, or 100 μg/mL, in dose of the corresponding USS (*i.e.*, Ver, Ce6, or ICG)). The resulting mixture was then ultrasonicated (0.5 W/cm^2, for 10 min) with a medical ultrasonic instrument (WED-100, WELLD), followed by centrifugation (10,000 g, for 10 min) (5417R, Eppendorf) to collect nanoparticle-free supernatant for subsequent fluorescence emission spectrum detection ($\lambda_{ex}/\lambda_{em}$ = 315 nm/320-600 nm, slit-widths of 5 nm for both excitation and emission wavelengths) with a fluorimeter (Hitachi f-4600 fluorescence spectrometer). Controls were PTA solution treated similarly but without any USS-PLGA@Lecithin nanoparticles.

S17. Detection of $O^{2-\bullet}$ generation by USS-PLGA@Lecithin nanoparticles.

DHE was used as the probe for detecting $O^{2-\bullet}$ generation with a fluorimeter. Briefly, DHE solution (5 mM, 4 μL) (from the vendor) was diluted with Millipore water to 100 μM, and the resulting DHE dilution (100 μM, 100 μL) was mixed with dispersion (100 μL in Millipore water) of USS-PLGA@Lecithin (*i.e.*, Ver-PLGA@Lecithin, Ce6-PLGA@Lecithin, or ICG-PLGA@Lecithin) to differing final concentrations (25 μg/mL, 50 μg/mL, 75 μg/mL, or 100 μg/mL, in dose of the corresponding USS (*i.e.*, Ver, Ce6, or ICG)). The resultant mixtures were subsequently ultrasonicated (0.5 W/cm^2, 10 min) with a medical ultrasonic instrument (WED-100, WELLD), followed by centrifugation (10,000 g, for 10 min) (5417R, Eppendorf) to collect nanoparticle-free supernatant for subsequent fluorescence emission spectrum measurement

($\lambda_{ex}/\lambda_{em}$ = 480 nm/500-700 nm, slit-widths of 5 nm for both excitation and emission wavelengths) with a fluorimeter (Hitachi f-4600 fluorescence spectrometer). Controls were DHE solution treated similarly but without any USS-PLGA@Lecithin nanoparticle.

S18. Detection of $^1O_2$ generation by USS-PLGA@Lecithin with ESR spectroscopy.

The generation of $^1O_2$ by a USS-PLGA@Lecithin nanoparticle was further monitored with ESR spectroscopy by using TEMP as the trapping agent for capturing $^1O_2$. Briefly, a dispersion (100 μg/mL, 1 mL in water) of a USS-PLGA@Lecithin nanoparticle (*i.e.*, Ver-PLGA@Lecithin, Ce6-PLGA@Lecithin, or ICG-PLGA@Lecithin) was mixed with TEMP (20 μL, 1 mM) (directly from the vendor). The resulting mixture was then ultrasonicated (at 0.5 W/cm^2 for 10 min) with a medical ultrasonic instrument (WED-100, WELLD), followed by ESR spectrum measurement with an ESR spectrometer (JESFA 200, JOEL).

S19. Preparation of the *H. pylori* inoculum for *in vitro* antibacterial assays.

Specifically, *H. pylori* cells were inoculated from a -80 ºC refrigerator into fresh tryptic soy broth (TSB) (2.5 mL) containing 5% (volume ratio) fetal bovine serum (FBS) and cultured at 37 ºC under microaerophilic conditions (10% CO_2, 85% N_2 and 5% O_2) for 12 h, followed by centrifugation (10,000 g, at 4 ºC, for 5 min) (MicroCL 17R, Thermo Scientific) and redispersion of the pellet (*i.e.*, bacterial cells) into fresh FBS-supplemented (v./v. of 5%) TSB (4 mL) and then incubation at 37 ºC for 48 h to allow the bacterial cells to grow to mid-log phase (OD_{600} = 1.0), which yielded an *H. pylori* culture of ~1.0 × 10^8 CFU/mL. The regrown bacterial cells were collected by centrifugation (10,000 g, at 4 ºC, for 5 min) (MicroCL 17R, Thermo Scientific) and washed with sterile PBS *via* centrifugation (10,000 g, at 4 °C, for 5 min) (MicroCL 17R, Thermo

Scientific) twice, and the resulting pellets were redispersed into sterile PBS to ~1.5×10^6 CFU/mL, which yielded the adjusted *H. pylori* suspension for *in vitro* antibacterial assays.

S20. *In vitro* antibacterial assays with USS-PLGA@Lecithin upon 10 min of US exposure at differing output power densities.

The in vitro antibacterial activity of USS-PLGA@Lecithin nanoparticles (*i.e.*, Ver-PLGA@Lecithin, Ce6-PLGA@Lecithin, or ICG-PLGA@Lecithin) against *H. pylori* under different US intensities was evaluated by the plate counting method.

In the cases with US exposure, a 1-mL centrifuge tube was added to a dispersion (100 μL) of a USS-PLGA@Lecithin nanoparticle (*i.e.*, Ver-PLGA@Lecithin, Ce6-PLGA@Lecithin, or ICG-PLGA@Lecithin) at an expected concentration (in dose of the corresponding USS (*i.e.*, Ver, Ce6, or ICG)) and then the adjusted *H. pylori* suspension (50 μL) to achieve a final bacterial inoculation of ~5×10^5 CFU/mL and an expected final USS-PLGA@Lecithin concentration (50 μg/mL, in dose of the corresponding USS (*i.e.*, Ver, Ce6, or ICG)). The resulting mixture was subsequently mixed in the centrifuge tube by pipetting in-and-out and exposed to 10 min of US exposure at differing output power densities (0.3 W/cm^2, 0.5 W/cm^2, 1.0 W/cm^2, or 2.0 W/cm^2) on a medical ultrasonic instrument (WED-100, WELLD) and transferred into a zero-dilution well of a 96-well microplate. In the case without US exposure, USS-PLGA@Lecithin dispersion (100 μL) at an expected concentration (in dose of the corresponding USS (*i.e.*, Ver, Ce6, or ICG)) and the adjusted bacterial suspension (50 μL) were successively added into each zero-dilution well in a 96-well microplate, to achieve a final bacterial inoculation of ~5×10^5 CFU/mL and an expected final USS-PLGA@Lecithin concentration (50 μg/mL, in dose of the corresponding USS (*i.e.*, Ver, Ce6, or ICG)).

In the resulting preset microplate, serial 10-fold dilutions were subsequently made with sterile PBS, followed by plating the resultant dilutions (20 μL) onto Columbia Blood Agar Plate (Columbia agar medium supplemented with sterile defibrinated sheep blood (v./v. of 5%) and then incubated at 37 ºC under microaerophilic conditions (10% CO_2, 85% N_2, and 5% O_2) for 4 days until visible colonies were formed. In each trial, fresh PBS (20 μL) and the adjusted *H. pylori* suspension (20 μL) were plated onto Columbia blood agar plates to verify that the buffer used in the antibacterial assays was sterile and to indicate the inoculum size. Each trial was carried out in triplicate, and the reported results are averages of two independent trials.

S21. *In vitro* antibacterial assays with USS-PLGA@Lecithin upon US exposure at 0.5 W/cm^2 for differing exposure times.

The in vitro antibacterial activity of USS-PLGA@Lecithin nanoparticles (*i.e.*, Ver-PLGA@Lecithin, Ce6-PLGA@Lecithin, or ICG-PLGA@Lecithin) against *H. pylori* upon US exposure for different times was evaluated by the plate counting method.

In the cases with US exposure, a 1-mL centrifuge tube was added to a dispersion (100 μL) of a USS-PLGA@Lecithin nanoparticle (*i.e.*, Ver-PLGA@Lecithin, Ce6-PLGA@Lecithin, or ICG-PLGA@Lecithin) at an expected concentration (in dose of the corresponding USS (*i.e.*, Ver, Ce6, or ICG)), and then the adjusted *H. pylori* suspension (50 μL) (prepared according to **S19. Preparation of the *H. pylori* inoculum for *in vitro* antibacterial assays**) to achieve a final bacterial inoculation of ~5 × 10^5 CFU/mL and an expected final USS-PLGA@Lecithin concentration (50 μg/mL, in dose of the corresponding USS (*i.e.*, Ver, Ce6, or ICG)). The resulting mixture was subsequently mixed in a centrifuge tube by pipetting in-and-out and exposed to US exposure at 0.5 W/cm^2 for differing exposure times (5 min, 10 min, 15 min, or 20 min) on a medical

ultrasonic instrument (WED-100, WELLD), transferred into a zero-dilution well of a 96-well microplate. In the case without US exposure, USS-PLGA@Lecithin dispersion (100 μL) at an expected concentration (in dose of the corresponding USS (*i.e.*, Ver, Ce6, or ICG)) and the adjusted bacterial suspension (50 μL) were successively added into each zero-dilution well in a 96-well microplate to achieve a final bacterial inoculation of ~5×10^5 CFU/mL and an expected final USS-PLGA@Lecithin concentration (50 μg/mL, in dose of the corresponding USS (*i.e.*, Ver, Ce6, or ICG)).

In the resulting preset microplate, serial 10-fold dilutions were subsequently made with sterile PBS, followed by plating the resultant dilutions (20 μL) onto Columbia Blood Agar Plate (Columbia agar medium supplemented with sterile defibrinated sheep blood (v./v. of 5%)) and then incubated at 37 ºC under microaerophilic conditions (10% CO_2, 85% N_2, and 5% O_2) for 4 days until visible colonies were formed. In each trial, fresh PBS (20 μL) and the adjusted *H. pylori* suspension (20 μL) were plated onto Columbia blood agar plates to verify that the buffer used in the antibacterial assays was sterile and to indicate the inoculum size. Each trial was carried out in triplicate, and the reported results are averages of two independent trials.

S22. *In vitro* antibacterial assays with USS-PLGA@Lecithin at differing USS doses.

In vitro anti-*H. pylori* activity of a USS-PLGA@Lecithin nanoparticle (*i.e.*, Ver-PLGA@Lecithin, Ce6-PLGA@Lecithin, or ICG-PLGA@Lecithin) was evaluated by the plate counting method.

In the case with US exposure, a 1-mL centrifuge tube was added to a dispersion (100 μL) of a USS-PLGA@Lecithin nanoparticle (*i.e.*, Ver-PLGA@Lecithin, Ce6-PLGA@Lecithin, or ICG-PLGA@Lecithin) at an expected concentration (in dose of the corresponding USS (*i.e.*, Ver, Ce6,

or ICG)), and then the adjusted *H. pylori* suspension (50 μL) (prepared according to **S19. Preparation of the *H. pylori* inoculum for *in vitro* antibacterial assays**) to achieve a final bacterial inoculation of ~5 × 10^5 CFU/mL and an expected final USS-PLGA@Lecithin concentration (0 μg/mL, 25 μg/mL, 50 μg/mL, 75 μg/mL, or 100 μg/mL, in dose of the corresponding USS (*i.e.*, Ver, Ce6, or ICG)). The resulting mixture was subsequently mixed in the centrifuge tube by pipetting in-and-out, exposed to US (0.5 W/cm^2, 10 min) on a medical ultrasonic instrument (WED-100, WELLD) and transferred into a zero-dilution well of a 96-well microplate. In the case without US exposure, USS-PLGA@Lecithin dispersion (100 μL) at an expected concentration (in dose of the corresponding USS (*i.e.*, Ver, Ce6, or ICG)) and the adjusted bacterial suspension (50 μL) were successively added into each zero-dilution well in a 96-well microplate to achieve a final bacterial inoculation of ~5 × 10^5 CFU/mL and an expected final USS-PLGA@Lecithin concentration (0 μg/mL, 25 μg/mL, 50 μg/mL, 75 μg/mL, or 100 μg/mL, in dose of the corresponding USS (*i.e.*, Ver, Ce6, or ICG)).

In the resulting preset microplate, serial 10-fold dilutions were subsequently made with sterile PBS, followed by plating the resultant dilutions (20 μL) onto Columbia Blood Agar Plate (Columbia agar medium supplemented with sterile defibrinated sheep blood (v./v. of 5%)) and then incubation at 37 ºC under microaerophilic conditions (10% CO_2, 85% N_2, and 5% O_2) for 4 days until visible colonies were formed. In each trial, fresh PBS (20 μL) and the adjusted *H. pylori* suspension (20 μL) were plated onto Columbia blood agar plates to verify that the buffer used in the antibacterial assays was sterile and to indicate the inoculum size. Each trial was carried out in triplicate, and the reported results are averages of two independent trials.

S23. *In vitro* antibacterial assays with Ver-PLGA@Lecithin.

We evaluated the *in vitro* antibacterial activity of Ver-PLGA@Lecithin against *H. pylori* (ATCC 43504) by the plate counting method. In the case of US exposure, Ver-PLGA@Lecithin dispersion (100 μL) was added to a 1-mL centrifuge tube at an expected concentration (in Ver dose), and then the adjusted *H. pylori* suspension (50 μL) (prepared according to **S19. Preparation of the *H. pylori* inoculum for *in vitro* antibacterial assays**) to achieve a final bacterial inoculation of ~5 × 10^5 CFU/mL and an expected final Ver-PLGA@Lecithin concentration (0 μg/mL, 25 μg/mL, 50 μg/mL, 75 μg/mL, or 100 μg/mL in Ver dose). The resulting mixture was subsequently mixed in the centrifuge tube by pipetting in-and-out, exposed to US (0.5 W/cm^2, 10 min) on a medical ultrasonic instrument (WED-100, WELLD) and transferred into a zero-dilution well of a 96-well microplate. In the case without US exposure, Ver-PLGA@Lecithin dispersion (100 μL) at an expected concentration (in Ver dose) and the adjusted bacterial suspension (50 μL) were successively added into each zero-dilution well in a 96-well microplate to achieve a final bacterial inoculation of ~5 × 10^5 CFU/mL and an expected final Ver-PLGA@Lecithin concentration (0 μg/mL, 25 μg/mL, 50 μg/mL, 75 μg/mL, or 100 μg/mL in Ver dose).

In the resulting preset microplate, serial 10-fold dilutions were subsequently made with sterile PBS, followed by plating the resultant dilutions (20 μL) onto Columbia Blood Agar Plate (Columbia agar medium (Qingdao Hope Bio-Technology, China)) supplemented with sterile defibrinated sheep blood (v./v. of 5%, Bianjian Biotechnology Co., Ltd.) and then incubated at 37 ºC under microaerophilic conditions (10% CO_2, 85% N_2, and 5% O_2) for 4 days until visible colonies were formed. In each trial, fresh PBS (20 μL) and the adjusted *H. pylori* suspension (20 μL) were plated onto Columbia blood agar plates to verify that the buffer used in the antibacterial assays was sterile and to indicate the inoculum size. Each trial was carried out in triplicate, and the

reported results are averages of two independent trials.

S24. *In vitro* antibacterial assays with Ver-PLGA@Lecithin in simulated gastric fluid.

The *in vitro* antibacterial activity of Ver-PLGA@Lecithin against *H. pylori* was monitored in simulated gastric fluid (SGF) (Yuanye Bio-Technology, Shanghai, China). In the case of US exposure, Ver-PLGA@Lecithin dispersion (100 μL, in SGF) was added to a 1-mL centrifuge tube at an expected concentration (in dose of the Ver), and then the adjusted *H. pylori* suspension (50 μL) (prepared according to **S19. Preparation of the *H. pylori* inoculum for *in vitro* antibacterial assays**) to achieve a final bacterial inoculation of ~5 × 10^5 CFU/mL and an expected final Ver-PLGA@Lecithin concentration (0 μg/mL, 25 μg/mL, 50 μg/mL, 75 μg/mL, or 100 μg/mL in dose of the Ver). The resulting mixture was subsequently mixed in the centrifuge tube by pipetting in-and-out, exposed to US (0.5 W/cm^2, 10 min) on a medical ultrasonic instrument (WED-100, WELLD) and transferred into a zero-dilution well of a 96-well microplate. In the case without US exposure, Ver-PLGA@Lecithin dispersion (100 μL, in SGF) at an expected concentration (in dose of the Ver) and the adjusted bacteria suspension (50 μL) were successively added into each zero-dilution well in a 96-well microplate to achieve a final bacterial inoculation of ~5 × 10^5 CFU/mL and an expected final Ver-PLGA@Lecithin concentration (0 μg/mL, 25 μg/mL, 50 μg/mL, 75 μg/mL, or 100 μg/mL in dose of the Ver).

In the resulting preset microplate, serial 10-fold dilutions were subsequently made with sterile SGF, followed by plating the resultant dilutions (20 μL) onto Columbia Blood Agar Plate (Columbia agar medium supplemented with sterile defibrinated sheep blood (v./v., 5%)) and then incubation at 37 °C under microaerophilic conditions (10% CO_2, 85% N_2, and 5% O_2) for 4 days until visible colonies were formed. In each trial, fresh SGF (20 μL) and the adjusted *H. pylori*

suspension (20 μL) were plated onto Columbia blood agar plates to verify that the buffer used in the antibacterial assays was sterile and to indicate the inoculum size. Each trial was carried out in triplicate, and the reported results are averages of two independent trials.

S25. Effect of Ver-PLGA@Lecithin surface protein corona formation on antibacterial efficiency *in vitro*.

Ver-PLGA@Lecithin nanoparticles (100 μL) were mixed with PBS, SGF, HCS-supplemented PBS (100 μL) or HCS-supplemented SGF (100 μL) (to a final concentration of 100 μg/mL for Ver-PLGA@Lecithin nanoparticles (in Ver dose) and 10 mg/mL for HCS) and incubated at 4 ℃ for 12 h. After that, the mixture was centrifuged (10,000 g, for 10 min) (5417R, Eppendorf) to recollect the nanoparticles and washed twice with Millipore water (10,000 g, for 10 min) (5417R, Eppendorf). The resulting pellets were then tested for *in vitro* antibacterial activity under ultrasound conditions.

In the case with US exposure, a 1-mL centrifuge tube was added to a dispersion (100 μL) of a Ver-PLGA@Lecithin nanoparticle at an expected concentration (in dose of the Ver) and then the adjusted *H. pylori* suspension (50 μL) (prepared according to **S19. Preparation of the *H. pylori* inoculum for *in vitro* antibacterial assays**) to achieve a final bacterial inoculation of ~5×10^5 CFU/mL and an expected final Ver-PLGA@Lecithin concentration (100 μg/mL, in dose of the Ver). The resulting mixture was subsequently mixed in the centrifuge tube by pipetting in-and-out, exposed to US (0.5 W/cm^2, 10 min) on a medical ultrasonic instrument (WED-100, WELLD) and transferred into a zero-dilution well of a 96-well microplate. In the case without US exposure, Ver-PLGA@Lecithin dispersion (100 μL) at an expected concentration (in dose of the Ver) and the adjusted bacteria suspension (50 μL) were successively added into each zero-dilution well in a 96-

well microplate to achieve a final bacterial inoculation of ~5 × 10^5 CFU/mL and an expected final Ver-PLGA@Lecithin concentration (100 μg/mL, in dose of the Ver).

In the resulting preset microplate, serial 10-fold dilutions were subsequently made with sterile PBS, followed by plating the resultant dilutions (20 μL) onto Columbia Blood Agar Plate (Columbia agar medium supplemented with sterile defibrinated sheep blood (v./v., 5%)) and then incubation at 37 °C under microaerophilic conditions (10% CO_2, 85% N_2, and 5% O_2) for 4 days until visible colonies were formed. In each trial, fresh PBS (20 μL) and the adjusted *H. pylori* suspension (20 μL) were plated onto Columbia blood agar plates to verify that the buffer used in the antibacterial assays was sterile and to indicate the inoculum size. Each trial was carried out in triplicate, and the reported results are averages of two independent trials.

S26. *In vitro* antibacterial assays with Ver-PLGA@Lecithin in the presence of β-carotene.

The role of $^1O_2$ generated *in situ* by Ver-PLGA@Lecithin upon US exposure in the activity of the particles against *H. pylori* was examined by antibacterial assays in the presence of β-carotene.

In the case of US exposure, Ver-PLGA@lecithin dispersion (100 μL, in PBS containing 2.25 mg/mL β-carotene) at an expected concentration (in dose of the Ver) and the adjusted *H. pylori* suspension (50 μL) (prepared according to **S19) were successively added to a 1-mL centrifuge tube. Preparation of the *H. pylori* inoculum for *in vitro* antibacterial assays**), which yielded a mixture of ~5 × 10^5 CFU/mL *H. pylori* cells and Ver-PLGA@Lecithin at an expected final concentration (0 μg/mL, 25 μg/mL, 50 μg/mL, 75 μg/mL, or 100 μg/mL, in dose of the Ver) in PBS containing β-carotene at a final concentration of 1.5 mg/mL. The resulting mixture was subsequently mixed in a centrifuge tube by pipetting in-and-out and exposed to US (at 0.5 W/cm^2, for 10 min) on a medical ultrasonic instrument (WED-100, WELLD) and transferred into a zero-

dilution well of a 96-well microplate. In the case without US exposure, Ver-PLGA@Lecithin dispersion (100 μL) at an expected concentration (in dose of the Ver) and the adjusted bacteria suspension (50 μL) were successively added into each zero-dilution well in a 96-well microplate, which yielded a mixture of ~5 × 10^5 CFU/mL *H. pylori* cells and Ver-PLGA@Lecithin at an expected final concentration (0 μg/mL, 25 μg/mL, 50 μg/mL, 75 μg/mL, or 100 μg/mL, in dose of the Ver) in PBS containing β-carotene at a final concentration of 1.5 mg/mL.

In the resulting preset microplate, serial 10-fold dilutions were subsequently made with sterile PBS, followed by plating the resultant dilutions (20 μL) onto Columbia Blood Agar Plate (Columbia agar medium supplemented with sterile defibrinated sheep blood (v./v., 5%)) and then incubation at 37 ºC under microaerophilic conditions (10% CO_2, 85% N_2, and 5% O_2) for 4 days until visible colonies were formed. In each trial, fresh PBS (20 μL, containing 1.5 mg/mL β-carotene) and the adjusted *H. pylori* suspension (20 μL) were plated onto Columbia blood agar plates to verify that the buffer used in the antibacterial assays was sterile and to indicate the inoculum size. Controls are *H. pylori* cells treated similarly but in the absence of β-carotene. Each trial was carried out in triplicate, and the reported results are averages of two independent trials.

S27. SEM characterization of *H. pylori* cells after different treatments.

H. pylori cells were inoculated from a -80 ºC refrigerator into fresh TSB (2.5 mL) containing 5% (volume ratio) FBS and cultured at 37 ºC under microaerophilic conditions (10% CO_2, 85% N_2 and 5% O_2) for 12 h, followed by centrifugation (10,000 g, at 4 ºC, for 5 min) (MicroCL 17R, Thermo Scientific) and redispersion of the pellet (*i.e.*, bacterial cells) into fresh FBS-supplemented (v./v. of 5%) TSB (4 mL) and then incubation at 37 ºC for 48 h to allow the bacterial cells to grow to mid-log phase (OD_{600} = 1.0), which yielded an *H. pylori* culture of ~1.0 × 10^8 CFU/mL. The

regrown bacterial cells were collected by centrifugation (10,000 g, at 4 ºC, for 5 min) (MicroCL 17R, Thermo Scientific) and washed with sterile PBS *via* centrifugation (10,000 g, at 4 ºC, for 5 min) (MicroCL 17R, Thermo Scientific) twice, and the resulting pellets were redispersed into sterile PBS to ~1.0×10^7 CFU/mL, which yielded the adjusted *H. pylori* suspension.

Into a 1-mL centrifuge tube was added Ver-PLGA@Lecithin dispersion (200 μL) at an expected concentration (in dose of the Ver) and then the adjusted *H. pylori* suspension (200 μL), to achieve a final bacterial inoculation of ~5×10^6 CFU/mL and a final Ver-PLGA@Lecithin concentration of 1 mg/mL (in dose of the Ver). The resulting mixture was subsequently mixed in the centrifuge tube by pipetting in-and-out, exposed to US (0.5 W/cm^2, 10 min) on a medical ultrasonic instrument (WED-100, WELLD) and then centrifuged (10,000 g, at 4 ℃, for 5 min) (MicroCL 17R, Thermo Scientific). The resulting pellet was redispersed into formaldehyde (1 mL, 4%), followed by standing still for 1 h, successive dehydration with ethanol solutions at a series of concentration gradients (25%, 50%, 75%, 90% and 100%) (5-min dehydration with each ethanol solution and then centrifugation (10,000 g, at 4 ℃, for 5 min) (MicroCL 17R, Thermo Scientific) to remove the supernatant), and then redispersion into 100% ethanol (100 μL). The resulting dispersion (10 μL) was dropped onto a copper sheet, dried at 37 °C for 5 min, sputtered with gold-platinum alloy for 10 min, and then imaged under an SEM microscope (FEI Apreo, Thermo Scientific).

Controls are *H. pylori* cells treated similarly but in the absence of both US and Ver-PLGA@Lecithin. The SEM characterizations were repeated at least once to confirm that the results are reproducible.

S28. Biocompatibility of orally administered Ver-PLGA@Lecithin in healthy mice.

We established mouse models and monitored the biocompatibility of orally administered Ver-PLGA@Lecithin in healthy mice, with PBS administered alone for comparison. All animal experiments were conducted in compliance with the guidelines for the care and use of research animals established by the Animal Care and Use Committee at the University of Science and Technology of China (USTCACUC1501010). Mice were housed at a temperature of 22-25 ºC and a 12 h/12 h dark/light cycle. The mouse models used in this work were of one sex, which is female. Briefly, ten C57BL/6J female mice (8 weeks old, Shanghai Slack Laboratory Animal Technology Co., Ltd.) were randomly divided into 2 groups (n = 5 per group), with one group receiving oral administration of PBS throughout the whole assay (*i.e.*, the healthy group) and the other group receiving oral administration of Ver-PLGA@Lecithin (*i.e.*, the Ver-PLGA@Lecithin group). For the healthy group, each mouse was treated with PBS (0.3 mL each time) *via* oral administration every 24 hours and 6 times (gavage on days 1, 2, 3, 4, 5, and 6). For the Ver-PLGA@Lecithin group, each mouse was treated *via* oral administration of Ver-PLGA@Lecithin (0.3 mL of 10 mg/mL in PBS each time) every 24 hours 6 times (gavage on days 1, 2, 3, 4, 5, and 6).

At 24 h after treatment completion, blood samples were collected from the mice of each group above, all mice in the two groups were sacrificed, and their stomachs, hearts, livers, spleens, lungs and kidneys were collected from the abdominal cavity. Briefly, blood was collected from the orbits of mice into a 1.5-mL centrifuge tube containing 10 μL of 100 U heparin sodium solution (Changzhou Qianhong Biopharma Co., Ltd. China) and then temporarily stored on ice. Each as-collected blood sample was subsequently subjected to centrifugation (at 1,500 × g for 10 min at 4 °C) (5417R, Eppendorf), and the resulting supernatant, which was mouse serum, was collected, stored in an aliquot (200 μL each aliquot) at -80 °C, and used within one week. To quantify the

levels of interleukin-1 beta (IL-1β) and interleukin-6 (IL-6), two proinflammatory factors, in the resulting mouse serum samples, two ELISA kits (Beyotime Biotechnology) for IL-1β and IL-6 were used. Briefly, an aliquot of as-collected serum (200 μL) was removed from the -80 ºC freezer and warmed naturally to room temperature, and into a well in a 96-well microplate of an ELISA kit were successively added 10 μL of the resulting serum and 90 μL PBS, followed by incubation at 37 ºC for 2 h, washing with wash buffer of the ELISA kit 3 times, and then the addition of horseradish peroxidase-labelled detection antibody and incubation at 37 ºC for 1 h. The resulting sample was subsequently washed again with wash buffer of the ELISA kit 3 times, followed by the addition of the substrate 3,3',5,5'-tetramethylbenzidine (TMB), incubation at 37 ºC in the dark for 20 min, and then the addition of the stop solution of the ELISA kit to terminate the reaction. The microplate was then subjected to optical density reading at 540 nm (OD_{540}) with a microplate reader (Varioskan, Thermo Scientific). For each proinflammatory factor (*i.e.*, IL-1β or IL-6), the calibration curve of OD_{540} *versus* its concentration was determined using the standard solution provided in the ELISA kit by the vendor. Each trial was performed in triplicate, and the reported results are the averages of two independent trials.

For the mice in each group, all five mice were used for histological analysis. The mouse stomachs and organs collected at 24 h after treatment completion were then cut open and rinsed with PBS to remove the contents therein. For histological analysis, mouse gastric and organ tissues from each group were fixed in 10% neutral buffered formalin, processed routinely into paraffin, sectioned, and stained with hematoxylin and eosin (H&E) for imaging under a microscope (MuVi-SPIM, LUXENDO).

S29. Examinations on toxicity for sonodynamic therapy in mouse models bearing gastric *H.*

***pylori* infection.**

To evaluate the *in vivo* biosafety of sonodynamic therapy mediated by Ver-PLGA@Lecithin, we established mouse models bearing gastric infection with *H. pylori* and monitored the efficacy of the sonodynamic therapy mediated by Ver-PLGA@Lecithin. All animal experiments were conducted in compliance with the guidelines for the care and use of research animals established by the Animal Care and Use Committee at the University of Science and Technology of China (USTCACUC1501010). Mice were housed at a temperature of 22-25 ºC and a 12 h/12 h dark/light cycle. The mouse models used in this work were of one sex, which is female. Briefly, thirty C57BL/6J female mice (8 weeks old, Shanghai Slack Laboratory Animal Technology Co., Ltd.) were randomly divided into 6 groups (n = 5 per group), with one group to stay uninfected throughout the whole assay (*i.e.*, the healthy group) while the remaining five groups were infected *via* gavage administration of *H. pylori* containing FBS-supplemented TSB (0.3 mL of ~1.0×10^8 CFU/mL for each mouse) every 48 hours 4 times (on days 1, 3, 5, and 7, respectively) and fasting for 12 hours prior to bacterial inoculation in mice and gavage $NaHCO_3$ (0.15 mL 0.2 M) in the first 10 minutes of the day of bacterial inoculation to increase gastric pH to improve the survival and colonization rates of *H. pylori* without decomposing urea and establishing resistance to strong acids) and then left alone for 2 weeks to allow *H. pylori* infection to be established. Subsequently, the healthy group was treated with PBS (*i.e.*, healthy), and the five infected groups were treated with PBS (*i.e.*, control), triple therapy, Ver-PLGA@Lecithin alone, US exposure alone, and Ver-PLGA@Lecithin plus US. For both the healthy group and the control group, each mouse was treated with PBS (0.3 mL each time) *via* oral administration every 48 hours and 4 times (gavage on days 1, 3, 5, and 7). For the triple therapy group, each mouse was treated *via* oral administration with a triple therapy regimen every 48 hours 4 times (gavage on days 1, 3, 5, and 7, respectively),

and at each time, the regimen was administered omeprazole (a proton pump inhibitor, Macklin Biochemical Co., Ltd.) (400 μmol per kilogram of mouse weight) and, 30 minutes later, with amoxicillin (28.5 mg/kg, Sigma–Aldrich) and clarithromycin (14.3 mg/kg, Sigma–Aldrich). For the Ver-PLGA@Lecithin group, each mouse was treated *via* oral administration of Ver-PLGA@Lecithin (0.3 mL of 10 mg/mL in PBS each time) every 48 hours 4 times (gavage on days 1, 3, 5, and 7). For the US group, each mouse was treated with a combination of PBS and US exposure every 48 hours 4 times (gavage on days 1, 3, 5, and 7, respectively), and at each time, the treatment was performed with PBS *via* oral administration (0.3 mL each time) and, 30 min later, with US exposure (0.5 W/cm^2 for 10 min) with a medical US instrument (WED-100, WELLD) on the skin over the stomach. For the sonodynamic therapy group, each mouse was treated with a combination of Ver-PLGA@Lecithin and US exposure every 48 hours 4 times (gavage on days 1, 3, 5, and 7) and, at each time, the treatment was performed with Ver-PLGA@Lecithin (0.3 mL of 10 mg/mL in PBS) *via* oral administration and, 30 min later, with US exposure (0.5 W/cm^2, 10 min) with a medical US instrument (WED-100, WELLD) on the skin over stomach.

At 48 h after treatment completion, blood samples were collected from all of the mice. Briefly, blood was collected from the orbits of mice into a 1.5-mL centrifuge tube containing 10 μL of 100 U heparin sodium solution (Changzhou Qianhong Biopharma Co., Ltd. China) and then temporarily stored on ice. After that, 50 μL of each blood sample was immediately used to detect blood biochemical indicators with an animal hematology analyzer (XT-1800i, Sysmex). The rest of each as-collected blood sample was subsequently subjected to centrifugation (1,500 g, at 4 ºC, for 10 min) (5417R, Eppendorf), and the resulting supernatant, which was mouse serum, was collected, stored in an aliquot (200 μL each aliquot) at -80 °C, and used within one week. To quantify the levels of albumin, alanine aminotransferase (ALT) and aspartate aminotransferase

(AST), three liver and renal function markers, in the resulting mouse serum samples, three ELISA kits (catalog no. CEB028Mu, SEA207Mu and SEB214Mu, respectively, Cloud-Clone Corp. Wuhan Co., Ltd. China), respectively, for albumin, ALT and AST were used. Briefly, an aliquot of as-collected serum (200 μL) was removed from the -80 ºC freezer and warmed naturally to room temperature, and 10 μL of the resulting serum and 90 μL PBS were successively added to a well in a 96-well microplate of an ELISA kit, followed by incubation at 37 ºC for 1 h, washing with wash buffer of the ELISA kit 3 times, and then the addition of horseradish peroxidase-labelled detection antibody and incubation at 37 °C for 1.5 h (AST and ALT) or 30 min (albumin). The resulting sample was subsequently washed again with wash buffer from the ELISA kit 3 times, followed by the addition of the substrate 3,3',5,5'-tetramethylbenzidine (TMB), incubation at 37 ºC in the dark for 10 min, and then the addition of the stop solution from the ELISA kit to terminate the reaction. The microplate was then subjected to optical density reading at 540 nm (OD_{540}) with a microplate reader (Varioskan, Thermo Scientific). To quantify the levels of creatinine (CREA), blood urea nitrogen (BUN) and uric acid (UA), three liver and renal function markers, in the resulting mouse serum samples, three ELISA kits (Catalog no. ml037580, ml076479 and ml092697, Shanghai Enzyme-linked Biotechnology Co., Ltd. (Shanghai, China)), respectively, for CREA, BUN and UA were used. The experimental method is consistent with the above. For each liver and renal function marker, the calibration curve of OD_{540} *versus* its concentration was determined using the standard solution provided in the ELISA kit by the vendor. Each trial was performed in triplicate, and the reported results are the averages of two independent trials.

For TUNEL (TdT-mediated dUTP nick-end labelling) analysis, mouse gastric tissues (n = 2) from each group were fixed in 10% neutral buffered formalin and sent to XINLE Biotech Co., Ltd. (Anhui, China) for TUNEL analysis under a microscope (LSM980, ZEISS). For samples from

each group, we imaged the TUNEL-stained tissue slices under a fluorescence microscope at 50 different fields of view and at 32× magnification. The resultant fluorescence microscopy images were then subjected to statistical analysis with ImageJ (ImageJ2) software, which was launched directly without manual addition of any plugins. In the analysis using ImageJ, the images were thresholded in the 'default' mode coupled with 'dark background' and 'auto', to avoid possible image-to-image errors introduced by manual selection of threshold. Once an image was thresholded, individual cells in that image could be identified.

For a fluorescence TUNEL-stained image, the mean intensity of green fluorescence and that of blue fluorescence over the whole image were obtained as specific values from ImageJ, and the percentage of the green fluorescence intensity for that image was calculated with the function below.

$$\text{Percentage of green fluorescence intensity} = I_{\text{Green}} / (I_{\text{Green}} + I_{\text{Blue}})$$

where I_{Green} and I_{Blue} represent the mean intensity of green fluorescence and the mean intensity of blue fluorescence over a whole TUNEL-stained image, respectively.

In this way, we calculated the percentage of the average green fluorescence intensity for each of the 300 fluorescence images (*i.e.*, 6 groups, with 50 images for each group). Moreover, for the 50 images for each group, we calculated the average and standard deviation for their percentages of green fluorescence intensity.

S30. Quantifying the serum levels of IL-1RA in mouse models.

We monitored the serum levels of IL-1RA in mouse models bearing gastric *H. pylori* infection. All animal experiments were conducted in compliance with the guidelines for the care and use of research animals established by the Animal Care and Use Committee at the University of Science

and Technology of China (USTCACUC1501010). The mouse models used in this work were of one sex, which is female. Briefly, thirty C57BL/6J female mice (8 weeks old, Shanghai Slack Laboratory Animal Technology Co., Ltd.) were randomly divided into 6 groups (n = 5 per group), with one group to stay uninfected throughout the whole assay (*i.e.*, the healthy group) while the remaining five groups were infected *via* gavage administration of *H. pylori* containing FBS-supplemented TSB (0.3 mL of ~1.0×10^8 CFU/mL for each mouse) every 48 hours 4 times (on days 1, 3, 5, and 7, respectively) and then left alone for 2 weeks to allow *H. pylori* infection to be established. Subsequently, the healthy group was treated with PBS (*i.e.*, healthy), and the five infected groups were treated with PBS (*i.e.*, control), triple therapy, Ver-PLGA@Lecithin alone, US exposure alone, and Ver-PLGA@Lecithin plus US. For both the healthy group and the control group, each mouse was treated with PBS (0.3 mL each time) *via* oral administration every 48 hours and 4 times (gavage on days 1, 3, 5, and 7). For the triple therapy group, each mouse was treated *via* oral administration with a triple therapy regimen every 48 hours 4 times (gavage on days 1, 3, 5, and 7, respectively), and at each time, the regimen was administered omeprazole (a proton pump inhibitor, Macklin Biochemical Co., Ltd.) (400 μmol per kilogram of mouse weight) and, 30 minutes later, with amoxicillin (28.5 mg/kg, Sigma–Aldrich) and clarithromycin (14.3 mg/kg, Sigma–Aldrich). For the Ver-PLGA@Lecithin group, each mouse was treated *via* oral administration of Ver-PLGA@Lecithin (0.3 mL of 10 mg/mL in PBS each time) every 48 hours 4 times (gavage on days 1, 3, 5, and 7). For the US group, each mouse was treated with a combination of PBS and US exposure every 48 hours 4 times (gavage on days 1, 3, 5, and 7, respectively), and at each time, the treatment was performed with PBS *via* oral administration (0.3 mL each time) and, 30 min later, with US exposure (0.5 W/cm^2 for 10 min) with a medical US instrument (WED-100, WELLD) on the skin over the stomach. For the sonodynamic therapy group, each mouse was

treated with a combination of Ver-PLGA@Lecithin and US exposure every 48 hours 4 times (gavage on days 1, 3, 5, and 7) and, at each time, the treatment was performed with Ver-PLGA@Lecithin (0.3 mL of 10 mg/mL in PBS) *via* oral administration and, 30 min later, with US exposure (0.5 W/cm^2, 10 min) with a medical US instrument (WED-100, WELLD) on the skin over stomach.

Mouse blood collection was performed three weeks after triple therapy and sonodynamic therapy, and blood was collected in the orbits of mice into a 1.5-mL centrifuge tube containing 10 μL of 100 U heparin sodium solution (Changzhou Qianhong Biopharma Co., Ltd. China) and then temporarily stored on ice. Each as-collected blood sample was subsequently subjected to centrifugation (at 1,500 × g for 10 min at 4 ºC) (5417R, Eppendorf), and the resulting supernatant, which was mouse serum, was collected, stored in an aliquot (200 μL each aliquot) at -80 ºC, and used within one week. To quantify the levels of IL-1RA in the resulting mouse serum samples, an enzyme-linked immunosorbent assay (ELISA) kit (Catalog no. SEA223Mu, Cloud-Clone Corp. Wuhan Co., Ltd., China) were used to detect the content of IL-1RA in the serum. Briefly, an aliquot of as-collected serum (200 μL) was removed from the -80 ºC freezer, warmed naturally to room temperature, and placed into a well in a 96-well microplate of an ELISA kit. The kit precoated with IL-1RA antibody was successively added, followed by incubation at 37 ºC for 1 h, washing with wash buffer of the ELISA kit 3 times, and then the assay solution was added and incubated at 37 ºC for 1 h. The well plate was washed 3 times with wash buffer again, followed by the addition of horseradish peroxidase-labelled detection antibody and incubation at 37 ºC for 30 minutes. The resulting sample was subsequently washed again with wash buffer from the ELISA kit 5 times, followed by the addition of the substrate 3,3',5,5'-tetramethylbenzidine (TMB), incubation at 37 ºC in the dark for 15 min, and then the addition of the stop solution from the ELISA kit to terminate

the reaction. The microplate was then subjected to optical density reading at 540 nm (OD_{540}) with a microplate reader (iMark, Bio-Rad), and the calibration curve of OD_{540} *versus* its concentration was determined using the standard solution provided in the ELISA kit by the vendor. Each trial was performed in triplicate, and the reported results are the averages of two independent trials.

References:

1. Chan, J. M. et al. PLGA–lecithin–PEG core–shell nanoparticles for controlled drug delivery. *Biomaterials* **30**, 1627-1634 (2009).
2. Esim, O., Ozkan, C. K., Sarper, M., Savaser, A. & Ozkan, Y. Development of Gemcitabine Loaded PLGA/Lecithin Nanoparticles for Non-Small Cell Lung Cancer Therapy. *Curr. Drug Deliv.* **17**, 622-628 (2020).
3. Ling, G. et al. Development of novel self-assembled DS-PLGA hybrid nanoparticles for improving oral bioavailability of vincristine sulfate by P-gp inhibition. *J. Control. Release* **148**, 241-248 (2010).
4. Finosh, G. T. & Jayabalan, M. Reactive oxygen species Control and management using amphiphilic biosynthetic hydrogels for cardiac applications. *Adv. Biosci. Biotechnol.* **4,** 1134-1146 (2013).
5. Long, R., Huang, H., Li, Y., Song, L. & Xiong, Y. Palladium-Based Nanomaterials: A Platform to Produce Reactive Oxygen Species for Catalyzing Oxidation Reactions. *Adv. Mater.* **27**, 7025-7042 (2015).
6. Wang, S. et al. Nanoenzyme-Reinforced Injectable Hydrogel for Healing Diabetic Wounds Infected with Multidrug Resistant Bacteria. *Nano Lett.* **20**, 5149-5158 (2020).
7. Wang, Z. et al. Biomimetic nanoflowers by self-assembly of nanozymes to induce intracellular oxidative damage against hypoxic tumors. *Nat. Commun.* **9**, 3334 (2018).
8. Makadia, H. K. & Siegel, S. J. Poly Lactic-co-Glycolic Acid (PLGA) as Biodegradable Controlled Drug Delivery Carrier. *Polymers* **3**, 1377-1397 (2011).
9. Gavini, J. et al. Verteporfin-induced lysosomal compartment dysregulation potentiates the effect of sorafenib in hepatocellular carcinoma. *Cell Death Dis.* **10**, 749 (2019).

10. Lee, I. H. Use of Lecithin As an Antistatic Agent in Nonconductive Crystallization Slurries for Isolating Pure Active Pharmaceutical Ingredients. *Org. Process Res. Dev.* **17**, 1330-1334 (2013).
11. Hunter, C. A. & Jones, S. A. IL-6 as a keystone cytokine in health and disease. *Nat. Immunol.* **16**, 448-457 (2015).
12. Dash, N. R., Khoder, G., Nada, A. M. & Al Bataineh, M. T. Exploring the impact of Helicobacter pylori on gut microbiome composition. *PLoS ONE* **14**, e0218274 (2019).
13. Frost, F. et al. Helicobacter pylori infection associates with fecal microbiota composition and diversity. *Sci. Rep.* **9**, 20100 (2019).
14. Wang, D. et al. Alterations in the human gut microbiome associated with Helicobacter pylori infection. *FEBS Open Bio.* **9**, 1552-1560 (2019).
15. Iino, C. et al. Influence of Helicobacter pylori infection and atrophic gastritis on the gut microbiota in a Japanese population. *Digestion* **101**, 422-432 (2020).
16. Chen, C. C. et al. The interplay between Helicobacter pylori and gastrointestinal microbiota. *Gut Microbes* **13**, 1909459 (2021).
17. Chen, Q. et al. Advanced Sensing Strategies Based on Different Types of Biomarkers toward Early Diagnosis of H. pylori. *Crit. Rev. Anal. Chem*. 1-13 (2023).
18. Kienesberger, S. et al. Gastric Helicobacter pylori Infection Affects Local and Distant Microbial Populations and Host Responses. *Cell Rep.* **14**, 1395-1407 (2016).
19. Zhou, Y. et al. Long-term changes in the gut microbiota after 14-day bismuth quadruple therapy in penicillin-allergic children. *Helicobacter* **25**, e12721 (2020).
20. Yang, L. et al. Helicobacter pylori infection aggravates dysbiosis of gut microbiome in children with gastritis. *Front. Cell. Infect. Microbiol.* **375** (2019).

21. Chen, L. et al. The impact of Helicobacter pylori infection, eradication therapy and probiotic supplementation on gut microenvironment homeostasis: An open-label, randomized clinical trial. *EBioMedicine* **35**, 87-96 (2018).
22. Gao, J.-J. et al. Association between gut microbiota and Helicobacter pylori-related gastric lesions in a high-risk population of gastric cancer. *Front. Cell. Infect. Microbiol.* **8**, 202 (2018).
23. He, C. et al. The eradication of Helicobacter pylori restores rather than disturbs the gastrointestinal microbiota in asymptomatic young adults. *Helicobacter* **24**, e12590 (2019).
24. Iino, C. et al. Infection of Helicobacter pylori and atrophic gastritis influence Lactobacillus in gut microbiota in a Japanese population. *Front. Immunol.* 9, 712 (2018).
25. Hidetaka, Y. et al. Changes in the gut microbiota composition and the plasma ghrelin level in patients with Helicobacter pylori-infected patients with eradication therapy. *BMJ Open Gastroenterol.* **4**, e000182 (2017).
26. Yap, T. W. C. et al. Helicobacter pylori Eradication Causes Perturbation of the Human Gut Microbiome in Young Adults. *PLoS ONE* **11**, e0151893 (2016).
27. Li, M., Jin, X., Liu, T. et al. Nanoparticle elasticity affects systemic circulation lifetime by modulating adsorption of apolipoprotein A-I in corona formation. *Nat. Commun.* **13**, 4137 (2022).
28. LeBel, C. P., Ischiropoulos, H. & Bondy, S. C. Evaluation of the probe 2', 7'-dichlorofluorescin as an indicator of reactive oxygen species formation and oxidative stress. *Chem. Res. Toxicol.* **5**, 227-231 (1992).
