# Supplementary material for: A nanoparticle-based sonodynamic therapy reduces Helicobacter pylori infection in mouse without disrupting gut microbiota
Source: Nat Commun. 2024 Jan 29;15:844. doi: 10.1038/s41467-024-45156-8 (PMC10825188; doi:10.1038/s41467-024-45156-8)
Supplement: Supplementary file 3 — Description of Additional Supplementary Files [file 41467_2024_45156_MOESM3_ESM.docx]

**Description of Additional Supplementary Files**

**File Name: Supplementary Data 1**

**Description:** Effects of antibiotic-based *H. pylori* eradication therapies on human gut microbiota. Supplementary Data 1 contains information including the sample size of human participants, participant age, participant ethnicity, regimen used for antibiotic-based *H. pylori* eradication, sample type, detection methods of *H. pylori* and of gut microbiota, short-term changes (≤ 3 months) (including the control, up-regulated strains, down-regulated strains and diversity changes), long-term changes (6-12 months) (including the control, up-regulated strains, down-regulated strains and diversity changes), long-term changes (≥ 12 months) (including the control, up-regulated strains, down-regulated strains and diversity changes) and references.

**File Name: Supplementary Data 2**

**Description:** Effects of *H. pylori* infection on gut microbiota of human objects and murine animal models. On information from human studies, Supplementary Data 2 contains details including sample size of human participants, participant gender and age, participant ethnicity, BMI index, detection methods of *H. pylori* and of gut microbiota, diseases accompanying the infection, up-regulated strains, down-regulated strains, diversity changes and references. On information from studies using murine animal models, Supplementary Data 2 contains details including sample size of murine animals, infection time, detection time, detection methods of *H. pylori* and of gut microbiota, diseases accompanying the infection, up-regulated strains, down-regulated strains, diversity changes and references.
