# Supplementary material for: A nanoparticle-based sonodynamic therapy reduces Helicobacter pylori infection in mouse without disrupting gut microbiota
Source: Nat Commun. 2024 Jan 29;15:844. doi: 10.1038/s41467-024-45156-8 (PMC10825188; doi:10.1038/s41467-024-45156-8)
Supplement: Supplementary file 6 — Reporting Summary [file 41467_2024_45156_MOESM6_ESM.pdf]

nature portfolio

Corresponding author(s): Lihua Yang

Last updated by author(s): Dec 15, 2023

Reporting Summary

Nature Portfolio wishes to improve the reproducibility of the work that we publish. This form provides structure for consistency and transparency in reporting. For further information on Nature Portfolio policies, see our Editorial Policies and the Editorial Policy Checklist.

Statistics

For all statistical analyses, confirm that the following items are present in the figure legend, table legend, main text, or Methods section.

n/a	Confirmed	
[ ]	[x]	The exact sample size (*n*) for each experimental group/condition, given as a discrete number and unit of measurement
[ ]	[x]	A statement on whether measurements were taken from distinct samples or whether the same sample was measured repeatedly
[ ]	[x]	The statistical test(s) used AND whether they are one- or two-sided<br>*Only common tests should be described solely by name; describe more complex techniques in the Methods section.*
[x]	[ ]	A description of all covariates tested
[x]	[ ]	A description of any assumptions or corrections, such as tests of normality and adjustment for multiple comparisons
[ ]	[x]	A full description of the statistical parameters including central tendency (e.g. means) or other basic estimates (e.g. regression coefficient) AND variation (e.g. standard deviation) or associated estimates of uncertainty (e.g. confidence intervals)
[ ]	[x]	For null hypothesis testing, the test statistic (e.g. F, t, r) with confidence intervals, effect sizes, degrees of freedom and P value noted<br>*Give P values as exact values whenever suitable.*
[x]	[ ]	For Bayesian analysis, information on the choice of priors and Markov chain Monte Carlo settings
[x]	[ ]	For hierarchical and complex designs, identification of the appropriate level for tests and full reporting of outcomes
[x]	[ ]	Estimates of effect sizes (e.g. Cohen's *d*, Pearson's *r*), indicating how they were calculated

Our web collection on statistics for biologists contains articles on many of the points above.

Software and code

Policy information about availability of computer code

Data collection	llumina Hiseq, ImageJ
Data analysis	Origin 8.0; Excel 2016; R 3.6.0; ade4 1.7-13; Graphpad Prism 8.0.1

For manuscripts utilizing custom algorithms or software that are central to the research but not yet described in published literature, software must be made available to editors and reviewers. We strongly encourage code deposition in a community repository (e.g. GitHub). See the Nature Portfolio guidelines for submitting code & software for further information.

Data

Policy information about availability of data

All manuscripts must include a data availability statement. This statement should provide the following information, where applicable:

- Accession codes, unique identifiers, or web links for publicly available datasets
- A description of any restrictions on data availability
- For clinical datasets or third party data, please ensure that the statement adheres to our policy

The authors declare that all data supporting the findings of this study are available within the paper and its Supplementary Information files. Source data is provided. The 16S rRNA sequencing data generated in this study have been deposited in the Genome Sequence Archive (GSA) (https://ngdc.cncb.ac.cn/gsa/) at accession number CRA013610.

Research involving human participants, their data, or biological material

Policy information about studies with human participants or human data. See also policy information about sex, gender (identity/presentation), and sexual orientation and race, ethnicity and racism.

Reporting on sex and gender	No research involving human participants
Reporting on race, ethnicity, or other socially relevant groupings	No research involving human participants
Population characteristics	No research involving human participants
Recruitment	No research involving human participants
Ethics oversight	No research involving human participants

Note that full information on the approval of the study protocol must also be provided in the manuscript.

Field-specific reporting

Please select the one below that is the best fit for your research. If you are not sure, read the appropriate sections before making your selection.

[x] Life sciences [ ] Behavioural & social sciences [ ] Ecological, evolutionary & environmental sciences

For a reference copy of the document with all sections, see nature.com/documents/nr-reporting-summary-flat.pdf

Life sciences study design

All studies must disclose on these points even when the disclosure is negative.

Sample size	For bacterial and cellular assays, each trial was carried out in triplicate, and the reported results are averages of two independent trials. This is a protocol commonly used in the literature (Nat. Commun. 2021, 12, 745; Nat. Commun. 2022, 13, 4137). With each trial performed in triplicate, results from each trial are reliable to acceptable extent. As the reported results are averages of two independent trials, the reproducibility can get confirmed.<br>To examined Ver-PLGA@Lecithin efficacy and biosafety as a sonosensitizer for treating gastric H. pylori infection in mouse models. forty-two C57BL/6J mice were randomly divided into six groups (n = 7 per group), with one group to stay naive throughout the whole study while the rest to receive gastric H. pylori infection and then one of the following treatments: PBS alone, triple therapy, US exposure alone, Ver-PLGA@Lecithin alone, and Ver-PLGA@Lecithin followed by US exposure. In laboratory research work, 7 mice per group is believed to be a sample size big enough for providing results of statistical reliability and for checking reproducibility; all drugs and nanoparticles were dispersed into PBS and administered orally, and the US exposure was applied locally on the skin over stomach. At 48-h after treatment completion, blood samples were collected from 5 mice randomly selected at any pre-specified blood collection time to monitor three pro-inflammatory cytokines and gastric function biomarkers in serum levels and perform complete blood count (CBC) test.<br>In addition, these mice were sacrificed and their stomachs were collected for homogenization and subsequent quantification of H. pylori burden therein. In this way, pro-inflammatory cytokines and gastric function biomarkers in serum levels and CBC analysis are reliable and reproducible. At 48-h after treatment completion, all mice were sacrificed, and the stomachs and the five major organs of 2 mice randomly selected in each group were collected for histology analysis by hematoxylin and eosin (H&E) tissue staining according to the literature (Nat. Commun. 2021, 12, 745), and the rest of 5 mice' stomach and other major organs were homogenized followed by carrying out the bacterial colony-forming units (CFU) counting therein according to the literature (Proc. Natl. Acad. Sci. U. S. A. 2014, 111, 17600-17605). H&E staining is a qualitative analysis, so we randomly select 2 mice from the treatment group as representatives of the group to ensure repeatability. Since CFU counting is a quantitative analysis, we performed CFU counting analysis using an additional 5 mice for each treatment group in an effort to obtain results with acceptable reliability and reproducibility.<br>To examine the biosafety of Ver-PLGA@Lecithin as a sonosensitizer for treating gastric H. pylori infection in mouse models, we randomly selected ten uninfected mice, divided them into two groups (n = 5 per group) and in laboratory research work, 5 mice per group is believed to be a sample size big enough for providing results of statistical reliability and for checking reproducibility. Then, we gave one group orally administered Ver-PLGA@Lecithin, with PBS administered alone for comparison in another, after six consecutive days of administration, their blood samples and major organs were collected to figure out the three pro-inflammatory cytokines in serum levels and H&E staining analysis in Ver-PLGA@Lecithin group. We believe that since H&E staining is a qualitative analysis, we randomly select two mice from the treatment group as representatives of this group, which ensures repeatability.
Data exclusions	No data were excluded from the analysis.
Replication	To verify the reproducibility for bacterial and cellular assays, each trial was carried out in triplicate, and the reported results are averages of two independent trials. The observed results show that all the attempts at replication were successful.<br>To verify the reproducibility for animal experiments, the mouse models in each in vivo assay were randomly allocated into different treatment groups with 7 mice in each group. The observed results show that all the attempts at replication were successful.
Randomization	In each independent assay that involves a bacterial dispersion or a cell culture, the bacterial dispersion or the cell culture was randomly allocated into the control and treatment groups. To ensure randomization, we collected the bacterial or cell culture after it reached

	appropriate state of cell growth, re-dispersed the as-collected bacteria or cells as planktonic objects into an expected medium (either a buffer or a nutrient medium), and then inoculated an expected and equal amount (usually measured in volume) of the resulting bacterial or cell dispersion into each well of a microplate, and the as-inoculated wells were then allocated to the control and different treatment groups. In animal experiments, animals were randomly divided into several groups before any treatment, to ensure the randomness of each group of animals.
Blinding	The investigators of this work were not blinded to group assignment during data collection and/ or analysis. The reasons why blinding was not taken into account in the data collection or analysis in this work are as follows: This work, though involving animal studies, does not have experimental research involving human participants. In clinical studies that involve human participants, investigators are required to be blinded to group assignment during data collection and/or analysis; in contrast, in animal studies of laboratory research works, that investigators are not blinded to group assignment during data collection and/or analysis is a common and normal practice.

Reporting for specific materials, systems and methods

We require information from authors about some types of materials, experimental systems and methods used in many studies. Here, indicate whether each material, system or method listed is relevant to your study. If you are not sure if a list item applies to your research, read the appropriate section before selecting a response.

Materials & experimental systems

n/a	Involved in the study
☒	☐ Antibodies
☐	☒ Eukaryotic cell lines
☒	☐ Palaeontology and archaeology
☐	☒ Animals and other organisms
☒	☐ Clinical data
☒	☐ Dual use research of concern
☒	☐ Plants

Methods

n/a	Involved in the study
☒	☐ ChIP-seq
☒	☐ Flow cytometry
☒	☐ MRI-based neuroimaging

Eukaryotic cell lines

Policy information about cell lines and Sex and Gender in Research

Cell line source(s)	The cell lines used in this study were human gastric adenocarcinoma (AGS) cells and was purchased from Cell Bank of the Chinese Academy of Sciences (Shanghai, China).
Authentication	All the eukaryotic cell lines were used as received without further authentication.
Mycoplasma contamination	The cell lines were not tested for mycoplasma contamination.
Commonly misidentified lines (See ICLAC register)	No commonly misidentified lines were involved in this study.

Animals and other research organisms

Policy information about studies involving animals; ARRIVE guidelines recommended for reporting animal research, and Sex and Gender in Research

Laboratory animals	The animals used in this study are C57BL/6J mice (female, 8-week old). Mice were housed at temperature of 22-25 °C and 12h/12h dark/light cycle. We don't have information on humidity in mouse housing at hand and did not find the related device that can gauge the humidity in the animal center where our animal studies were carried out.
Wild animals	The study did not involve wild animals.
Reporting on sex	Exclusively, mouse models were used as the animal models in this research. The mouse models used in this work were of one sex, which is female. The animal studies in this work did not take into account the effect of sex, as sex-based analysis is commonly not involved in laboratory animal studies.
Field-collected samples	The study did not involve samples collected from the field.
Ethics oversight	The Animal Care and Use Committee at University of Science and Technology of China.

Note that full information on the approval of the study protocol must also be provided in the manuscript.
